# Food–energy–water nexus optimization brings substantial reduction of urban resource consumption and greenhouse gas emissions

**DOI:** 10.1093/pnasnexus/pgae028

**Published:** 2024-01-25

**Authors:** Pengpeng Zhang, Lixiao Zhang, Yan Hao, Ming Xu, Mingyue Pang, Changbo Wang, Aidong Yang, Alexey Voinov

**Affiliations:** State Key Joint Laboratory of Environmental Simulation and Pollution Control, School of Environment, Beijing Normal University, Beijing 100875, China; School of Geographical Sciences, Hebei Normal University, Shijiazhuang 050024, China; State Key Joint Laboratory of Environmental Simulation and Pollution Control, School of Environment, Beijing Normal University, Beijing 100875, China; State Key Joint Laboratory of Environmental Simulation and Pollution Control, School of Environment, Beijing Normal University, Beijing 100875, China; School of Environment, Tsinghua University, Beijing 100084, China; Key Laboratory of Three Gorges Reservoir Region’s Eco-Environment, Ministry of Education, Chongqing University, Chongqing 400044, China; College of Economics and Management and Research Center for Soft Energy Science, Nanjing University of Aeronautics and Astronautics, Nanjing 211106, China; Department of Engineering Sciences, University of Oxford, Oxford OX1 3PJ, UK; Faculty of Engineering Technology, University of Twente, Enschede 7522 NB, The Netherlands

**Keywords:** FEW nexus, integrated optimization, reduction potentials, urban sustainability

## Abstract

Urban sustainability is a key to achieving the UN sustainable development goals (SDGs). Secure and efficient provision of food, energy, and water (FEW) resources is a critical strategy for urban sustainability. While there has been extensive discussion on the positive effects of the FEW nexus on resource efficiency and climate impacts, measuring the extent to which such synergy can benefit urban sustainability remains challenging. Here, we have developed a systematic and integrated optimization framework to explore the potential of the FEW nexus in reducing urban resource demand and greenhouse gas (GHG) emissions. Demonstrated using the Metropolis Beijing, we have identified that the optimized FEW nexus can reduce resource consumption and GHG emissions by 21.0 and 29.1%, respectively. These reductions come with increased costs compared to the siloed FEW management, but it still achieved a 16.8% reduction in economic cost compared to the business-as-usual scenario. These findings underscore the significant potential of FEW nexus management in enhancing urban resource efficiency and addressing climate impacts, while also identifying strategies to address trade-offs and increase synergies.

**Significance Statement**

Our integrated nexus optimization of urban food–energy–water (FEW) systems represents a groundbreaking achievement that has not been accomplished before. The fact that about 75% of global resources are consumed by cities highlights the crucial role of cities in addressing global challenges. In aggregate, the integrated optimization framework we propose aligns with the laws of resource flows in cities. Taking Beijing, one of the world's megacities, as a reference, we provide a valuable benchmark for other cities. Our studies identify the potential for reducing resource consumption and greenhouse gas emissions, which can guide resource management actions to ensure FEW securities. Our results demonstrate the significance of nexus management in enhancing resource efficiency, fostering synergies and mitigating the impacts of climate change.

## Introduction

Rising scarcities of key resources, such as food, energy, and water (FEW), bring formidable challenges to achieving the United Nations sustainable development goals (SDGs) (1, 2). This is particularly the case for cities which are the hubs of resource demand and waste generation (3–5). Challenges of FEW resources are further exacerbated in cities due to their interlinkages and the tele-coupling between the city and its hinterlands (6, 7). Calls for “effective and integrated management” for FEW resources in urban systems from the nexus perspective have increased in frequency and urgency (8).

There is a growing body of literature on the FEW nexus which primarily focuses on characterizing (9–11), modeling (12, 13), and optimizing the FEW nexus (14, 15) at regional, national, and global scales (16–21). However, our understanding of the contributions of the FEW nexus toward SDGs is still limited, particularly regarding the SDGs related to the provision of sufficient FEW for all (SDGs 2, 6, 7), cities (SDG 11), responsible consumption and production (SDG 12), and climate action (SDG 13) (7, 22, 23). Specifically, three critical questions need to be answered: (i) What is the potential of FEW systems in reducing resource-environmental-economic impacts? (ii) Which sectors of FEW systems drive these reductions, and what is their relative importance? Are there trade-offs among these three goals? (iii) What is the added value of the FEW nexus for achieving SDGs in urban systems compared to the “silo” management of FEW resources?

**Fig. 1.**
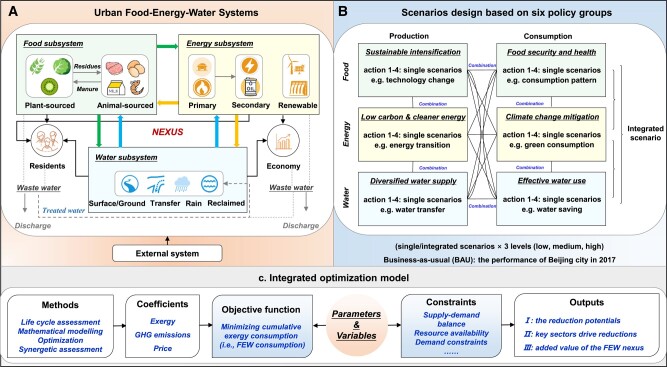
Workflow of the analysis, integrated optimization model, and data sources. This illustration shows urban FEW systems (a), the process of scenario design (b), and the integrated optimization model (c), respectively. Urban FEW systems consist of the system boundary, FEW types, and interconnections among FEW subsystems. The scenarios in this study consider the key actions, such as changes in technologies and production-consumption patterns, in the context of the SDGs, combined with the SSP1-3. The integrated optimization framework is utilized to explore the potential for reducing total cumulative exergy consumption (CExC), GHG emissions, and the associated economic cost under each scenario. Subsequently, a synergetic degree model is developed to assess the performance of the optimization results in improving the synergies of urban FEW systems. Finally, feasible pathways are identified to promote urban FEW sustainability.

To address these questions, we have developed an integrated optimization framework for the urban FEW nexus, combined with a context-based SDGs scenario modeling, to explore the optimal performance of the urban FEW nexus (Figs. 1 and S1–S11). Our approach is based on systematic mathematical modeling and optimization, aiming to identify the best scenario of FEW supply and demand that can meet urban socioeconomic demands while minimizing resource-environmental-economic impacts. We have also taken into consideration the given constraints in each scenario. In this analysis, we have proposed six response policy groups based on FEW production and consumption, which form different scenarios (Fig. 1). These policy groups include: (i) “Sustainable intensification,” (ii) “Food security and health,” (iii) “Low carbon and cleaner energy,” (iv) “Climate change mitigation,” (v) “Diversified water supply,” and (iv) “Efficient water use,” which mainly refer to the shared socioeconomic pathways (SSPs), particularly the SSP1-3 (24, 25). Each policy group includes a set of actions (i.e. decision variables or constraints) that represent the key technologies (e.g. increase the share of renewable energy), production processes (e.g. technical promotion), or consumption patterns (e.g. dietary change) of FEW systems throughout their life cycle processes. The actions within each policy group are mutually exclusive but can be combined in any way. The scenarios created in this study are classified into single and integrated policy groups, categorized under low, medium, and high levels (251 individual scenarios × 3 levels). A brief narrative description of each scenario is outlined in the Scenario construction section and the Tables S1–S8.

One of the key contributions of this study is the utilization of “exergy” (26, 27), as a unified measure of the various forms of FEW resources to offer a comprehensive assessment of resource scarcity and usefulness. Armed with this indicator and integrated optimization model, this study first focuses on minimizing cumulative exergy consumption (CExC) while satisfying urban FEW demands considering the whole life cycle process with regard to different scenarios (28–30). We then evaluated the greenhouse gas (GHG) emissions and the corresponding economic cost based on the optimal results of the minimized CExC in each scenario (31). This optimization framework allows for the evaluation of the benefits of the FEW nexus in achieving SDGs in urban systems, while considering the environmental and economic costs. The sustainability benefits of the urban FEW nexus are demonstrated using Beijing as a case study, providing valuable insights for achieving SDGs in one of the largest cities in the world, and also offer a useful reference for sustainable development of other cities through FEW nexus optimization.

## Results

### Potentials of reducing CExC, GHG emissions, and economic cost

**Fig. 2.**
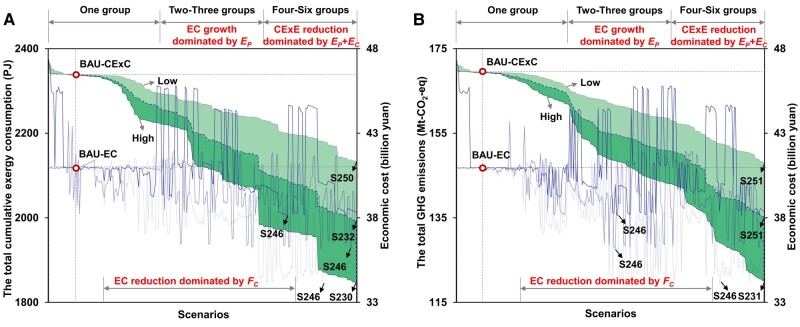
CExC, GHG emissions, and economic cost under different scenarios in Beijing. The optimization results of all scenarios are presented in the figure. In (a), the changes in the optimal cumulative exergy consumption (CExC) and the associated economic cost (EC) under each scenario are shown for each scenario (b), displays the changes in optimal GHG emissions and the associated EC. The stacked plots illustrate the trends of the optimal CExC (a) and optimal GHG emissions (b) under low, mid, and high levels. The curves represent the EC associated with the optimization result of each scenario. EC stands for economic cost. *F_c_*, *E_p_*, and *E_c_* refer to the scenarios based on food consumption, energy production, and energy consumption policy groups, respectively. “EC growth dominated by *E_p_*” indicates that the increase in economic cost is mainly influenced by the scenarios based on energy production policy group. “EC reduction dominated by *F_c_*” signifies that the reduction in economic cost is mainly driven by the scenarios based on food consumption policy group. “CExC reduction dominated by *E_p_* + *E_c_*” implies the reduction in the total cumulative exergy consumption is primarily influenced by the scenarios based on energy production and consumption policy groups.

We have simulated the mitigation trajectories of optimizing CExC and evaluated their related GHG emissions and economic cost in different scenarios categorized as low, mid, and high levels (Fig. 2). Compared to the baseline BAU scenario, our findings indicate that CExC, GHG emissions, and economic cost can be reduced by up to 21% (490.7 PJ, 1 PJ = 10^15^J), 29.1% (49.3 Mt-CO_2_-eq), and 16.8% (68.7 billion yuan), respectively (Figs. S12–S15). These reductions are achieved in scenarios with integrated policy groups under high management intensity. Specifically, the optimal scenarios for CExC, GHG emissions, and economic cost are S230, S231, and S246, which belong to five policy groups (Tables S9 and S10). This indicates the importance of integrated management in achieving synergistic benefits that cannot be achieved individually. It is important to note that the maximal reduction of CExC, GHG emissions, and economic cost occurs in different scenarios, highlighting the trade-offs among these three goals. This also emphasizes the resource-environment-economy trilemma, where no single scenario can simultaneously achieve minimal CExC, GHG emissions, and economic cost.

The potential for saving in CExC, GHG emissions, and economic cost is primarily associated with the consumption-based policy groups (Figs. S16 and S17). When only considering the FEW consumption-based integrated scenarios, the projected reduction in CExC, GHG emissions, and economic cost are 15.9, 15.2, and 15.4%, respectively (Fig. 2). This illustrates the fact that consumption-based policies are expected to be more important for cities as they heavily rely on imported FEW resources (32, 33).

The optimization of CExC and related GHG emissions as well as economic cost, varies greatly across different sectors. The reductions in CExC and GHG emissions are mainly driven by the decarbonization of the energy sector and improvement in energy efficiency. Our analysis shows that the optimal scenarios with integrated energy production and consumption policy groups under the high-level conditions can reduce CExC and GHG emissions by 15.4 and 23.5%, respectively. These reduction potentials are primarily attributed to the shift from petroleum and natural gas-dominated energy sources to low carbon and cleaner energy sources in Beijing's energy structure. However, it is important to note that minimizing resource-environmental impacts does not necessarily minimize economic costs in the energy sector. Trade-offs between resource-environmental impacts and economic cost are evident in scenarios involving the deployment of renewable energy. As the share of renewable energy increases, the total economic cost also rises. In contrast, scenarios related to the food sector demonstrate a decline in economic cost, highlighting the effectiveness of food consumption policies such as sustainable agriculture, and healthier diets. Furthermore, water policy groups are found to be less effective in reducing CExC, GHG emissions, and economic cost, likely due to high dependency of urban water supply and wastewater treatment on the energy sector.

### Nexus contributions to optimizing urban FEW systems

**Fig. 3.**
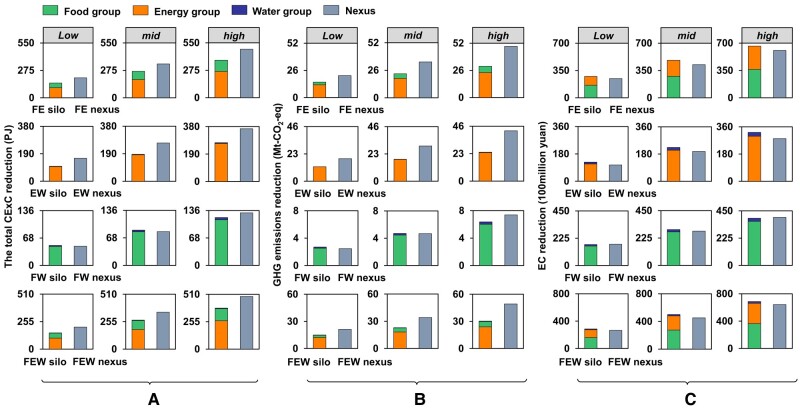
Nexus contributions to reducing CExC, GHG emissions, and the associated economic cost. The figures illustrate the benefits of using the nexus approach to reduce CExC (a), GHG emissions (b), and the associated economic cost (c), compared to the “silo” optimization. We examined optimal scenarios for urban single subsystems (F/E/W group), double subsystems (FE/EW/FE groups) and three triple subsystems (FEW groups). “FE silo” represents the separate optimization of the food and energy subsystems without considering their interconnections. On the other hand, “FE nexus” indicates that the interconnections between these two subsystems were taken into account during optimization. The F/E/W group in Fig. 3 represent the contribution to reducing CExC (a), GHG emissions (b), and associated economic costs (c), when only optimizing the FEW subsystems. The nexus in Fig. 3 indicates the potential reduction in CExC, GHG emissions, and associated economic costs based on the optimal results of the double and triple subsystems which consider the interactions between FEW sectors. The difference values between the double/triple subsystems and the sum of the potential of the corresponding single subsystems (F/E/W group) represent the nexus contribution.

Figure 3 illustrates the maximum potentials of single, double, and triple interactions among FEW sectors in reducing CExC, GHG emissions, and associated economic cost under different levels. It can be found that scenarios considering the nexus between two sectors and among three sectors have a significant impact on achieving resource-environmental optimization objectives, but have a smaller effect on reducing economic costs, compared to scenarios with “silo” management. This suggests that integrated management may lead to an increase in marginal cost. Specifically, scenarios considering the FEW nexus have higher potentials in reducing CExC compared to single FEW policy groups, with reduction of up to 377.3 PJ (76.9%), 227.0 PJ (46.3%), and 484.9 PJ (98.9%) under low, mid, and high levels, respectively. Furthermore, the contributions of the nexus to reducing CExC and GHG emissions increase with more elements and higher management intensities. However, the reductions in economic cost driven by scenarios considering the FEW nexus are lower than those achieved through “silo” optimization. For instance, implementing a full portfolio of FEW policy groups leads to an increase in the contributions of the FEW nexus to the largest reduction potentials of CExC and GHG emissions, from 52.9 PJ (at low level) to 108.5 PJ (at high level), and from 6.3 Mt-CO_2_-eq (at low level) to 19.0 Mt-CO_2_-eq (at high level), respectively. However, compared to “silo” optimization, the contributions of the FEW nexus to reducing economic cost in the optimal scenarios decrease by 2.1 billion yuan (at low level) and 4.4 billion yuan (at high level), respectively.

In light of the recognized significance of the FEW nexus in relation to resource-environmental-economic impacts, we have conducted a further analysis on the characteristics of the nexus between two resources. Specifically, we have examined the interconnections between energy and food/water (FE/EW nexus), which have demonstrated substantial positive effects in reducing CExC and GHG emissions. Our analysis reveals that the scenarios involving the FE nexus and EW nexus can result in a remarkable reduction in CExC, with a maximum decrease of 109.5 PJ and 96.2 PJ, respectively. Additionally, these two nexus also exhibit a significant influence on GHG emissions, leading to a decline of over 17.5 Mt-CO_2_-eq for both scenarios. However, the FE nexus and EW nexus restrain the decline in economic cost, driving the economic cost up by 5.5 billion yuan and 4.1 billion yuan, respectively.

### Roles of integrated management and nexus in improving urban FEW synergies

**Fig. 4.**
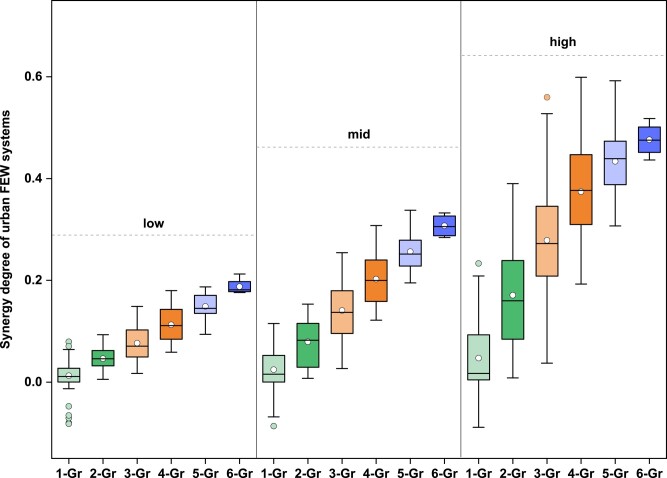
Ranges of synergetic degree for urban FEW systems. The distribution of the synergetic degree of urban FEW systems is evaluated based on the optimization results of each scenario at low, mid, and high levels. The FEW subsystems are divided into six parts, which include food production and consumption, energy production and consumption, and water production and consumption. The scenario design also includes six production and consumption-based policy groups. Therefore, 1- and 2-Gr represent the scenarios with one policy group and two policy groups, respectively, and so on.

To gain a comprehensive understanding of integrated optimization from the nexus perspective, we have developed a synergetic assessment model (SAM) to explore the changes in urban FEW systems. The synergetic degree of urban FEW systems in each scenario is evaluated based on the aforementioned optimization results. As illustrated in Fig. 4, more than 90% of the scenarios perform considerably better than the BAU scenario. The synergetic degree of urban FEW systems shows a clear upward trend driven by the integrated policy groups and high management intensity. The results reveal that integrated optimization coupled with the expansion of policy groups can enhance synergies and reduce trade-offs in urban FEW systems. On average, compared to the BAU scenario, the synergetic degrees of urban FEW systems under low, mid, and high levels reach 0.19, 0.31, and 0.48, respectively. This implies that higher level of integrated management facilitates the transition of urban FEW systems from disorder to order, and even to a steady state. The relative contributions of scenarios with different policy groups range from 0.01 to 0.05 (one policy group), 0.05 to 0.17 (two policy groups), 0.08 to 0.28 (three policy groups), 0.11 to 0.37 (four policy groups), 0.15 to 0.43 (five policy groups), and 0.19 to 0.48 (six policy groups).

It must be pointed out that continuously increasing the elements in the integrated scenarios does not necessarily maximize the synergetic degree of urban FEW systems. For instance, under midlevel, the largest synergetic degree in the scenario with five policy groups (except water production) is up to 0.34, while that in the scenario with six policy groups (FEW production and consumption) is only 0.31. The trend becomes more notable under high level. The largest synergetic degrees in scenarios with four and five policy groups are higher than that in scenarios with six policy groups. These results indicate that we should aim for a balance among the three objectives for relative optimizations, rather than solely focusing on minimizing one of the objectives.

## Discussion

The management of the urban FEW nexus should prioritize options that maximize synergies, improve resource-use efficiency, internalize environmental and economic impacts, and minimize trade-offs (34). Optimizing urban FEW systems is a promising and critical option to ensure resource security and minimize environmental-economic impacts (35). Given the openness of urban systems, the interactions and interplay of multiple sectors and processes critically influence resource consumption, environmental impacts, and the corresponding economic cost. In this study, we propose to optimize urban FEW systems from a nexus and life cycle-based perspective.

As mentioned above, the current FEW nexus conceptual frameworks often concentrate on specific places or contexts, limiting their ability to capture socioeconomic and environmental interactions across different locations. In this study, we propose an expanded nexus conceptual framework that takes into account the multisectoral (FEW), multisystematic (resource, economy, and society), and multiregional (city and its hinterlands) perspectives. Such integrated approach is more applicable and can address nexuses in multiple places as well as the reallocation of resource production caused by the increasing separation between resource production and consumption. Additionally, most studies on FEW nexus optimization and simulation have primarily focused on biophysical and technical aspects, with few quantifying the contributions of the nexus to progress toward meeting the SDGs in urban systems (19, 22). Therefore, this optimization work also considers the socioeconomic aspects and evaluates the performance of the nexus in delivering the SDGs in urban systems, going beyond the traditional “silo” optimization of FEW resources. Furthermore, understanding and identifying synergies and trade-offs among sectors can maximize overall system resource efficiency and ensure environmental sustainability while meeting the basic needs of urban socioeconomic development.

### There is significant potential to reduce resource-environmental-economic impacts

Our research demonstrates the practicality of applying the nexus concept to the SDGs, and integrated nexus optimization can generate substantial benefits. This is particularly important in urban areas, which have been recognized as central to driving the SDGs (31) and are increasingly at the center of the FEW nexus discussion (36). For instance, in comparison to the BAU scenario, the highest potentials of the reduction in CExC, GHG emissions, and economic cost in Beijing are all over 16%, even reach to 30%. The FEW nexus offers additional benefits that outweigh the traditional isolated optimization methods. Among the highest potential outcomes, the FEW nexus provides large added value that its contributions to reducing CExC and GHG emissions reach to 22.1 and 38.6%, respectively. Moreover, these positive outcomes are driven by integrated scenarios involving multiple policy groups under high management conditions. Consequently, our findings are crucial for understanding the importance of coordinating and cooperating across sectors and systems to promote sustainable urban development in terms of FEW resources.

Greater emphasis should be placed on implementing consumption-side policies in urban areas as an effective option to decrease FEW demands while minimizing environmental effects and corresponding economic cost. This study found that an integrated scenario focusing solely on a FEW consumption could account for over 50% of the potential reduction in CExC, GHG emissions, and economic costs in Beijing. The changes in a FEW consumption also demonstrated significant difference in their resource-environmental-economic impacts. For example, the scenarios with energy consumption policy groups have the larger contributions to reducing CExC and GHG emissions compared to other scenarios, while scenarios with food consumption policy groups played a dominant role in mitigating economic cost. It is important to note that shifting toward cleaner and new energy sources (as seen in the scenarios with energy production policy groups) can also be an effective approach to decrease CExC and GHG emissions, which is associated with their biophysical properties.

### No easy answer to minimize the resource-environmental-economic impacts simultaneously

Solutions to the resource-environmental-economic trilemma are not easily found (37, 38). Generally, integrated optimization with more sectors under higher management intensity leads to larger positive resource-environmental-economic outcomes. However, the scenarios with the most sectors may not always result in the best outcomes. For example, in our analysis, the optimal scenarios with minimal CExC, GHG emissions, and the associated economic costs are the S230, S231, and S246, respectively. These findings suggest that integrated optimization can create stronger synergies across urban FEW systems. However, it is not a cure-all as adding sectors inevitably increases system complexity, which generally incurs higher economic costs compared to “silo” optimization. In addition, the change from a groundwater-dominated supply structure to diversified water sources (e.g. reclaimed water and cross-regional water diversion) increases energy use and creates trade-offs between sustainable water supply and energy conservation. This highlights the need for policy coherence and innovative responses in resource management (39). Therefore, achieving urban FEW sustainability requires not only technical solutions, but also complex interactions among stakeholders, including government, private sector, experts, and citizens. Addressing these challenges will necessitate additional research, time, and financial resources.

The integrated optimization model developed in this study, based on the nexus perspective, can be applied to other cities facing similar sustainability challenges. Such optimization analysis is crucial for urban development in order to meet the SDGs. However, it is important to acknowledge that the model presented in this study has been developed based on various methodological assumptions to simplify the complex interconnections of the real world. We have only optimized the current status in Beijing in 2017 and assume that certain socioeconomic factors remain unchanged including population volume and structure, the output of each economic sector, FEW resource endowments, and economic cost of each technology. Given the diversities of FEW sources in Beijing, we have assumed that the key provision regions of Beijing's FEW resources remain the same. Due to data limitations, we were not able to divide the FEW resources into more detailed types. These assumptions are reasonable for the current focus, but should be revisited in future work based on more detailed data. Nevertheless, the proposed framework can still be adjusted to integrate other factors into the optimization framework. Another limitation of this work is that the optimization process is entirely based on mathematical programming. Although stakeholders can participate in developing the optimization model, they are only presented with the final results, making it difficult for them to understand the impacts of FEW interactions (40, 41). Further research aims to use relatively straightforward mathematical techniques to solve complex optimization problems. In addition, we have used the multiple scenarios with different levels to perform sensitivity and uncertainty analysis, in order to make our results more robust. The detailed information is presented in Section S4 and Figs. S18–S21.

In conclusion, our results bear out the need, potential benefits and trade-offs of employing integrated nexus approach to address global challenges, particularly in the context of urban FEW sustainability. Our study reveals that compared to the BAU scenario, integrated FEW resource management has the potential to achieve significant reductions in CExC (up to 21.0%), GHG emissions (up to 29.1%), and economic costs (up to 16.8%). However, implementing this approach requires substantial adjustments to urban FEW systems, especially consumption-side transitions. What's more, it is crucial to consider the interactions among FEW sectors and the tele-connection between cities and their hinterlands. Our analysis reveals that the added value of the FEW nexus can contribute to a reduction of 21.2 and 38.6% in CExC and GHG emissions within best scenario, respectively. However, it has a comparatively smaller impact on reducing economic costs compared to the “silo” management approach. Overall, while the nexus concept helps overcome the limitations of compartmentalized thinking, its implementation is challenging. Further research should explore scenarios in a more detailed and comprehensive manner, considering factors such as climate change, socioeconomic developments, management policies, natural disasters, human and infrastructural investment, and path-dependency. Such in-depth analysis can facilitate sustainable urban development and contribute to the achievement of SDGs on a broader scale.

## Materials and methods

### Integrated optimization framework

An integrated optimization framework has been developed that combines the mathematical optimization model with life cycle assessment (LCA) model, exergy-environmental-economic cost coefficients and SAM. The framework consists of four interconnected modules. First, a systematic mathematical modeling-based optimization framework is constructed, consisting of a preliminary stage and a simultaneous design stage. The preliminary design stage focuses on the individual FEW subsystems and the important life cycle processes within these subsystems. The simultaneous design stage considers the possible interlinkages among these subsystems, and the tele-coupling between the city and its hinterlands (40) (see the Section S1). Second, a series of scenarios are set for the changes in different actions (e.g. production or treatment technological measures) and resource types of FEW production and consumption subsystems. The key assumptions and important deducing processes are described in Section S2 (module 2). Third, key parameters, variables, objective functions, and constrains are established to describe the technical constraints, behavior of each scenario, and the interconnections among FEW subsystems. The optimization model is then executed with the aim of minimizing cumulative exergy consumption (i.e. minimizing resource consumption) of each scenario to achieve integrated optimal management (step 1). It should be noted that this objective function is to minimize the total resource consumption, but it does not necessarily mean that the resource consumption of each subsystem is minimized. The demand of each subsystem for the other two subsystems and the characteristics of the supply from one subsystem to the others are unknown and will be determined through optimization. Furthermore, we quantify the environmental impacts (e.g. the GHG emissions) and economic costs based on the optimization results obtained from different scenarios (step 2). By comparing the total cumulative exergy consumption (CExC), GHG emissions, and economic cost of each scenario, we can identify the potential optimal pathway for urban FEW systems (module 3). Finally, we analyze the changes in the synergetic degree of urban FEW systems on the basis of the optimization scheme of each scenario (module 4).

### Scenario construction

#### Baseline scenario

The status of Beijing city's FEW systems in 2017 was used as the baseline scenario (BAU) in our analysis. The BAU scenario described the performance of key actions, including the amounts of FEW supply and demand, technology, the CExC of the total/subsystems, economic cost, and interlinkages among FEW systems. The baseline scenario serves as a reference point for comparing with other scenarios to identify the optimal pathways for urban sustainable development. The important outcomes of the baseline scenario can be found in Section S3.

#### Alternative scenario

**Fig. 5.**
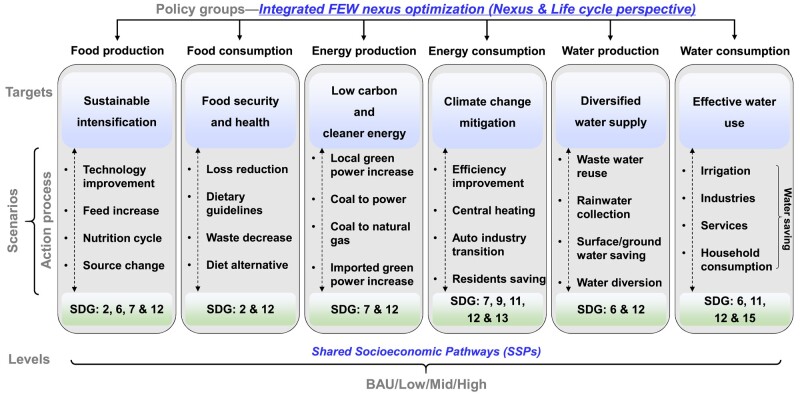
Schematic diagram of the construction of urban FEW sustainability. We begin with six policy groups, each including a specific subset of FEW systems to address the targets related to FEW sustainability. Additionally, four actions are suggested to facilitate the important processes associated with each subset of FEW systems thereby promoting the achievement of SDGs relevant to FEW resources. Furthermore, referring to the shared SSPs, especially SSPs 1–3, we propose a hypothesis that explores different degrees of changes in political, life-style, economic, and technological aspects: low, medium, and high levels.

A series of alternative scenarios were developed here to explore the reduction potentials of urban resource consumption-GHG emissions-economic cost (Fig. 5). These scenarios involve six single policy groups that connect with the production and consumption of FEW subsystems. The targets of these policy groups align closely with the SDGs, including zero hunger (SDG 2), clean water and sanitation (SDG 6), affordable and clean energy (SDG 7), sustainable cities and communities (SDG 11), responsible consumption and production (SDG 12), and climate action (SDG 13). These scenarios provide a starting point for comprehensive analyses. More importantly, each of the six subsystem-policy groups is further divided into four action processes, which aim to describe the impacts of key life cycle stages (e.g. changes in production technologies and consumption-patterns) on urban FEW systems (full description in Section S2).

Second, these single policy groups were randomly combined to create integrated scenarios, where all action processes from each single policy group were applied. In other words, integrated scenarios consist of two or more single policy groups. This approach allows for the systematic combination of different single policy groups to form integrated optimization strategies as far as possible, which can also correspond to the targets of the SDG agenda (42).

Finally, each scenario was combined with the development modes of three levels, which are based on the SSPs, especially the SSP1-3 (43). In addition, we implemented the options by changing the model parameters, assuming no changes in population and economic growth.

### Optimization model

#### Step 1: minimizing the total cumulative exergy consumption

Mathematically, the objective of each scenario for urban FEW systems can be solved within the integrated optimization framework and under the constraints of internal-, and cross- systems (i.e. a set of linear constraints and one nonlinear constraint). The mathematical form is presented in Sections S5 and S6 (Key objective functions and constrains).


(1) $$\mathrm{Minimize}\;CExC_{\mathrm{total}}^{\mathrm{scenario}}=CExF^{\mathrm{scenario}}+CExE^{\mathrm{scenario}}+CExW^{\mathrm{scenario}}$$

where $CExC_{\mathrm{total}}^{\mathrm{scenario}}$ represents the total cumulative exergy consumption of each scenario. $CExF^{\mathrm{scenario}}$, $CExE^{\mathrm{scenario}}$, and $CExW^{\mathrm{scenario}}$ indicate the cumulative exergy consumption for urban food, energy, and water subsystems in the scenario, respectively.

##### Subject to (1) constraints within FEW subsystems

- FEW supply demand balance: for urban system, the FEW resource demands should be met by the sum of local production and imported from other provinces in China.
- For food subsystem, food supply from local and other provinces in China should consider the loss during harvest and processing as well as the waste. The intake range of each food type should adhere to the minimum and maximum value specified in Chinese Dietary Guidelines. The availability of agricultural land does not exceed the current total amount.
- For energy subsystem, the availability of raw energy material does not exceed the current viability.
- For water subsystem, the withdraw of surface and ground water should not exceed the actual availability in Beijing in 2017. In addition, we also consider the water quality issues and choose chemical oxygen demand (COD) level as the indicator. The concentration balance with COD levels in wastewater treatment plants can be referred to Leung Pah Hang et al. (44).

##### Subject to (2) constraints across-subsystems

Energy is required for food and water production. Water is also needed for food and energy production. For instance, the whole life cycle processes in food subsystem (e.g. agricultural production, livestock feeding, processing, transport, and cooking) need energy and water inputs.

The reduction potential of the total cumulative exergy consumption (CExC) in each scenario can be calculated by deducting the total (CExC) of BAU by that of each scenario,


(2) $$RP_{CExC}=CExC_{BAU}-CExC_{\mathrm{total}}^{\mathrm{scenario}}$$

where $RP_{CExC}$ is the reduction potential of the total cumulative exergy consumption in each scenario. $CExC_{BAU}$ is the total cumulative exergy consumption of the baseline scenario (BAU).

#### Step 2.1: Process-based LCA of the GHG emissions

Methods, like LCA, have yielded important insights into evaluating the environmental impacts (45, 46). LCA is a well-established technique used to measure the environmental impact of a product or service at every stage of its life cycle (47, 48). In this study, the GHG emissions can be calculated with the aid of the process-based LCA (PLCA) model. As to urban FEW subsystems, their key stages considered in the whole life cycle are diverse, corresponding to the system boundaries (city and its hinterlands) and key processes in the integrated optimization framework, respectively.

In the food subsystem, the accounting focuses on four stages during the life cycle, i.e. production (tillage, sow, irrigation, fertilization, pesticide, and harvest), processing (grain threshing and slaughter), transport, and consumption (cooking). Additionally, the emissions from ruminant processes and dung need to be considered for animal-sourced foods. In the energy subsystem, the well-to-gate PLCA model for primary fossil energy also includes four processes, such as energy extraction, processing, transmission, and combustion. To measure the GHG emissions from thermal power and heating, we extended the above system boundary to include power generation. For renewable energy, the system boundary was defined to encompass two stages: construction (raw material acquisition and module assembly) and power generation. In the water subsystem, a cradle-to-cradle PLCA model was conducted, involving the abstraction, treatment and distribution, end use, and wastewater treatment. The total GHG emissions of urban FEW systems can be calculated by the following equations:


(3) $$\begin{array}{rl} & \mathrm{GHG}=Q_{i}^{f}+Q_{j}^{e}+Q_{k}^{w}\\ & \;=\sum_{i=1}^{n}M_{i}^{f}\xi_{i}^{s}+\sum_{\;j=1}^{m}M_{j}^{e}\xi_{j}^{s}+\sum_{k=1}^{l}M_{k}^{w}\xi_{k}^{s} \end{array}$$

where GHG is the total GHG emissions (t CO_2_-eq); the $Q_{i}^{f}$, $Q_{j}^{e}$, and $Q_{k}^{w}$ are the GHG emissions of FEW subsystems, respectively, and *i*, *j*, and *k* represent the different categories of FEW resources, respectively. $M_{i}^{f}$, $M_{j}^{e}$, and $M_{k}^{w}$ are the amounts of FEW consumption, respectively. And $\xi_{i}^{s}$ is the GHG emission coefficients of different life cycle processes.

The reduction potential of GHG emissions in each scenario are calculated by deducting GHG emissions of BAU by that of each scenario:


(4) $$RP_{\mathrm{GHG}}=\mathrm{GH}G_{\mathrm{BAU}}-\mathrm{GH}G_{\mathrm{scenario}}$$

where $RP_{\mathrm{GHG}}$ is the reduction potential of GHG emissions in each scenario. $\mathrm{GH}G_{\mathrm{BAU}}$ and $\mathrm{GH}G_{\mathrm{scenario}}$ are the GHG emissions of the baseline scenario and alternative scenarios, respectively.

#### Step 2.2: Economic cost of each scenario

To calculate the economic cost of urban FEW systems associated with different optimization pathways, we use the prices of FEW resources based on that of the BAU, which are then multiplied by the amounts of FEW demands. The economic cost of each scenario can be defined by


(5) $$\begin{array}{rl} & \mathrm{Econ}C_{\mathrm{total}}=\mathrm{Econ}C_{i}^{f}+\mathrm{Econ}C_{j}^{e}+\mathrm{Econ}C_{k}^{w}\\ & \;\;\;=\sum_{i=1}^{n}D_{i}^{f}P_{i}^{f}+\sum_{\;j=1}^{m}D_{j}^{e}P_{j}^{e}+\sum_{k=1}^{l}D_{k}^{w}P_{k}^{w} \end{array}$$

where $\mathrm{Econ}C_{\mathrm{total}}$ is the total economic cost based on the optimization scheme of each scenario. $\mathrm{Econ}C_{i}^{f}$, $\mathrm{Econ}C_{j}^{e}$, and $\mathrm{Econ}C_{k}^{w}$ are the economic cost of FEW subsystems, respectively. $D_{i}^{f}$, $D_{j}^{e}$, and $D_{k}^{w}$ denote the optimal FEW demand of each scenario, respectively. $P_{i}^{f}$, $P_{j}^{e}$, and $P_{k}^{w}$ are the corresponding prices of FEW resources (*i*, *j*, and *k*).

The reduction potential of the total economic cost in each scenario are calculated by deducting the total economic cost of BAU by that of each scenario:


(6) $$RP_{\mathrm{TEC}}=\mathrm{TE}C_{\mathrm{BAU}}-\mathrm{TE}C_{\mathrm{scenario}}$$

where $RP_{\mathrm{TEC}}$ represents the reduction potential of the total economic cost in each scenario. $\mathrm{TE}C_{\mathrm{BAU}}$ and $\mathrm{TE}C_{\mathrm{scenario}}$ are the total economic cost of the baseline scenario and the alternative scenarios, respectively.

#### Synergetic assessment model

The interactions among FEW sectors are complex and dynamic, resulting in diverse states such as synergies and trade-offs (49). To describe the state of urban FEW systems, a SAM is established based on the order parameters and slaving principle of synergetic theory proposed by Haken (50). The assessment results indicate the degree of mutual cooperation and support among FEW systems under different scenarios (i.e. the steady state of urban FEW systems). Furthermore, this model also allows for quantifying the contribution of the nexus on urban FEW synergies.

There are two important aspects that need to be emphasized before building the SAM. First, the system boundary used to quantify the synergetic degree is the same as the integrated optimization framework. The data required for this assessment are obtained from the optimization results of all scenarios. Second, cities are at the center of global resources consumption (36), and FEW resources are not only connected to, but also dependent on, each other (51). Therefore, it is crucial to pay more attention to the effects of integrated management on building urban FEW synergies. The supplies and consumptions of these three resources, and the CExC of one subsystem caused by the other two subsystems (e.g. energy for food and water subsystems) have a significant impact on the status of urban FEW nexus. Therefore, these indicators are selected as the order parameters of urban FEW systems.

*(1) Identifying the order parameters.* In the complex system, each subsystem is defined as $S=\{S_{1},S_{2},\ldots ,S_{i}\}$, i=1,2,…,n. In this study, urban FEW systems can be described as $S=\{S_{1},S_{2},S_{3}\}$, where $S_{1}$, $S_{2}$, and $S_{3}$ represent food, energy, and water subsystems, respectively. For each subsystem, $S_{i}=\{s_{i1},s_{i2},\ldots ,s_{ij}\}$, $s_{ij}$ denotes the order parameter sets of the $S_{i}$ subsystem. Supposing n≥2, $\beta_{ij}\leq s_{ij}\leq \alpha_{ij}$, j∈[1,n], where $\alpha_{ij}$ and $\beta_{ij}$ are the maximum and minimum value of order parameter, respectively. We set the planning value or modeling value based on scenarios as the maximum values, and the value obtained from the reference year (2017) or modeling value based on scenarios as the minimum values. According to the synergetic theory, the order parameters can be divided into positive and negative variables. When the component is positive, the larger value has better impact on urban FEW systems. In contrast, if the component is negative, the less value has better impact on urban FEW systems.

*(2) Calculating the order degree.* Assuming that there exist the positive ($\bar{\;p}$) and negative ($\bar{q}$) components, the calculation functions are given below:


(7) $$f_{i}(s_{ij})=\left\{\begin{array}{c} \frac{s_{ij}-\beta_{ij}}{\alpha_{ij}-\beta_{ij}}\;s_{ij}\in \bar{\;p}\\ \frac{\alpha_{ij}-s_{ij}}{\alpha_{ij}-\beta_{ij}}\;s_{ij}\in \bar{q} \end{array}\right\}$$

where $f_{i}(s_{ij})$ is the order degree of component, $f_{i}(s_{ij})\in [0,1]$. The value of order degree is closer to 1, implying that the “contribution” of order parameter on subsystem is higher. The order degree of order parameter $f_{i}(s_{ij})$ can be evaluated by the total contribution of the degree of components. Generally, it is done using the geometric weight sum, which is expressed as the formula (8)


(8) $$f_{i}(s_{i})=\sqrt[{n}]{\prod_{i=1}^{n}\;f_{i}(s_{ij})}$$

where larger value of $f_{i}(s_{i})$ also has larger contribution on the order degree of urban FEW systems. On the contrary, less value of $f_{i}(s_{i})$ has less contribution on the order degree of urban FEW systems.

*(3) Establishing the SAM .* Given that the order degree of urban FEW systems at times $t_{0}$ (in 2017) and $t_{i}$ are $f_{i}t_{0}(s_{i})$ and $f_{i}t_{t}(s_{i})$, respectively, the assessment functions are given below:


(9) $$\mathrm{SAM}=\theta \sqrt[{n}]{\left|\prod_{i=1}^{n}\left[\;f_{i}t_{t}(s_{i})-f_{i}t_{0}(s_{i})\right]\right|}$$

(10) $$\theta =\frac{\underset{i}{\min}\;[\;f_{i}t_{t}(s_{i})-f_{i}t_{0}(s_{i})\neq 0]}{\left|\underset{i}{\min}\;[\;f_{i}t_{t}(s_{i})-f_{i}t_{0}(s_{i})\neq 0]\right|}\;i=1,2,3$$

where SAM∈[−1,1], the value of SAM is closer to 1, indicating that the synergetic degree of urban FEW systems is higher.

#### Data sources

The basic data of FEW consumption and population in Beijing in 2017 (referred to as the BAU scenario) was collected from the Beijing Statistical Yearbook and bulletins (52). Information of FEW availabilities from local and external systems were taken from the China Statistical Yearbook, Provincial Statistical Yearbook, and previous studies (53, 54). The detailed data and related parameters mentioned in this study can be found in Section S7.

To depict the dietary status in Beijing, seven food types were evaluated based on diet habits and preferences. These food types include grains (wheat and rice), vegetables, fruits, meat (pork, beef, and poultry), eggs, aquatic products, and dairy. The National Cost-Benefit Compilation of Agricultural Products (55) provided the material and energy inputs for agricultural production (such as pesticides, fertilizer, and feed) for each food category per province. For the energy subsystem, data on primary fuel energy (e.g. raw coal, crude oil, and natural gas), renewable energy (e.g. hydropower, solar power, wind power, and biomass energy), and secondary energy (e.g. thermal power and heating) were used. Energy supply demand data were sourced from the China Energy Statistical Yearbook and Beijing Statistical Yearbook in 2018 (52, 56). As to the water subsystem, data on surface water, groundwater, rainwater and water transfer were obtained from the Beijing Statistical Yearbook and Beijing Water Statistical Yearbook in 2018 (52, 57).

The GHG emission factor for each resource during the life cycle processes were derived from the Ecoinvent database version 3.0, and the characterization factors of China's practices were primarily used. The LCA model used to calculate GHG emissions is ReCipe Midpoint (global warming potential on a 100-yeat basis, CO_2_-eq), as a standard in scientific LCA research (58). The exergy coefficients for different resources were gathered from previous studies (27, 59–61).

## Supplementary Material

pgae028_Supplementary_Data

## Data Availability

The data that support the findings of this paper are available in the Supplementary material. Source data are provided with this paper. The codes for data processing are conducted in Matlab (R2020b), and data illustration is generated in Origin 2021.
