# Supplementary material for: Food–energy–water nexus optimization brings substantial reduction of urban resource consumption and greenhouse gas emissions
Source: PNAS Nexus. 2024 Jan 25;3(2):pgae028. doi: 10.1093/pnasnexus/pgae028 (PMC11079490; doi:10.1093/pnasnexus/pgae028)
Supplement: pgae028_Supplementary_Data [file pgae028_supplementary_data.docx]

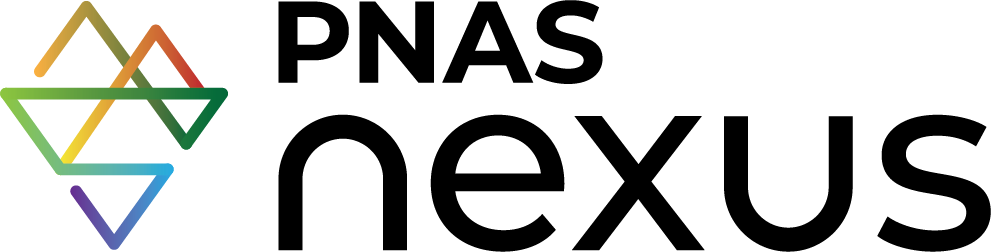


**Supplementary Materials for**

**Food-energy-water nexus optimization brings substantial reduction of urban resource consumption and greenhouse gas emissions**

Pengpeng Zhang^a,b^, Lixiao Zhang^a,1*^, Yan Hao^a^, Ming Xu^c,2*^, Mingyue Pang^d^, Changbo Wang^e^, Aidong Yang^f^, Alexey Voinov^g^

^a^State Key Joint Laboratory of Environmental Simulation and Pollution Control, School of Environment, Beijing Normal University, Beijing 100875, China.

^b^School of Geographical Sciences, Hebei Normal University, Shijiazhuang 050024, China. ^c^School of Environment, Tsinghua University, Beijing 100084, China.

^d^Key Laboratory of Three Gorges Reservoir Region's Eco-Environment, Ministry of Education, Chongqing University, Chongqing 400044, China.

^e^College of Economics and Management & Research Center for Soft Energy Science, Nanjing University of Aeronautics and Astronautics, Nanjing 211106, China.

^f^Department of Engineering Sciences, University of Oxford, Oxford OX1 3PJ, UK.

^g^Faculty of Engineering Technology, University of Twente, Enschede 7522 NB, Netherlands.

**^*^Corresponding authors.** Lixiao Zhang, Ming Xu

**Email:** [zhanglixiao@bnu.edu.cn](mailto:zhanglixiao@bnu.edu.cn) (Lixiao Zhang); [xu-ming@tsinghua.edu.cn](mailto:xu-ming@tsinghua.edu.cn) (Ming Xu)

**This PDF file includes:**

Supporting text

Figs. S1 to S21

Tables S1 to S24

SI References (1 to 36)

**Contents**

[**1. Details of the integrated optimization model** 5](#_Toc155882908)

[**Supplementary Fig. S1.** Systematic methodological optimization framework for urban system 5](#_Toc155882909)

[**Supplementary Fig. S2.** Constructing and solving an integrated optimization model 6](#_Toc155882910)

[**Supplementary Fig. S3.** Illustrative the generic superstructure of integrated subsystems 7](#_Toc155882911)

[**Supplementary Fig. S4.** Accounting framework of urban food subsystem in integrated optimization model 8](#_Toc155882912)

[**Supplementary Fig. S5.** Model details for urban food subsystem 9](#_Toc155882913)

[**Supplementary Fig. S6.** Accounting framework of urban energy subsystem in integrated optimization model 10](#_Toc155882914)

[**Supplementary Fig. S7.** Model details for urban energy subsystem (types and the well-to-gate) 11](#_Toc155882915)

[**Supplementary Fig. S8.** Accounting framework of urban water subsystem in integrated optimization model 12](#_Toc155882916)

[**Supplementary Fig. S9.** Model details for urban water subsystem 13](#_Toc155882917)

[**Supplementary Fig. S10.** The nexus among FEW and socio-economic system in integrated optimization model 14](#_Toc155882918)

[**Supplementary Fig. S11.** The details of optimization model for urban FEW systems 15](#_Toc155882919)

[**2. Constructing scenarios associated with the SDGs** 16](#_Toc155882920)

[**Supplementary Table S1.** The changes in key action processes under -, mid-, and high- levels of urban food production subsystem 17](#_Toc155882921)

[**Supplementary Table S2.** The major food supply regions for Beijing city^b^ 18](#_Toc155882922)

[**Supplementary Table S3.** The changes in key action processes under -, mid-, and high- levels of urban food consumption subsystem 20](#_Toc155882923)

[**Supplementary Table S4.** The Chinese Food Guide Pagoda (2016) recommended by the Chinese Dietary Guidelines (g/cap ∙ day) 21](#_Toc155882924)

[**Supplementary Table S5.** The changes in key action processes under -, mid-, and high- levels of urban energy production subsystem^e^ 23](#_Toc155882925)

[**Supplementary Table S6.** The changes in key action processes under -, mid-, and high- levels of urban energy consumption subsystem^f^ 25](#_Toc155882926)

[**Supplementary Table S7.** The changes in key action processes under -, mid-, and high- levels of urban water production subsystem^g^ 27](#_Toc155882927)

[**Supplementary Table S8.** The changes in key action processes under -, mid-, and high- levels of urban water consumption subsystem^h^ 29](#_Toc155882928)

[**Supplementary Table S9.** The description of scenarios in single policy group 30](#_Toc155882929)

[**Supplementary Table S10.** The description of scenarios in integrated policy groups 32](#_Toc155882930)

[**3. Results to support the main conclusion** 34](#_Toc155882931)

[**Supplementary Fig. S12.** The total cumulative exergy consumption (CExC) and the FEW nexus of baseline scenario (BAU) in Beijing, 2017 34](#_Toc155882932)

[**Supplementary Fig. S13.** The per capita food consumption and water use from different sources in the BAU scenario, compared with the recommend standards and the upper limit of water utilization 35](#_Toc155882933)

[**Supplementary Fig. S14.** The total CExC and economic cost of different integrated policy groups 36](#_Toc155882934)

[**Supplementary Fig. S15.** GHG emissions and economic cost of different integrated policy groups 37](#_Toc155882935)

[**Supplementary Fig. S16.** The total cumulative exergy consumption (CExC) and economic cost of six single policy groups 38](#_Toc155882936)

[**Supplementary Fig. S17.** GHG emissions and economic cost of six single policy groups 39](#_Toc155882937)

[**4. The uncertainty and sensitivity analysis of our results** 40](#_Toc155882938)

[**Supplementary Fig. S18.** The GHG emissions and economic cots of baseline scenario (BAU) in Beijing, 2017 41](#_Toc155882939)

[**Supplementary Fig. S19.** Uncertainty analysis of the optimization results (the total CExC) associated with single and integrated policy groups at low, mid and high levels 42](#_Toc155882940)

[**Supplementary Fig. S20.** Uncertainty analysis of the optimization results (GHG emissions) associated with single and integrated policy groups 43](#_Toc155882941)

[**Supplementary Fig. S21.** Uncertainty analysis of the optimization results (economic cost) associated with single and integrated policy groups 44](#_Toc155882942)

[**5. The explanation of key parameters and variables** 45](#_Toc155882943)

[**6. Key objective functions and constrains in this work** 48](#_Toc155882944)

[**7. Data sources and the key parameters of each resource in the different life cycle processes^i^** 53](#_Toc155882945)

[**Supplementary Table S11.** The key materials and energy input intensities of grain in the different life cycle processes 53](#_Toc155882946)

[**Supplementary Table S12.** The key materials and energy input intensities of vegetables in the different life cycle processes 54](#_Toc155882947)

[**Supplementary Table S13.** The key materials and energy input intensities of fruits in the different life cycle processes 55](#_Toc155882948)

[**Supplementary Table S14.** The key materials and energy input intensities of animal-sourced foods in the different life cycle processes in Beijing 56](#_Toc155882949)

[**Supplementary Table S15.** The key materials and energy input intensities of animal-sourced foods in the different life cycle processes in Inner Mongolia 57](#_Toc155882950)

[**Supplementary Table S16.** The key materials and energy input intensities of animal-sourced foods in the different life cycle processes in Henan province 58](#_Toc155882951)

[**Supplementary Table S17.** The key materials and energy input intensities of animal-sourced foods in the different life cycle processes in Hebei province 59](#_Toc155882952)

[**Supplementary Table S18.** The key materials and energy input intensities of animal-sourced foods in the different life cycle processes in Shandong province 60](#_Toc155882953)

[**Supplementary Table S19.** Water use of plant-based foods in the different life cycle processes (24) 61](#_Toc155882954)

[**Supplementary Table S20.** The water footprint coefficient of each energy category^j^ 62](#_Toc155882955)

[**Supplementary Table S21.** The energy footprint coefficient of each water category^k^ 63](#_Toc155882956)

[**Supplementary Table S22.** Exergy coefficient of various resources (32,33) 64](#_Toc155882957)

[**Supplementary Table S23.** GHG emission coefficient of food resource in different life cycle processes^l^ 65](#_Toc155882958)

[**Supplementary Table S24.** Prices of various food, energy and water resources in Beijing, 2017^m^ 66](#_Toc155882959)

[**References** 67](#_Toc155882960)

# **1. Details of the integrated optimization model**

# **Supplementary Fig. S1.** Systematic methodological optimization framework for urban system


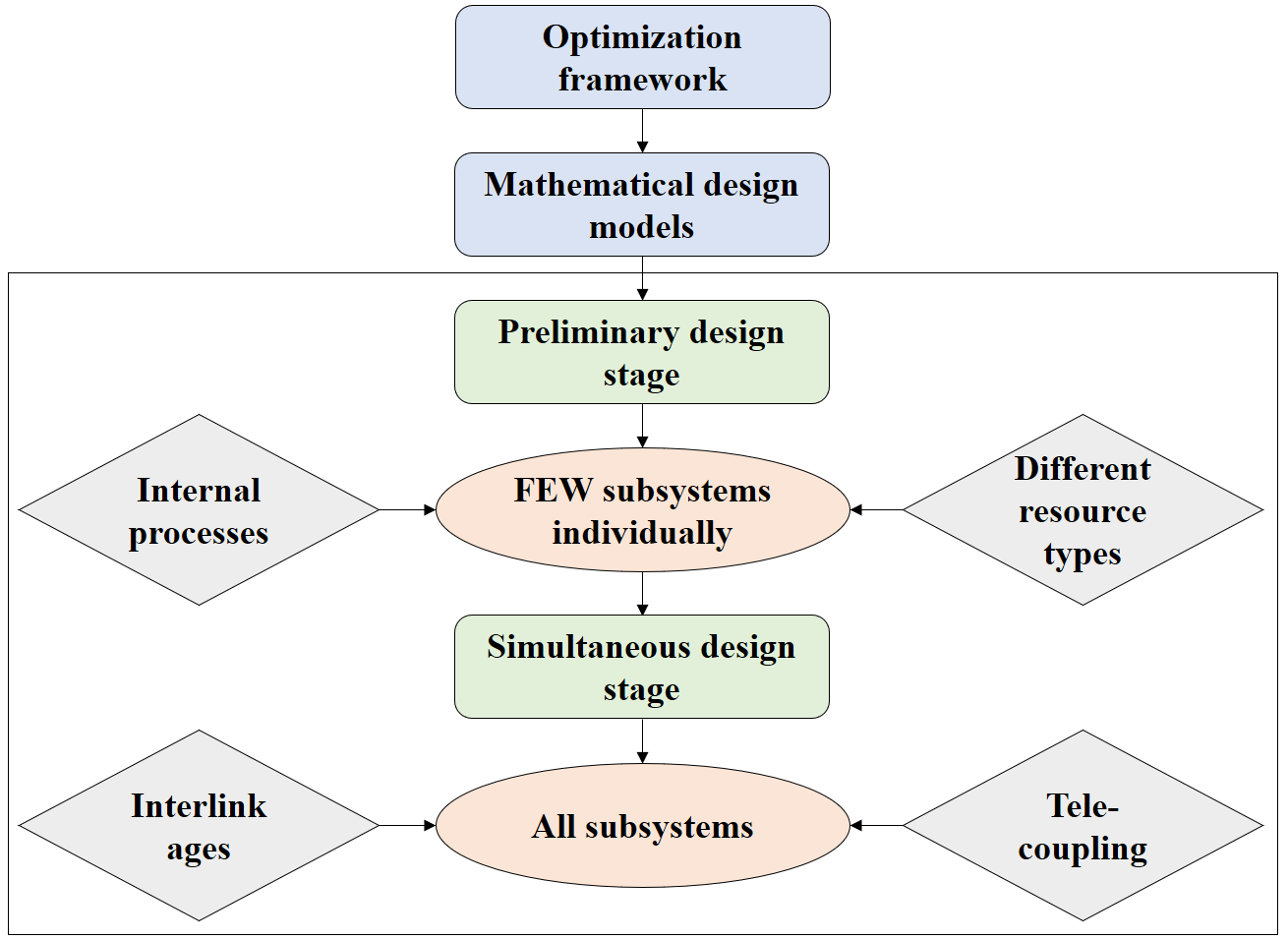


Note: The first step is to build the optimization framework to ensure the different subsystems and the possible internal flow from one resource to another within individual subsystem. The second step is to design mathematical models based on optimization framework. The objective function that minimizes the cumulative exergy consumption for each subsystem is ensured. A set of constraints including the technical measures, the availability of different resources, consumer behaviors, and the interactions among subsystems are also expressed in the mathematical model. The third step is to design the flows of different resource type within individual subsystem, the objective function and decision variables. The fourth step is to combine the individual subsystems as mentioned in the third stop, and identify their interactions, which can formulate an integrated mathematical optimization model. Compared to the ‘silo’ design, this optimization approach can demonstrate the benefits of integrated management on improving resource use efficiency and reducing trade-offs.

# **Supplementary Fig. S2.** Constructing and solving an integrated optimization model


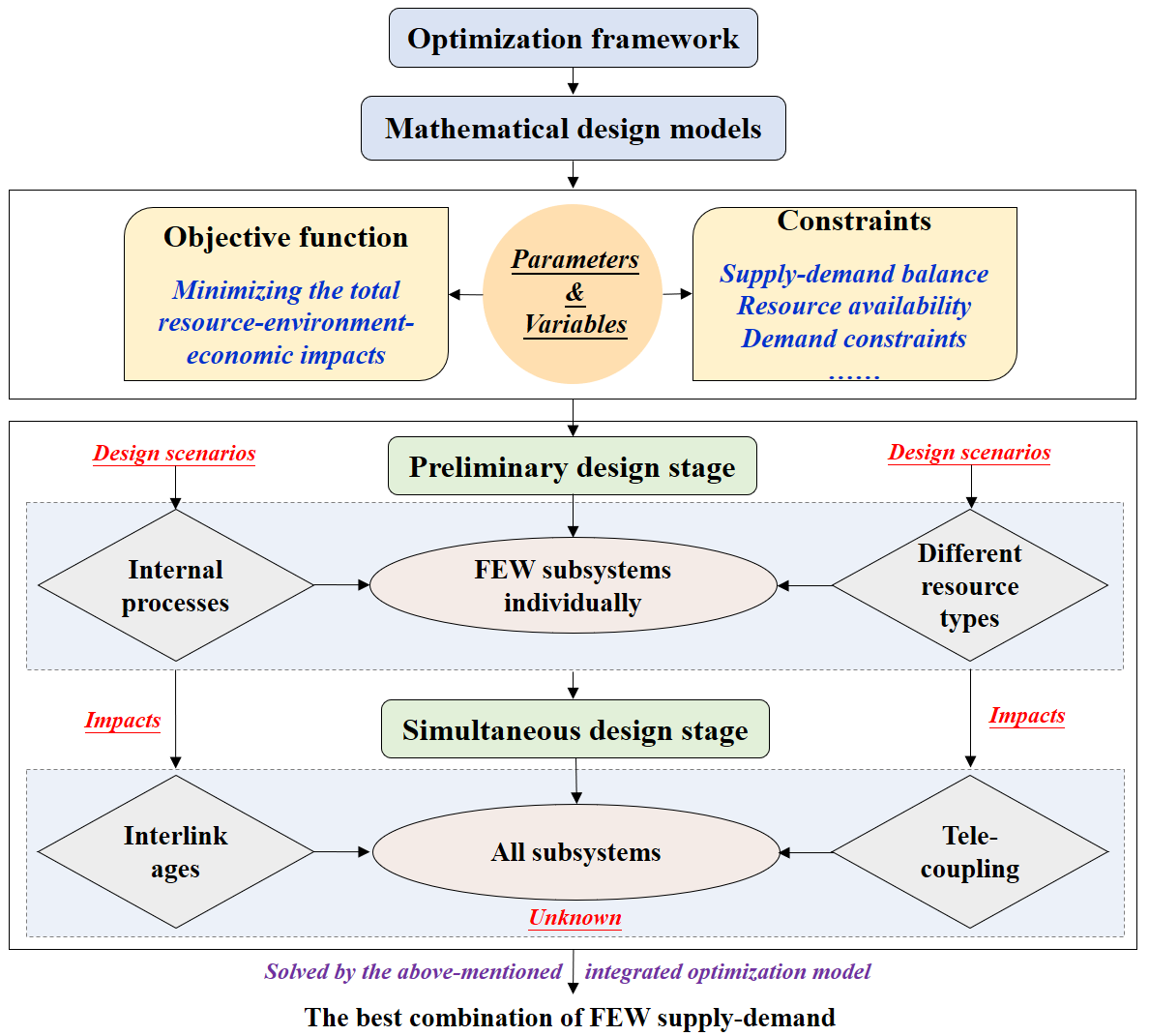


Note: Bases on the systematic methodological optimization framework, illustrated in Fig. S1, the objective function and decision variables are identified. After that, the scenarios are set in view of the changes in internal processes (e.g., technologies and treatment) and resource supply-demand. However, the quantify of supply-demand of each subsystem for socio-economic system and other two subsystems become unknown, due to the interlinkages among FEW subsystems. Therefore, the best combination of FEW supply-demand will be determined via integrated optimization model.

# **Supplementary Fig. S3.** Illustrative the generic superstructure of integrated subsystems


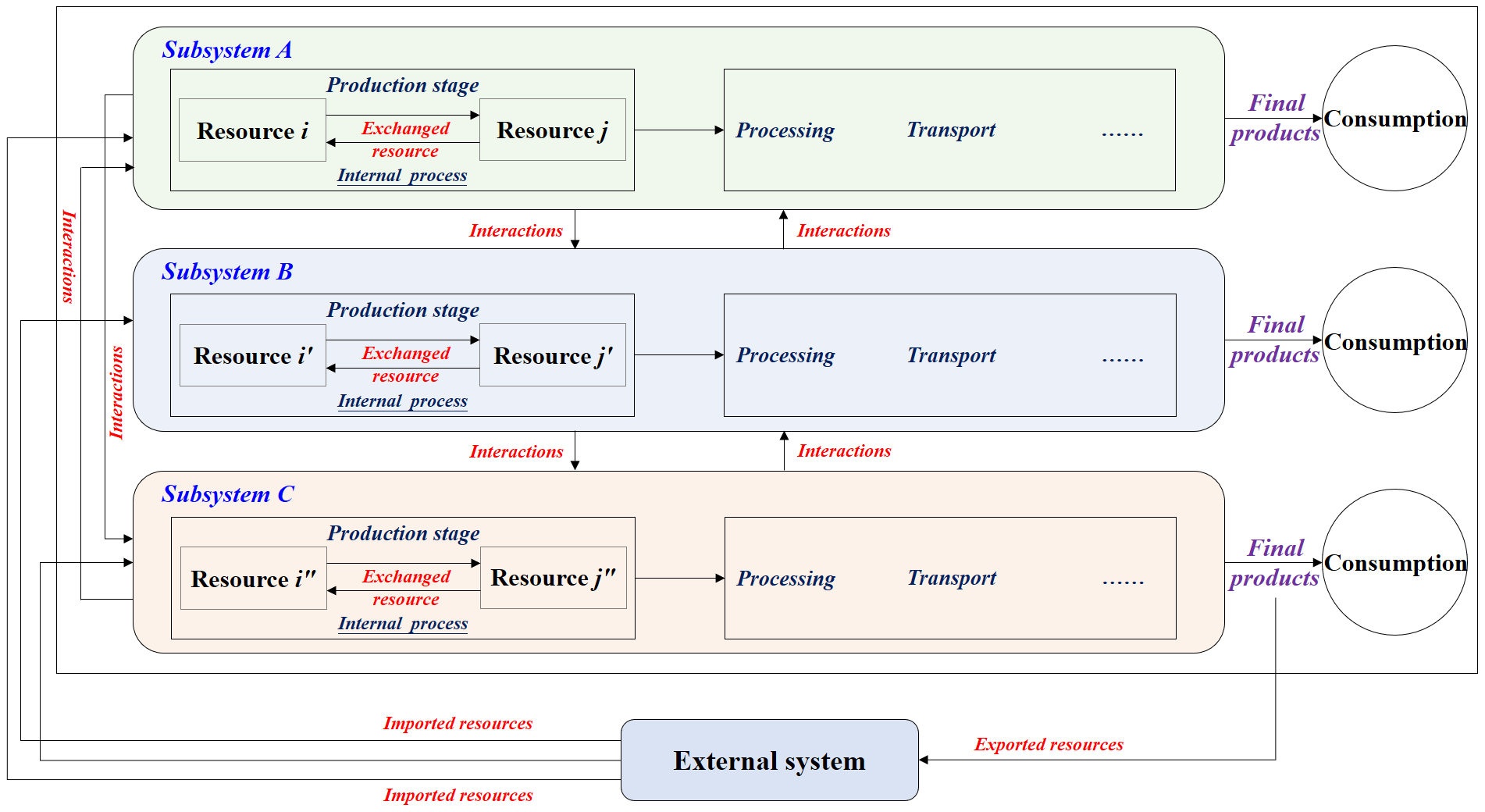


Note: Fig. S3 shows the potential internal relationships within individual subsystem and interactions among different subsystems. It particularly illustrates how exchanged flows within or between the subsystems become the potential interlinkages.

# **Supplementary Fig. S4.** Accounting framework of urban food subsystem in integrated optimization model


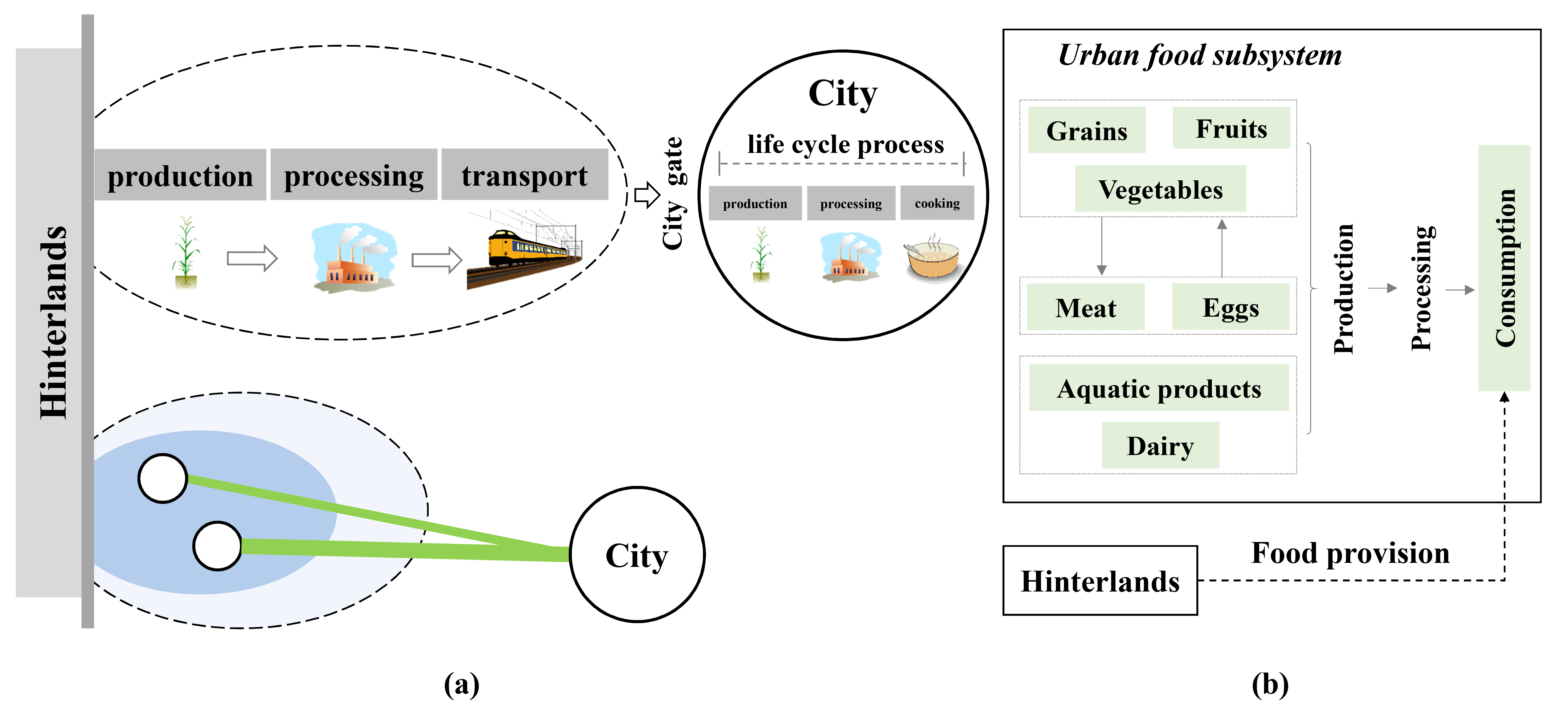


Note: Fig. S4(a) illustrates the system boundary and life cycle process of urban food subsystem (the cradle-to-gate). Considering the openness of urban system, its food sources can be divided into local production and their hinterland supply. In the hinterlands, there were three key life cycle stages, involving food production, processing and transport. Whereas, the life cycle processes in city’s administration boundary included local production, processing and cooking (i.e., local and hinterlands’ food resources). It should be noted that the food loss between harvest and processing and food waste were contained in the production and consumption stages, respectively. The food categories and the interrelationships between grains and meat used in this work are shown in Fig. S4(b). For example, straws from wheat and corn can be used as animal feed, and animal droppings can be used as manure.

# **Supplementary Fig. S5.** Model details for urban food subsystem


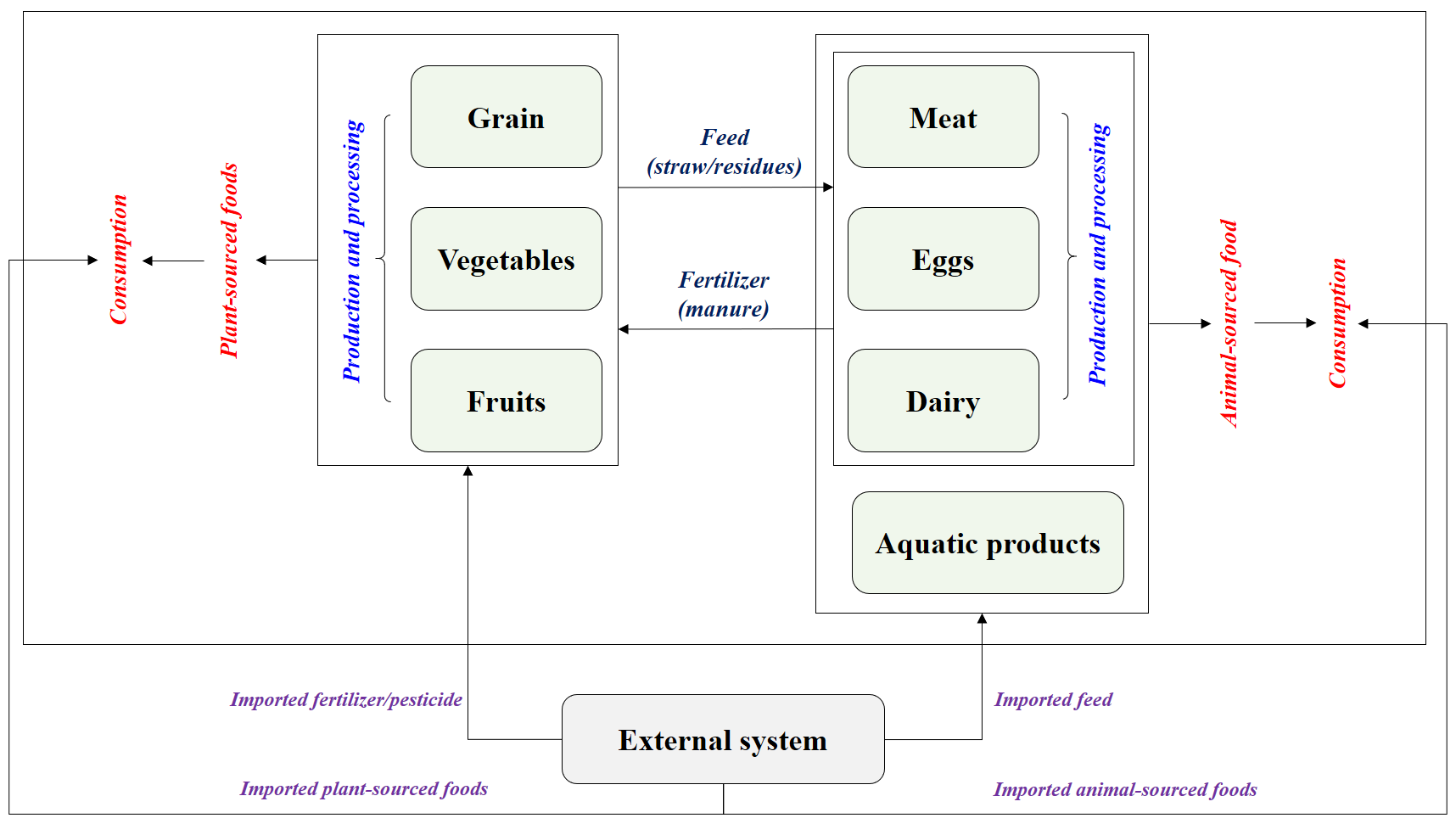


Note: Fig. S5 shows the food types, including plant-sourced and animal-sourced food. The internal processes between plant-sourced and animal-sourced food are also illustrated in Fig. S5.

# **Supplementary Fig. S6.** Accounting framework of urban energy subsystem in integrated optimization model


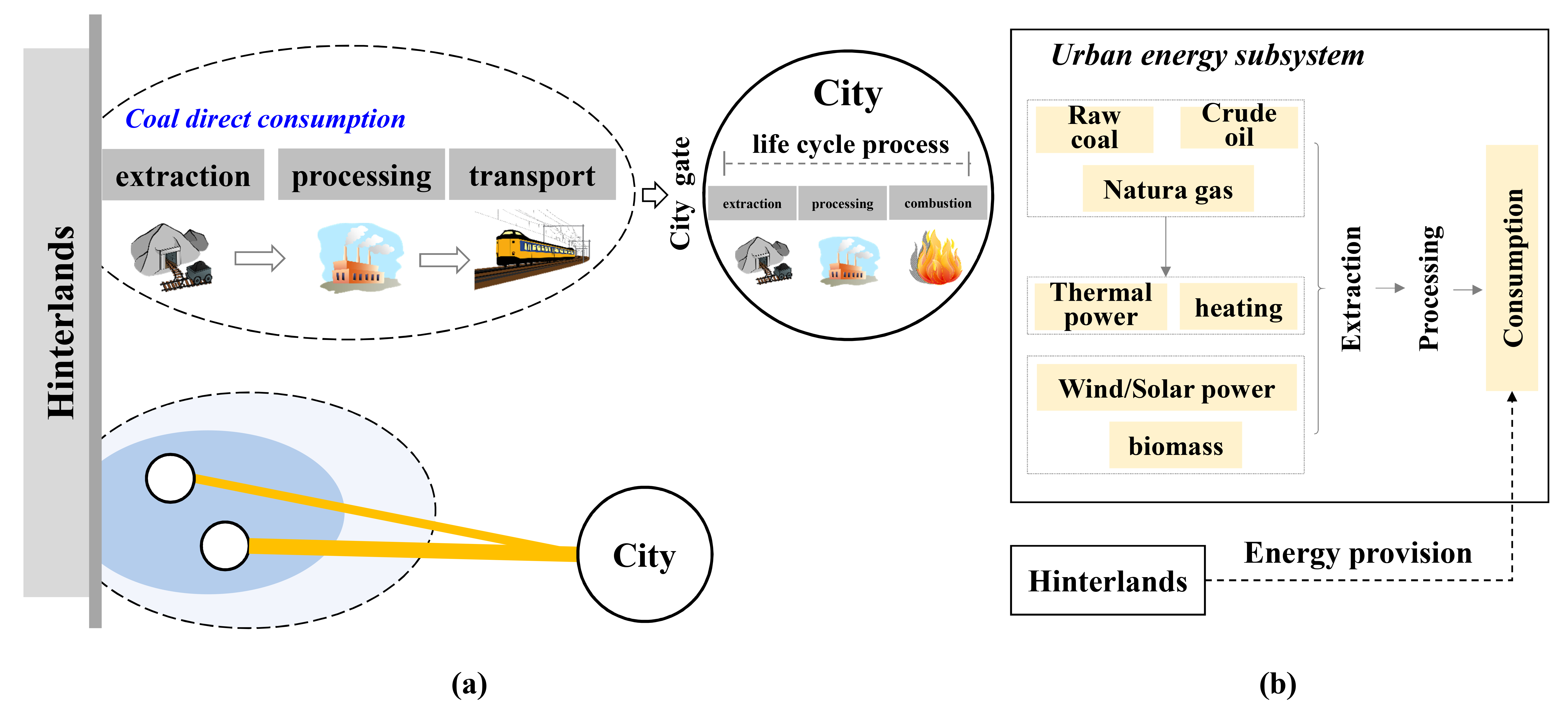


Note: Fig. S6 (a) depicts the system boundary and life cycle process of urban energy subsystem (the well-to-gate). Considering the openness of urban system and resource endowments, there are two types of energy sources, namely local (city) and external (hinterlands) provisions. And they varied in the life cycle processes. Taking direct consumption of raw coal as an example, there were three key life cycle stages in the external system, involving coal extraction, processing and transport. Whereas, the life cycle processes in local system included coal extraction, processing and combustion. It should be noted that the critical life cycle stages of each energy resource were a little difference. As for renewable energy, its life cycle process included three stages, materials and equipment manufacturing, transportation and installation and operation. The functional unit is 1 kWh on-grid electricity provision. Energy categories in this work are shown in Fig. S6 (b), which contains primary fossil energy, renewable energy and secondary energy.

# **Supplementary Fig. S7.** Model details for urban energy subsystem (types and the well-to-gate)


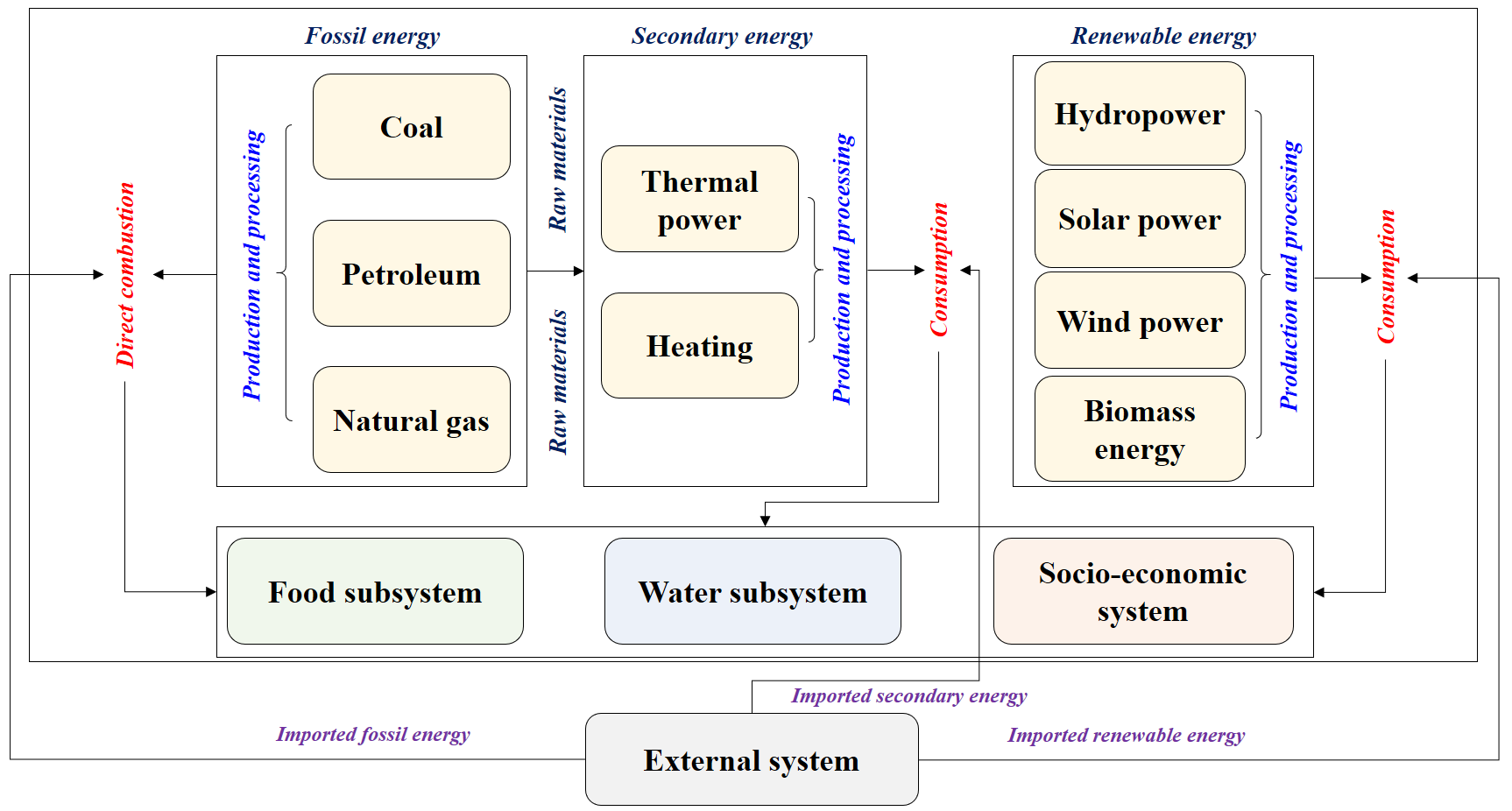


# **Supplementary Fig. S8.** Accounting framework of urban water subsystem in integrated optimization model


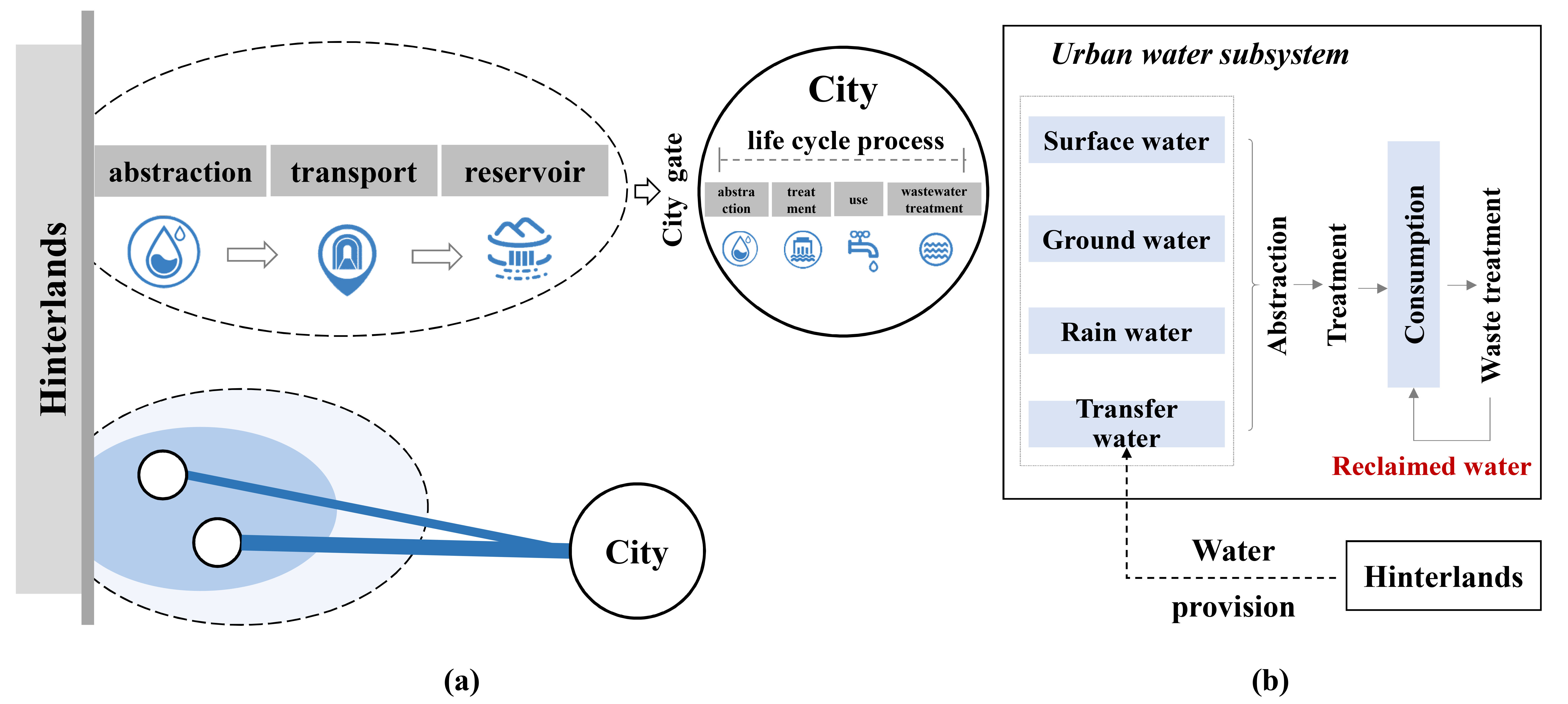


Note: Fig. S8 (a) shows the system boundary and life cycle process of urban water subsystem (the cradle-to-cradle). Similar to patterns of food and energy supply, urban water provision also depends on both local and external systems. However, the largest share of water supply still occurs in local system. As described in Fig. 8 (b), there were three key life cycle stages in the external system, involving water abstraction, transport and reservoir. Whereas, the life cycle processes in local system included water abstraction, transport, reservoir and wastewater treatment. For Beijing city, the South-to-North Water Diversion (SNWD) has been operated to transport water annually from the Danjiangkou Reservoir since Dec 2014 (1). It should be emphasized that the diverted water can simply flow under the influence of gravity with little dependence on pumping stations, due to an approximate 100 meters’ elevation difference between the Danjiangkou Reservoir and Beijing (2). Hence, the water diversion project greatly reduces groundwater exploitation and contribution to regional energy saving (3). As to local system, its life cycle process includes water abstraction, treatment and distribution, end use and wastewater treatment. We selected surface water, groundwater, rainwater, water for the SNWD Project, and reclaimed water (see Fig. S8 (b)).

# **Supplementary Fig. S9.** Model details for urban water subsystem


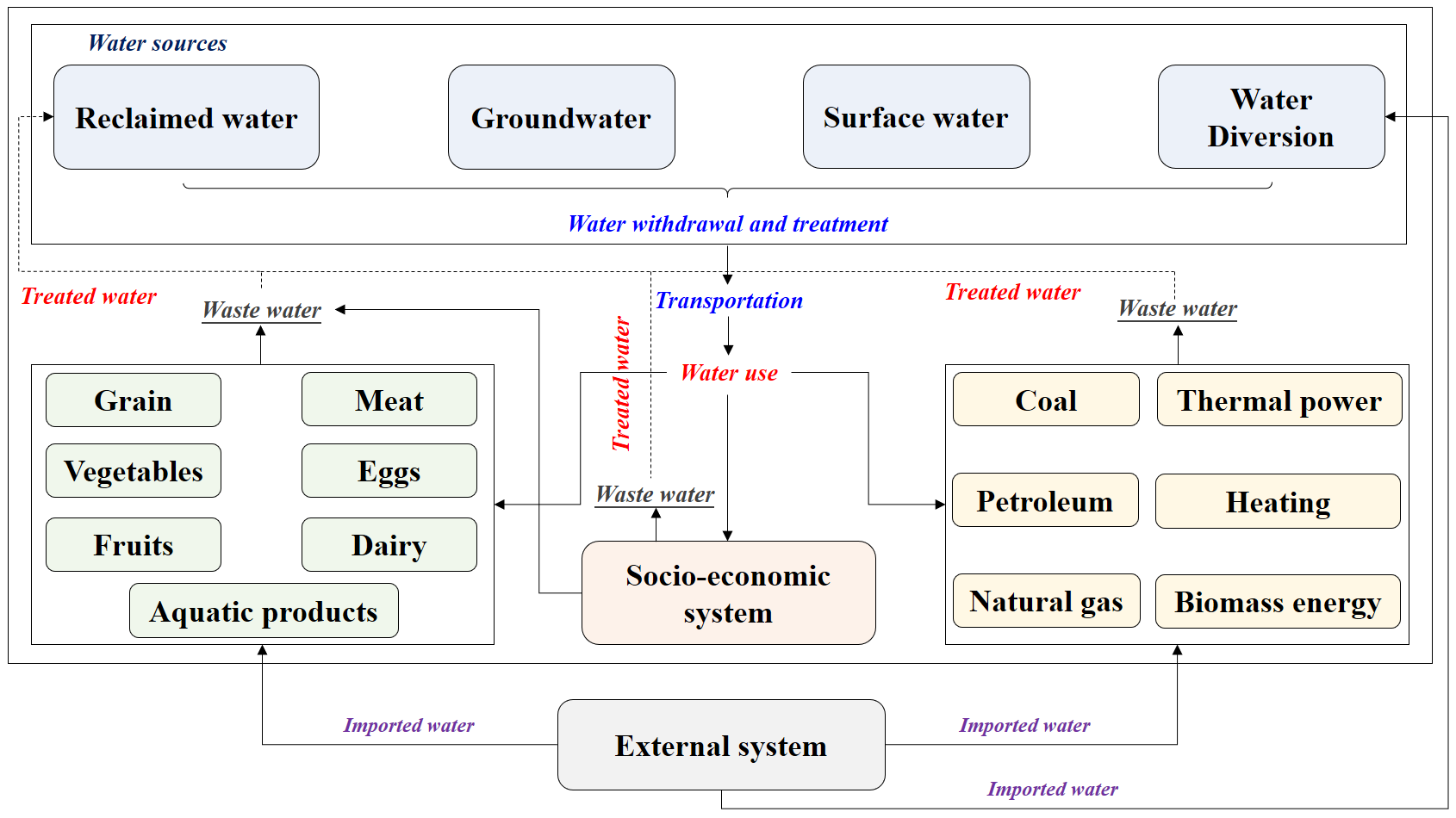


Note: Fig. S9 shows the water sources and the interconnections between water and energy-food subsystems. Besides, the interrelationships between water and socio-economic system are also presented in Fig. S9.

# **Supplementary Fig. S10.** The nexus among FEW and socio-economic system in integrated optimization model


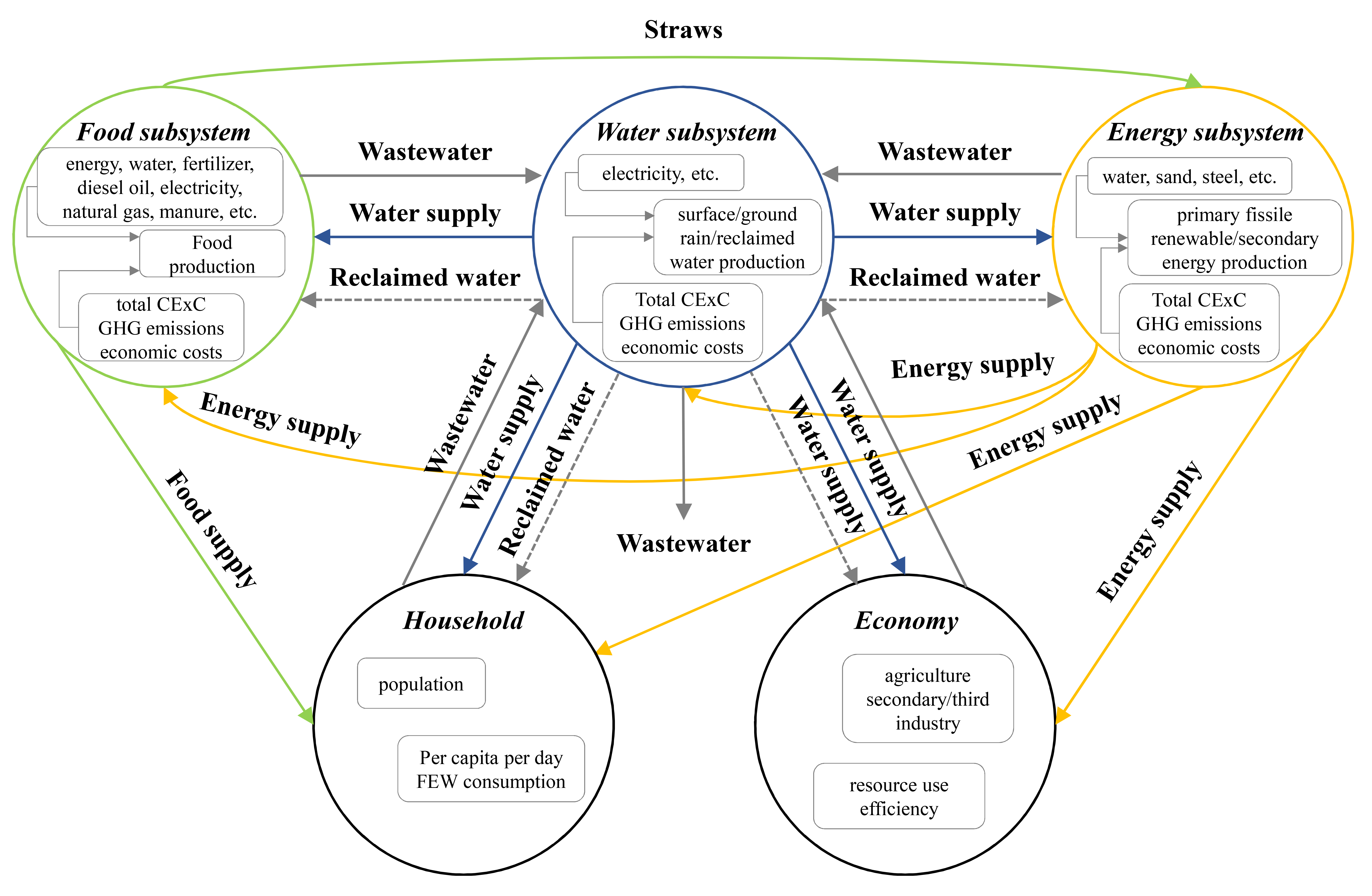


# **Supplementary Fig. S11.** The details of optimization model for urban FEW systems


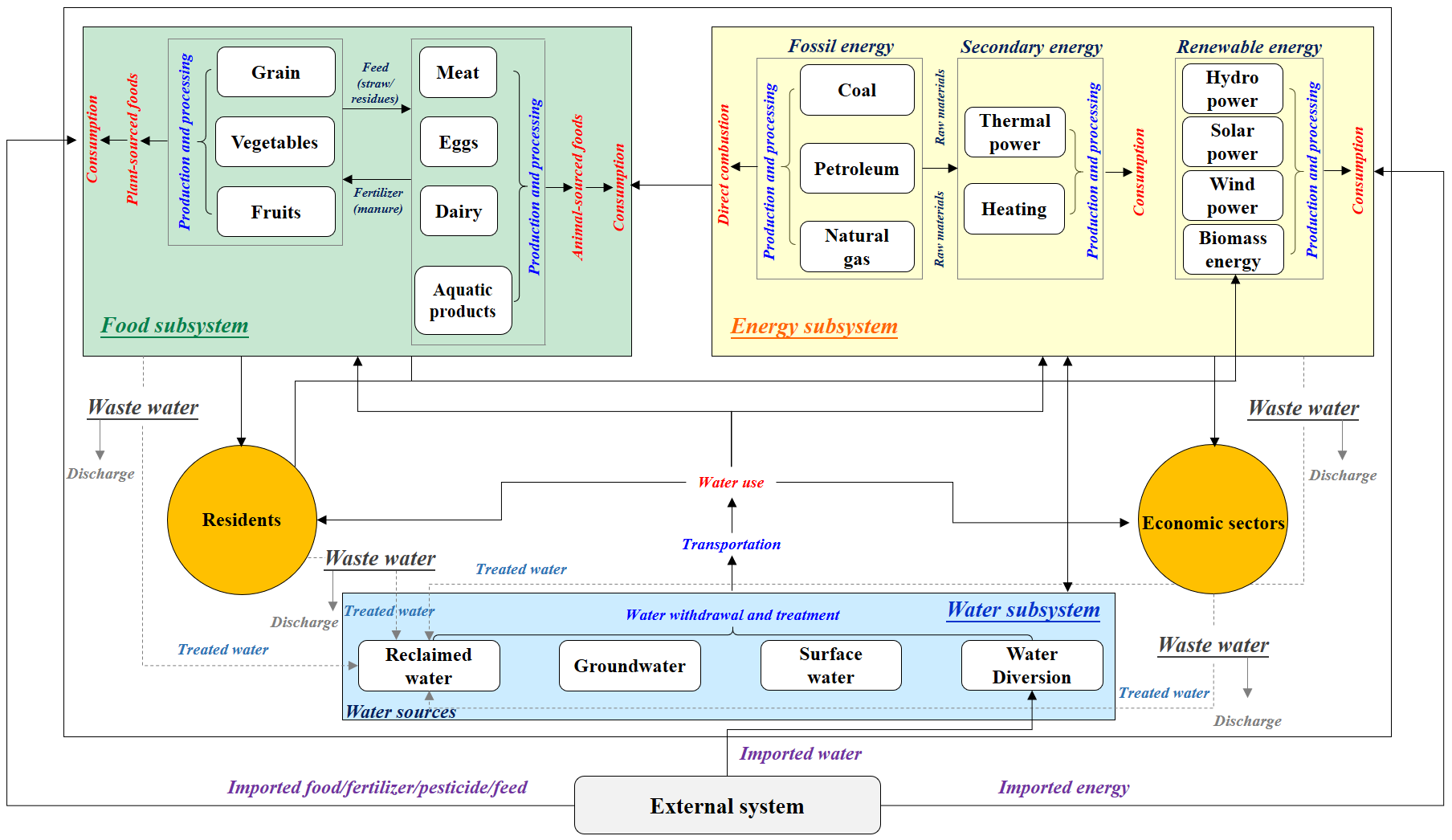


Note: Fig. S11 shows the system boundary (urban and external system—city and its hinterlands), FEW resource types, the life cycle process (e.g., the cradle-to-gate—production, processing, transport, and consumption), the internal process within FEW subsystems and the interconnections among FEW subsystems.

# **2. Constructing scenarios associated with the SDGs**

In this section, we first map the policy groups to the SDGs and define the target of each policy group: Sustainable intensification; Food security and sustainability; Low carbon and cleaner energy; Climate change mitigation; Diversified water supply sources, and Effective and sustainable consumption. A detailed description on of the correspondence between the qualitative targets comprising the seventeen SDGs in their present from and the policies encoding strategies for pursuit of these same targets. Texts in the green, yellow, and blue brackets cite the targets that policy groups are designed to achieve in their respective clusters, respectively.

**2.1 Sustainable intensification**

The targets of the corresponding to the SDGs are shown as follows.

- 2.3 By 2030, double the agricultural productivity and incomes
- 2.4 By 2030, ensure sustainable food production systems and implement resilient agricultural practices that increase productivity and production
- 6.4 By 2030, substantially increase water-use efficiency across all sectors and ensure sustainable withdrawals and supply of freshwater to address water scarcity and substantially reduce the number of people suffering from water scarcity
- 7.3 By 2030, double the global rate of improvement in energy efficiency
- 12.2 By 2030, reduce food losses along production and supply chains, including post-harvest losses

Based on these targets of the SDG 2, 6 7, and 12, we set the key action processes in the policy group “sustainable intensification”—food production subsystem as, Technology improvement; Feed increase; Nutrition cycle; and Source change.

The BAU scenario is the current status of food production (local and external system) in Beijing in 2017.

The changes in key action processes under low-, mid-, and high- levels are shown in Supplementary Table S1.

# **Supplementary Table S1.** The changes in key action processes under -, mid-, and high- levels of urban food production subsystem

| **Food production subsystem**  **Sustainable intensification** | **Key action process** | **Type** | **Low** | **mid** | **high** |
| --- | --- | --- | --- | --- | --- |
|  | Technology improvement (4) | Grain | +1% | +5% | +10% |
|  |  | Vegetable | +1% | +5% | +10% |
|  |  | Fruits | +10% | +15% | +20% |
|  |  | Animal production | +15% | +20% | +25% |
|  | Feed increase (5) | Fertilizer | +1.3% | +1.7% | +2.0% |
|  |  | Feed | +1.5% | +2.0% | +2.5% |
|  | Nutrition cycle | Corn/straw to feed | +5% | +10% | +15% |
|  |  | Manure | +5% | +10% | +15% |
|  | Source change^a^ | Local supply grains/aquatic products | +3% | +5% | +8% |
|  |  | Local supply vegetables/fruits/meat/  eggs/diary | -3% | -5% | -8% |

Note: ^a^Setting the source change was based on the scarcity degree of local food. A scarcity index model was developed to ensure the source change, inspired by the abiotic depletion potential (ADP) coefficient in life cycle analysis (LCA) (6). The formula can be expressed as $SIM\text{=}\frac{Local\sup ply}{(Total\sup ply)^{2}}\times Local\sup ply$ ($SIM$<0.01, increase self-sufficiency rate; $SIM$>0.01, decrease self-sufficiency rate).

# **Supplementary Table S2.** The major food supply regions for Beijing city^b^

| **Food category** | **Major supply regions** |
| --- | --- |
| Grains | Hebei, Henan, Shandong, Heilongjiang, Jilin |
| Vegetables | Hebei, Shandong |
| Fruits | Hebei, Shandong, Guangdong, Hainan |
| Meat | Henan, Shandong, Inner Mongolia |
| Eggs | Henan, Shandong, Hebei |
| Diary | Inner Mongolia |
| Aquatic products | Shandong |

Note: ^b^the major food supply regions were summarized from the previous studies (7), government and agricultural products wholesale market websites (e.g., http://www.xinfadi.com.cn/companyProfile.html)

**2.2 Food security and health**

The targets of the corresponding to the SDGs are shown as follows.

- 2.2 By 2030, end all forms of malnutrition
- 12.1 Implement the 10-year framework of programmes on sustainable consumption and production, all countries taking action, with developed countries taking the lead, taking into account the development and capabilities of developing countries
- 12.2 By 2030, achieve the sustainable management and efficient use of nature resources
- 12.3 By 2030, halve per capita global food waste at the retail and consumer levels and reduce food losses along production and supply chains, including post-harvest losses
- 12.5 By 2030, substantially reduce waste generation through prevention, reduction, recycling and reuse

Based on these targets of the SDG 2 and 12, we set the key action processes in the policy group “dietary change and food security”—food consumption subsystem as, loss reduction; dietary guidelines; waste decrease; and diet alternative.

The BAU scenario is the current status of food consumption in Beijing in 2017.

The changes in key action processes under low-, mid-, and high- levels are shown in Supplementary Table S3.

# **Supplementary Table S3.** The changes in key action processes under -, mid-, and high- levels of urban food consumption subsystem

| **Food consumption subsystem**  **Food security and health** | **Key action process** | **Type** | **Low** | **mid** | **high** |
| --- | --- | --- | --- | --- | --- |
|  | Loss reduction (8) | Grain | +2% | +5% | +8% |
|  |  | Vegetable/Fruits | +8% | +15% | +22% |
|  |  | Livestock products | +4% | +8% | +12% |
|  |  | Aquatic products | +2% | +5% | +10% |
|  | Dietary guidelines^c^ | Intake | Min. | Mid. | Max. |
|  | Waste decrease (8,9) | Retail | -30% | -50% | -70% |
|  |  | When consuming | -30% | -50% | -70% |
|  | Diet alternative^d^ | Reducing meat consumption | 75 | 57.5 | 40 |
|  |  | Reducing eggs consumption | 50 | 45 | 40 |
|  |  | Increasing aquatic products consumption | 40 | 57.5 | 75 |
|  |  | Increasing dairy consumption | 300 | | |

Note: ^c^The dietary guidelines were referred by the Chinese Food Guide Pagoda (2016) in the Chinese Dietary Guidelines, including minimum value, maximum value, and the mean of minimum-maximum values (http://dg.cnsoc.org/). The specific values were described in Supplementary Table S4.

^d^The diet alternative was based on the comparison between current food consumption and the Chinese Food Guide Pagoda (2016) recommended by the Chinese Dietary Guidelines (g/cap ∙ day), and the previous literature (10).

# **Supplementary Table S4.** The Chinese Food Guide Pagoda (2016) recommended by the Chinese Dietary Guidelines (g/cap ∙ day)

| **Food type** | **Minimum value** | **Maximum value** |
| --- | --- | --- |
| Grains | 250 | 400 |
| Vegetables | 300 | 500 |
| Fruits | 200 | 350 |
| Meat | 40 | 75 |
| Eggs | 40 | 50 |
| Dairy | 300 | |
| Aquatic products | 40 | 75 |

**2.3 Low carbon and cleaner energy**

The targets of the corresponding to the SDGs are shown as follows.

- 7.1 By 2030, ensure universal access to affordable, reliable and modern energy services
- 7.2 By 2030, increase substantially the share of renewable energy in the global energy mix
- 7.A By 2030, enhance international cooperation to facilitate access to clean energy research and technology, including renewable energy, energy efficiency and advanced and cleaner fossil-fuel technology, and promote investment in energy infrastructure and clean energy technology
- 12.2 By 2030, achieve the sustainable management and efficient use of natural resources

Based on these targets of the SDG 7 and 12, we set the key action processes in the policy group “low carbon and cleaner energy”—energy production subsystem as, local green power increase; coal to power; coal to natural gas; and imported green power increase from other regions.

The BAU scenario is the current status of energy production (local and external supply) in Beijing in 2017.

The changes in key action processes under low-, mid-, and high- levels are shown in Supplementary Table S5.

# **Supplementary Table S5.** The changes in key action processes under -, mid-, and high- levels of urban energy production subsystem^e^

| **Energy production subsystem**  **Low carbon and cleaner energy** | **Key action process** | **Type** | **Low** | **mid** | **high** |
| --- | --- | --- | --- | --- | --- |
|  | Local renewable energy increase | Wind power | +3 times | +4 times | +5 times |
|  |  | Solar power | +3 times | +4 times | +5 times |
|  |  | Biomass energy | +3 times | +4 times | +5 times |
|  | Coal to power | Direct consumption | +25% | +50% | +100% |
|  | Coal to natural gas | Direct consumption | +25% | +50% | +100% |
|  | Imported green power increase | Power from natural gas | +10% | +15% | +20% |
|  |  | Renewable energy | +10% | +50% | +100% |

Note: ^e^This policy group was mainly based on the Beijing Municipal Commission of Development and Reform (11) and the relative international reports (12,13). These actions also mean the changes in energy technologies.

**2.4 Climate change mitigation**

The targets of the corresponding to the SDGs are shown as follows.

- 7.3 By 2030, Double the global rate of improvement in energy efficiency
- 9.4 By 2030, upgrade infrastructure and retrofit industries to make them sustainable, with increased resource-use efficiency and greater adoption of clean and environmentally sound technologies and industrial processes, with all countries taking action in accordance with their respective capabilities
- 11.6 By 2030, reduce the adverse per capita environmental impact of cities, including by paying special attention to air quality and municipal and other waste management
- 12.1 Implement the 10-year framework of programmes on sustainable consumption and production, all countries taking action, with developed countries taking the lead, taking into account the development and capabilities of developing countries
- 12.2 By 2030, achieve the sustainable management and efficient use of natural resources
- 13.1 Strengthen resilience and adaptive capacity to climate-related hazards and natural disasters in all countries

Based on these targets of the SDG 7, 9, 11, 12 and 13, we set the key action processes in the policy group “climate change mitigation”—energy consumption subsystem as, efficiency improvement; central heating; auto industry transition; and residents saving.

The BAU scenario is the current status of energy consumption in Beijing in 2017.

The changes in key action processes under low-, mid-, and high- levels are shown in Supplementary Table S6.

# **Supplementary Table S6.** The changes in key action processes under -, mid-, and high- levels of urban energy consumption subsystem^f^

| **Energy consumption subsystem**  **Climate change mitigation** | **Key action process** | **Type** | **Low** | **mid** | **high** |
| --- | --- | --- | --- | --- | --- |
|  | Efficiency improvement | 10^4^ yuan/Energy consumption | +3% | +5% | +7% |
|  | Central heating | Direct consumption | +10% | +20% | +30% |
|  | Auto industry transition | Fuel technology | +0.5 | +1.0 | +1.5 |
|  |  | New energy vehicle | +10% | +15% | +20% |
|  | Residents saving | Coal to power | +50% | +80% | +100% |
|  |  | Coal to natural gas | +50% | +80% | +100% |

Note: ^f^This policy group was mainly based on the Beijing Municipal Commission of Development and Reform (11) and the relative international reports (12,13).

**2.5 Diversified water supply**

The targets of the corresponding to the SDGs are shown as follows.

- 6.3 By 2030, improve water quality by reducing pollution, eliminating dumping and minimizing release of hazardous chemicals and materials, halving the proportion of untreated wastewater and substantially increasing recycling and safe reuse globally
- 6.5 By 2030, implement integrated water resources management at all levels, including through transboundary cooperation as appropriate
- 6.A By 2030, expand international cooperation and capacity-building support to developing countries in water- and sanitation-related activities and programmes, including water harvesting, desalination, water efficiency, wastewater treatment, recycling and reuse technologies
- 12.5 By 2030, substantially reduce waste generation through prevention, reduction, recycling and reuse

Based on these targets of the SDG 6 and 12, we set the key action processes in the policy group “diversified water supply sources”—water production subsystem as, waste water reuse; rainwater collection; surface/groundwater saving; and water diversion.

The BAU scenario is the current status of water production (local and external supply) in Beijing in 2017.

The changes in key action processes under low-, mid-, and high- levels are shown in Supplementary Table S7.

# **Supplementary Table S7.** The changes in key action processes under -, mid-, and high- levels of urban water production subsystem^g^

| **Water production subsystem**  **Diversified water supply** | **Key action process** | **Type** | **Low** | **mid** | **high** |
| --- | --- | --- | --- | --- | --- |
|  | Waste water reuse | increasing recycling and safe reuse | +10% | +20% | +30% |
|  | Rainwater collection | Increasing rainwater collection | +60% | +70% | +80% |
|  | Surface/groundwater saving | Decreasing surface water use | -5% | -10% | -15% |
|  |  | Decreasing ground water use | -5% | -10% | -15% |
|  | Water diversion | The South-to-North Water Diversion | +5% | +10% | +15% |

Note: ^g^This policy group was mainly based on the Beijing Water Authority (2016) (14).

**2.6 Effective water use**

The targets of the corresponding to the SDGs are shown as follows.

- 6.1 By 2030, achieve universal and equitable access to safe and affordable drinking water for all
- 6.3 By 2030, improve water quality by reducing pollution, eliminating dumping and minimizing release of hazardous chemicals and materials, halving the proportion of untreated wastewater and substantially increasing recycling and safe reuse globally
- 6.4 By 2030, substantially increase water-use efficiency across all sectors and ensure sustainable withdrawals and supply of freshwater to address water scarcity and substantially reduce the number of people suffering from water scarcity
- 6.5 By 2030, implement integrated water resources management at all levels, including through transboundary cooperation as appropriate
- 6.B Support and strengthen the participation of local communities in improving water and sanitation management
- 11.6 By 2030, reduce the adverse per capita environmental impact of cities, including by paying special attention to air quality and municipal and other waste management
- 12.5 By 2030, substantially reduce waste generation through prevention, reduction, recycling and reuse
- 15.1 By 2020, ensure the conservation, restoration and sustainable use of terrestrial and inland freshwater ecosystems and their services, in particular forests, wetlands, mountains and drylands, in line with obligations under international agreements

Based on these targets of the SDG 6, 11, 12 and 15, we set the key action processes in the policy group “effective and sustainable consumption”—water consumption subsystem as, irrigation; industries; services; and household consumption.

The BAU scenario is the current status of water consumption in Beijing in 2017.

The changes in key action processes under low-, mid-, and high- levels are shown in Supplementary Table S8.

# **Supplementary Table S8.** The changes in key action processes under -, mid-, and high- levels of urban water consumption subsystem^h^

| **Water consumption subsystem**  **Effective water use** | **Key action process** | **Type** | **Low** | **mid** | **high** |
| --- | --- | --- | --- | --- | --- |
|  | Irrigation (5) | Irrigation water use efficiency | 0.75 | 0.8 | 1.0 |
|  | Industries | Decreasing industrial water | -10% | -15% | -20% |
|  | Services | Improving water use efficiency | +20% | +25% | +30% |
|  | Household consumption | Saving water | +5% | +10% | +15% |

Note: ^h^This policy group was mainly based on the Beijing Water Authority (2016) (14) and the previous literature. In the action processes of industries and services, water use efficiency was defined the relationships between value added and water use. In the household consumption, saving water was considered from per capita use.

# **Supplementary Table S9.** The description of scenarios in single policy group

| **Categories** | **Items** | **Contents** | **Categories** | **Items** | **Contents** |
| --- | --- | --- | --- | --- | --- |
| **Food production (F_p_)** | S1 | Technology improvement-FP | **Energy production (E_p_)** | S34 | S27+S30+S31 |
|  | S2 | Feed increase-FP |  | S35 | S28+S30+S31 |
|  | S3 | Nutrition cycle-FP |  | S36 | S29+S30+S31 |
|  | S4 | Source change-FP |  | S37 | S27+S28+S30+S31 |
|  | S5 | S1+S2 |  | S38 | S27+S29+S30+S31 |
|  | S6 | S1+S3 | **Energy consumption (E_c_)** | S39 | Efficiency improvement-EC |
|  | S7 | S1+S4 |  | S40 | Central heating-EC |
|  | S8 | S2+S3 |  | S41 | Auto industry transition (fuel technology)-EC |
|  | S9 | S2+S4 |  | S42 | Auto industry transition (new energy vehicle)-EC |
|  | S10 | S3+S4 |  | S43 | Residents saving (coal to power)-EC |
|  | S11 | S1+S2+S3 |  | S44 | Residents saving (coal to natural gas)-EC |
|  | S12 | S1+S2+S4 |  | S45 | S39+S40 |
|  | S13 | S1+S3+S4 |  | S46 | S39+ S41+S42 |
|  | S14 | S2+S3+S4 |  | S47 | S39+S43 |
|  | S15 | S1+S2+S3+S4 |  | S48 | S39+S44 |
| **Food consumption (F_c_)** | S16 | Loss reduction-FC |  | S49 | S40+ S41+S42 |
|  | S17 | Dietary guideless-FC |  | S50 | S40+S43 |
|  | S18 | Waste decrease-FC |  | S51 | S40+S44 |
|  | S19 | Diet alterative-FC |  | S52 | S41+S42+S43 |
|  | S20 | S16+S17 |  | S53 | S41+S42+S44 |
|  | S21 | S16+S18 |  | S54 | S39+S40+S41+S42 |
|  | S22 | S16+S19 |  | S55 | S39+S40+S43 |
|  | S23 | S17+S18 |  | S56 | S39+S40+S44 |
|  | S24 | S18+S19 |  | S57 | S39+S41+S42+S43 |
|  | S25 | S16+S17+S18 |  | S58 | S39+S41+S42+S44 |
|  | S26 | S16+S18+S19 |  | S59 | S40+S41+S42+S43 |
| **Energy production (E_p_)** | S27 | Local green power increase-EP |  | S60 | S40+S41+S42+S44 |
|  | S28 | Coal to power-EP |  | S61 | S39+S40+S41+S42+S43 |
|  | S29 | Coal to natural gas-EP |  | S62 | S39+S40+S41+S42+S44 |
|  | S30 | Thermal power-natural gas-EP | **Water production (W_p_)** | S63 | Waste water reuse-WP |
|  | S31 | Imported green power increase-EP |  | S64 | Rainwater collection-WP |
|  | S32 | S27+S28 |  | S65 | Surface water saving-WP |
|  | S33 | S27+S29 |  | S66 | Groundwater saving-WP |
| **Water production (W_p_)** | S67 | Water diversion-WP | **Water consumption (W_c_)** | S80 | Industries-WC |
|  | S68 | S63+S64 |  | S81 | Services-WC |
|  | S69 | S63+S65+S66 |  | S82 | Household consumption-WC |
|  | S70 | S63+S67 |  | S83 | S79+S80 |
|  | S71 | S64+S65+S66 |  | S84 | S79+S81 |
|  | S72 | S64+S67 |  | S85 | S79+S82 |
|  | S73 | S65+S66+S67 |  | S86 | S80+S81 |
|  | S74 | S63+S64+S65+S66 |  | S87 | S80+S82 |
|  |  |  |  | S88 | S81+S82 |
|  | S75 | S63+S64+S67 |  | S89 | S79+S80+ S81 |
|  | S76 | S63+S65+S66+S67 |  | S90 | S79+S80+ S82 |
|  | S77 | S64+S65+S66+S67 |  | S91 | S79+S81+S82 |
|  | S78 | S63+ S64+S65+S66+S67 |  | S92 | S80+ S81+ S82 |
|  | S79 | Irrigation-WC |  | S93 | S79+S80+S81+S82 |

Note: the amount of scenarios in single policy group were 93×3 levels (279 scenarios).

# **Supplementary Table S10.** The description of scenarios in integrated policy groups

| **Categories** | **Items** | **Contents** | **Categories** | **Items** | **Contents** |
| --- | --- | --- | --- | --- | --- |
| **F_p_** + **F_c_** | S94 | S15+S25 | **F_p_** + **F_c_** + **W_c_** | S135 | S15+S25+S93 |
|  | S95 | S15+S26 |  | S136 | S15+S26+S93 |
| **F_p_** + **E_p_** | S96 | S15+S37 | **F_p_** + **E_p_** + **E_c_** | S137 | S15+S37+S61 |
|  | S97 | S15+S38 |  | S138 | S15+S38+S62 |
| **F_p_** + **E_c_** | S98 | S15+S61 | **F_p_** + **E_p_** + **W_p_** | S139 | S15+S37+S78 |
|  | S99 | S15+S62 |  | S140 | S15+S38+S78 |
| **F_p_** + **W_p_** | S100 | S15+S78 | **F_p_** + **E_p_** + **W_c_** | S141 | S15+S37+S93 |
| **F_p_** + **W_c_** | S101 | S15+S93 |  | S142 | S15+S38+S93 |
| **F_c_** + **E_p_** | S102 | S25+S37 | **F_p_** + **E_c_** + **W_p_** | S143 | S15+S61+S78 |
|  | S103 | S25+S38 |  | S144 | S15+S62+S78 |
|  | S104 | S26+S37 | **F_p_** + **E_c_** + **W_c_** | S145 | S15+S61+S93 |
|  | S105 | S26+S38 |  | S146 | S15+S62+S93 |
| **F_c_ + E_c_** | S106 | S25+S61 | **F_p_** + **W_p_**+ **W_c_** | S147 | S15+S78+S93 |
|  | S107 | S25+S62 | **F_c_** + **E_p_** + **E_c_** | S148 | S25+S37+S61 |
|  | S108 | S26+S61 |  | S149 | S25+S38+S62 |
|  | S109 | S26+S62 |  | S150 | S26+S37+S61 |
| **F_c_** + **W_p_** | S110 | S25+S78 |  | S151 | S26+S38+S62 |
|  | S111 | S26+S78 | **F_c_** + **E_p_** + **W_p_** | S152 | S25+S37+S78 |
| **F_c_** + **W_c_** | S112 | S25+S93 |  | S153 | S25+S38+S78 |
|  | S113 | S26+S93 |  | S154 | S26+S37+S78 |
| **E_p_** + **E_c_** | S114 | S37+S61 |  | S155 | S26+S38+S78 |
|  | S115 | S38+S62 | **F_c_** + **E_p_** + **W_c_** | S156 | S25+S37+S93 |
| **E_p_** + **W_p_** | S116 | S37+S78 |  | S157 | S25+S38+S93 |
|  | S117 | S38+S78 |  | S158 | S26+S37+S93 |
| **E_p_** + **W_c_** | S118 | S37+S93 |  | S159 | S26+S38+S93 |
|  | S119 | S38+S93 | **F_c_** + **E_c_** + **W_p_** | S160 | S25+S61+S78 |
| **E_c_** + **W_p_** | S120 | S61+S78 |  | S161 | S25+S62+S78 |
|  | S121 | S62+S78 |  | S162 | S26+S61+S78 |
| **E_c_** + **W_c_** | S122 | S61+S93 |  | S163 | S26+S62+S78 |
|  | S123 | S62+S93 | **F_c_** + **E_c_** + **W_c_** | S164 | S25+S61+S93 |
| **W_p_** + **W_c_** | S124 | S78+S93 |  | S165 | S25+S62+S93 |
| **F_p_** + **F_c_**+ **E_p_** | S125 | S15+S25+S37 |  | S166 | S26+S61+S93 |
|  | S126 | S15+S25+S38 |  | S167 | S26+S62+S93 |
|  | S127 | S15+S26+S37 | **F_c_** +**W_p_** + **W_c_** | S168 | S25+S78+S93 |
|  | S128 | S15+S26+S38 |  | S169 | S26+S78+S93 |
| **F_p_** + **F_c_**+ **E_c_** | S129 | S15+S25+S61 | **E_p_** + **E_c_** + **W_p_** | S170 | S37+S61+S78 |
|  | S130 | S15+S25+S62 |  | S171 | S38+S62+S78 |
|  | S131 | S15+S26+S61 | **E_p_** + **E_c_** + **W_c_** | S172 | S37+S61+S93 |
|  | S132 | S15+S26+S62 |  | S173 | S38+S62+S93 |
| **F_p_** + **F_c_**+ **W_p_** | S133 | S15+S25+S78 | **E_p_** +**W_p_** + **W_c_** | S174 | S37+S78+S93 |
|  | S134 | S15+S26+S78 |  | S175 | S38+S78+S93 |
| **E_c_** +**W_p_** + **W_c_** | S176 | S61+S78+S93 | **F_c_**+**E_p_**+**E_c_**+**W_c_** | S214 | S26+S37+S61+S93 |
|  | S177 | S62+S78+S93 |  | S215 | S26+S38+S62+S93 |
| **F_p_**+**F_c_**+**E_p_**+**E_c_** | S178 | S15+S25+S37+S61 | **F_c_**+**E_p_**+**W_p_**+**W_c_** | S216 | S25+S37+S78+S93 |
|  | S179 | S15+S25+S38+S62 |  | S217 | S25+S38+S78+S93 |
|  | S180 | S15+S26+S37+S61 |  | S218 | S26+S37+S78+S93 |
|  | S181 | S15+S26+S38+S62 |  | S219 | S26+S38+S78+S93 |
| **F_p_**+**F_c_**+**E_p_**+**W_p_** | S182 | S15+S25+S37+S78 | **F_c_**+**E_c_**+**W_p_**+**W_c_** | S220 | S25+S61+S78+S93 |
|  | S183 | S15+S25+S38+S78 |  | S221 | S25+S62+S78+S93 |
|  | S184 | S15+S26+S37+S78 |  | S222 | S26+S61+S78+S93 |
|  | S185 | S15+S26+S38+S78 |  | S223 | S26+S62+S78+S93 |
| **F_p_**+**F_c_**+**E_p_**+**W_c_** | S186 | S15+S25+S37+S93 | **E_p_**+**E_c_**+**W_p_**+**W_c_** | S224 | S37+S61+S78+S93 |
|  | S187 | S15+S25+S38+S93 |  | S225 | S38+S62+S78+S93 |
|  | S188 | S15+S26+S37+S93 | **F_p_**+**F_c_**+  **E_p_**+**E_c_**+**W_p_** | S226 | S15+S25+S37+S61+S78 |
|  | S189 | S15+S26+S38+S93 |  | S227 | S15+S25+S38+S62+S78 |
| **F_p_**+**F_c_**+**E_c_**+**W_p_** | S190 | S15+S25+S61+S78 |  | S228 | S15+S26+S37+S61+S78 |
|  | S191 | S15+S25+S62+S78 |  | S229 | S15+S26+S38+S62+S78 |
|  | S192 | S15+S26+S61+S78 | **F_p_**+**F_c_**+  **E_p_**+**E_c_**+**W_c_** | S230 | S15+S25+S37+S61+S93 |
|  | S193 | S15+S26+S62+S78 |  | S231 | S15+S25+S38+S62+S93 |
| **F_p_**+**F_c_** +**E_c_**+**W_c_** | S194 | S15+S25+S61+S93 |  | S232 | S15+S26+S37+S61+S93 |
|  | S195 | S15+S25+S62+S93 |  | S233 | S15+S26+S38+S62+S93 |
|  | S196 | S15+S26+S61+S93 | **F_p_**+**F_c_**+  **E_p_**+**W_p_**+**W_c_** | S234 | S15+S25+S37+S78+S93 |
|  | S197 | S15+S26+S62+S93 |  | S235 | S15+S25+S38+S78+S93 |
| **F_p_**+**F_c_**+**W_p_**+**W_c_** | S198 | S15+S25+S78+S93 |  | S236 | S15+S26+S37+S78+S93 |
|  | S199 | S15+S26+S78+S93 |  | S237 | S15+S26+S38+S78+S93 |
| **F_p_**+**E_p_**+**E_c_**+**W_p_** | S200 | S15+S37+S61+S78 | **F_p_**+**F_c_**+  **E_c_**+**W_p_**+**W_c_** | S238 | S15+S25+S61+S78+S93 |
|  | S201 | S15+S38+S62+S78 |  | S239 | S15+S25+S62+S78+S93 |
| **F_p_**+**E_p_**+**E_c_**+**W_c_** | S202 | S15+S37+S61+S93 |  | S240 | S15+S26+S61+S78+S93 |
|  | S203 | S15+S38+S62+S93 |  | S241 | S15+S26+S62+S78+S93 |
| **F_p_**+**E_p_**+**W_p_**+**W_c_** | S204 | S15+S37+S78+S93 | **F_p_**+**E_p_**+  **E_c_**+**W_p_**+**W_c_** | S242 | S15+S37+S61+S78+S93 |
|  | S205 | S15+S38+S78+S93 |  | S243 | S15+S38+S62+S78+S93 |
| **F_p_**+**E_c_**+**W_p_**+**W_c_** | S206 | S15+S61+S78+S93 | **F_c_**+**E_p_**+  **E_c_**+**W_p_**+**W_c_** | S244 | S25+S37+S61+S78+S93 |
|  | S207 | S15+S62+S78+S93 |  | S245 | S25+S38+S62+S78+S93 |
| **F_c_**+**E_p_**+**E_c_**+**W_p_** | S208 | S25+S37+S61+S78 |  | S246 | S26+S37+S61+S78+S93 |
|  | S209 | S25+S38+S62+S78 |  | S247 | S26+S38+S62+S78+S93 |
|  | S210 | S26+S37+S61+S78 | **F_p_**+**F_c_**+**E_p_**+  **E_c_**+**W_p_**+**W_c_** | S248 | S15+S25+S37+S61+S78+S93 |
|  | S211 | S26+S38+S62+S78 |  | S249 | S15+S25+S38+S62+S78+S93 |
| **F_c_**+**E_p_**+**E_c_**+**W_c_** | S212 | S25+S37+S61+S93 |  | S250 | S15+S26+ S37+S61+S78+S93 |
|  | S213 | S25+S38+S62+S93 |  | S251 | S15+S26+S38+S62+S78+S93 |

Note: the amount of scenarios in the integrated policy groups were 158×3 levels (474 scenarios).

# **3. Results to support the main conclusion**

# **Supplementary Fig. S12.** The total cumulative exergy consumption (CExC) and the FEW nexus of baseline scenario (BAU) in Beijing, 2017


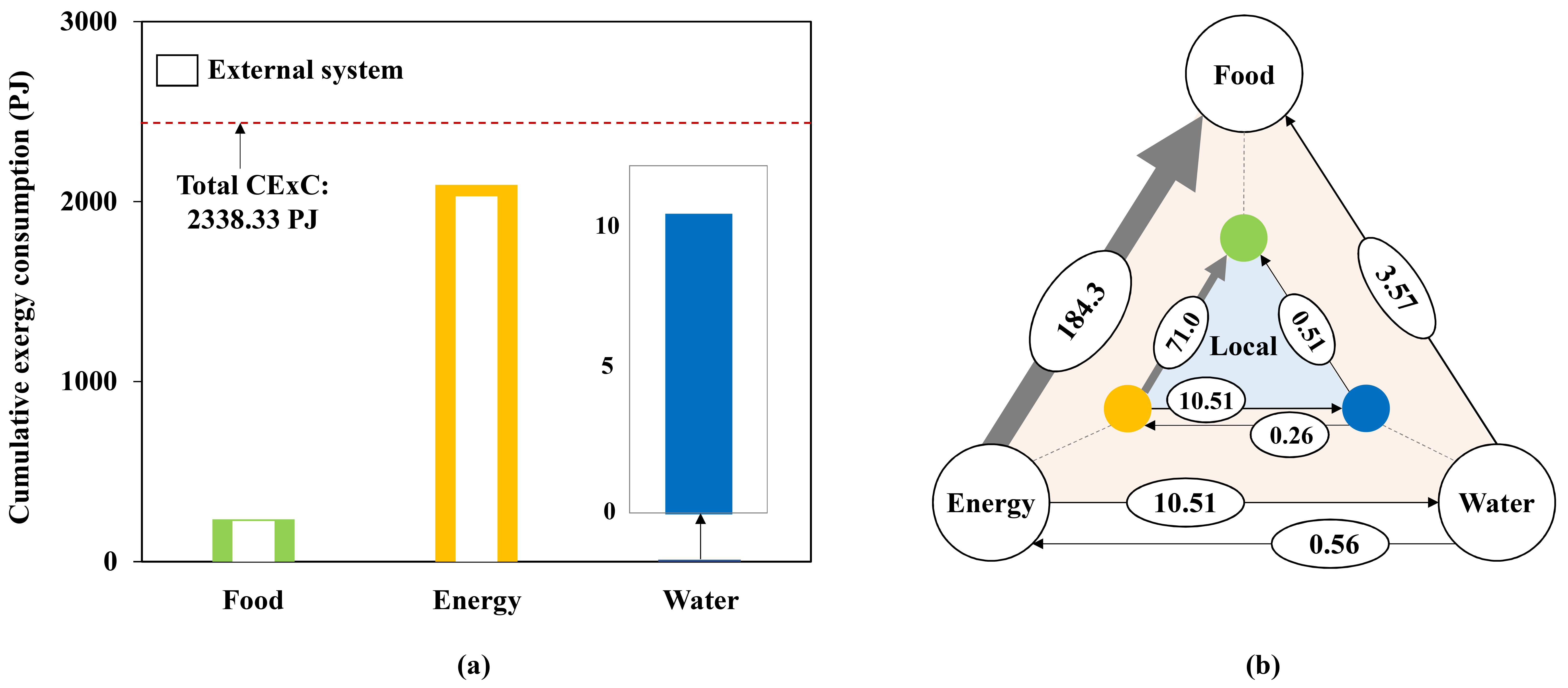


Note: Fig. S12 (a) show the total cumulative exergy consumption (CExC) in Beijing (2017) was 2338.33 PJ. And among them, energy subsystem contributed over 90% of the total CExC. In addition, except water subsystem, most of the CExC occurred outside Beijing’s administration boundary (i.e. external system). Fig. S12 (b) shows the interconnection among FEW systems. We used the amount of exergy consumption to express the FEW nexus. The arrow represents the flow direction of exergy consumption. For example, the 184.3 PJ of exergy consumption means energy consumption by food subsystem.

# **Supplementary Fig. S13.** The per capita food consumption and water use from different sources in the BAU scenario, compared with the recommend standards and the upper limit of water utilization


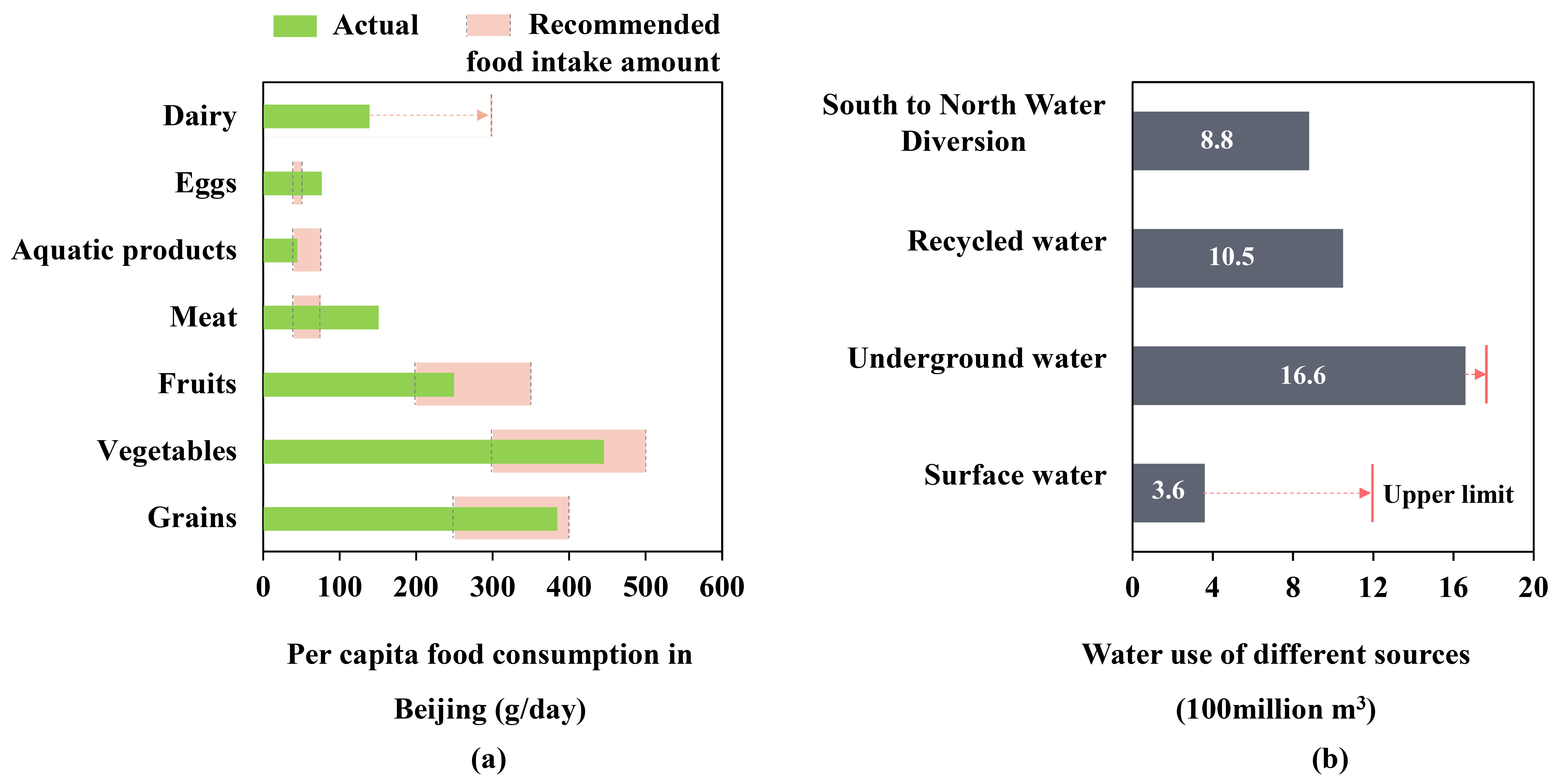


# **Supplementary Fig. S14.** The total CExC and economic cost of different integrated policy groups


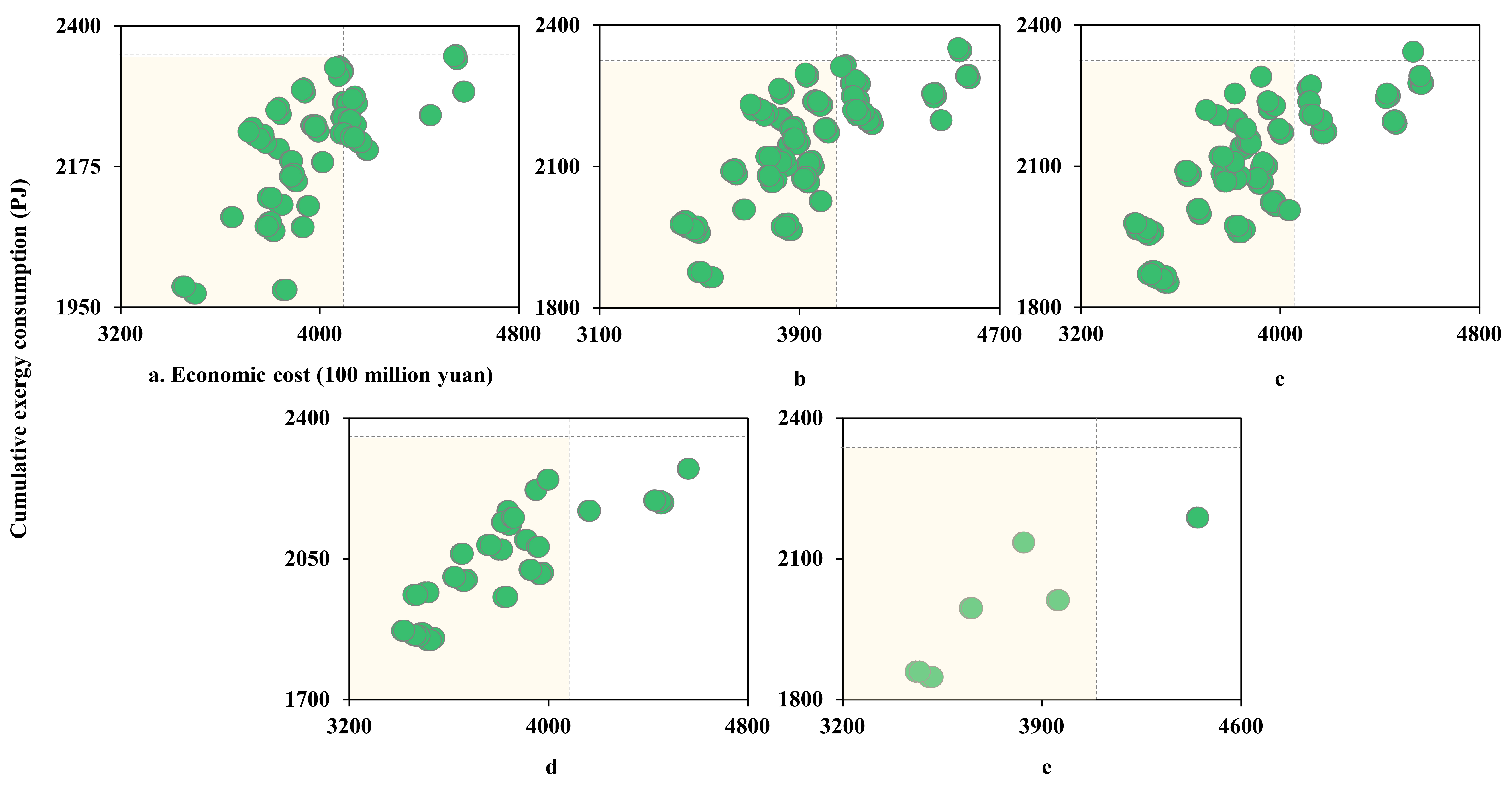


Note: Fig. S14 a, b, c, d and e show the optimization results of different scenarios in two, three, four, five and six policy groups, respectively.

# **Supplementary Fig. S15.** GHG emissions and economic cost of different integrated policy groups


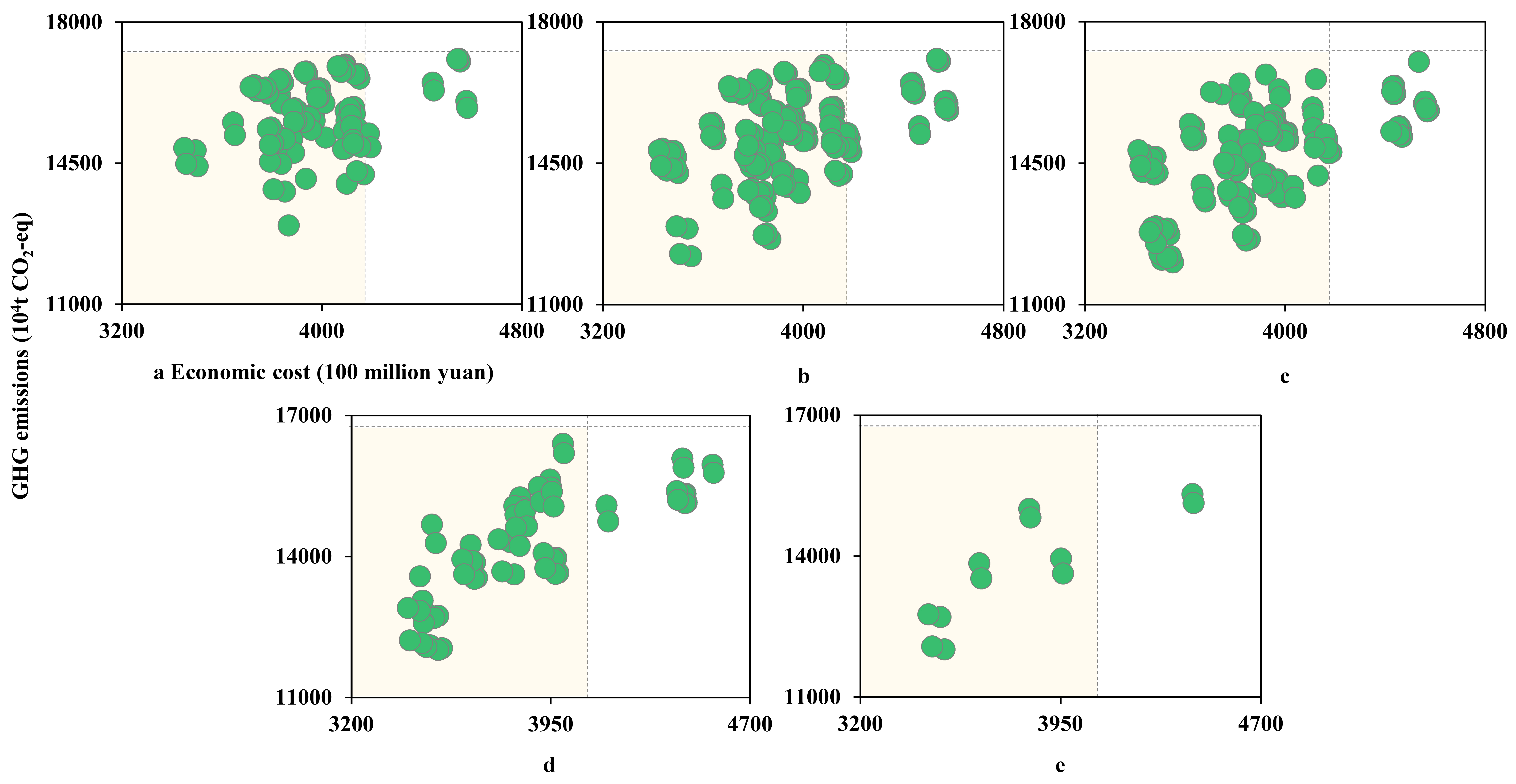


Note: Fig. S15 a, b, c, d and e show the optimization results of different scenarios in two, three, four, five and six policy groups, respectively.

# **Supplementary Fig. S16.** The total cumulative exergy consumption (CExC) and economic cost of six single policy groups


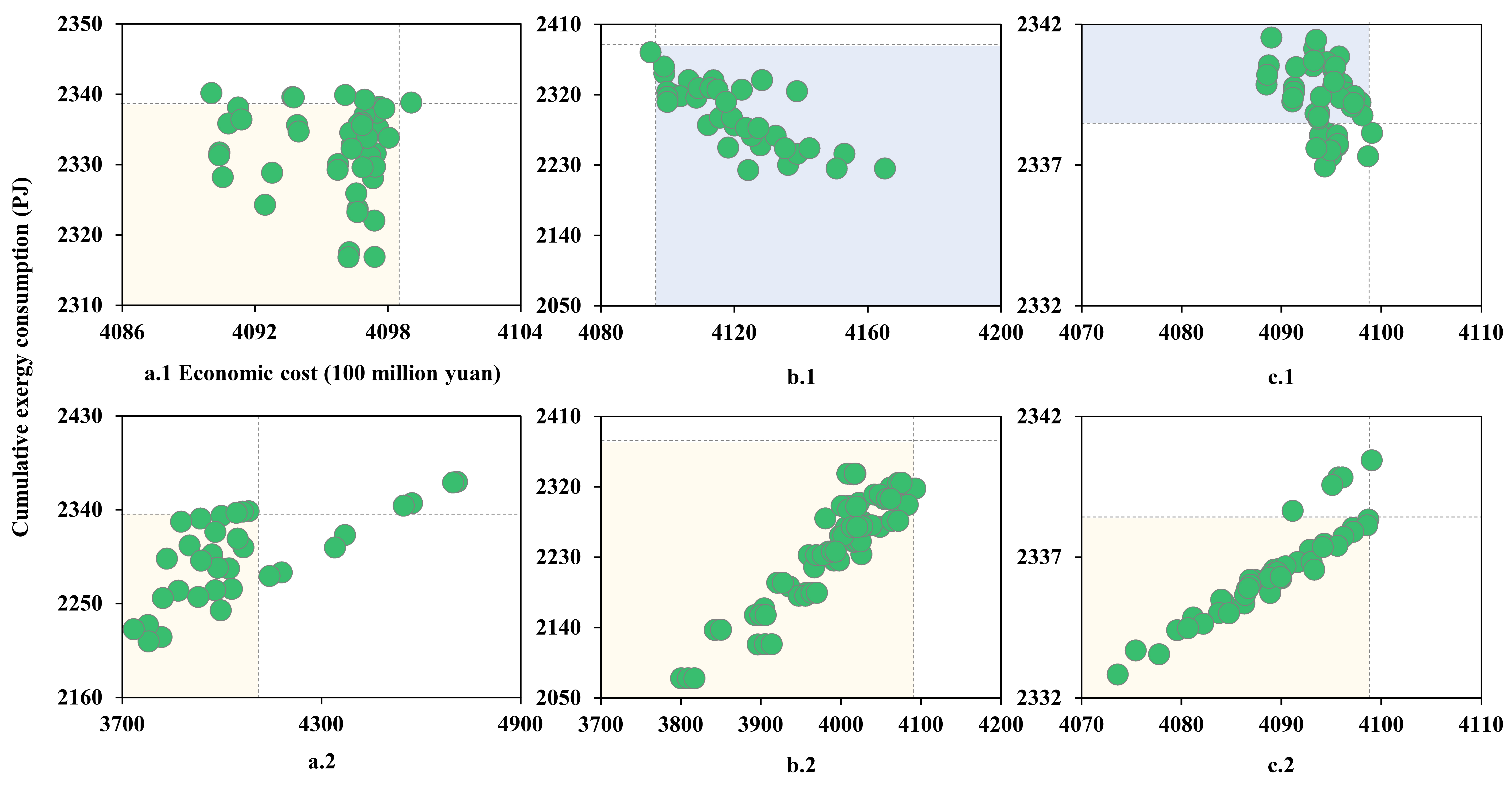


Note: Fig. S16 a.1 and a.2 show the optimization results of the total CExC and economic cost of each scenario in the food production and consumption policy group, respectively. Fig. S16 b.1 and b.2 show the optimization results of the total CExC and economic cost of each scenario in the energy production and consumption policy group, respectively. Fig. S16 c.1 and c.2 show the optimization results of the total CExC and economic cost of each scenario in the water production and consumption policy group, respectively.

# **Supplementary Fig. S17.** GHG emissions and economic cost of six single policy groups


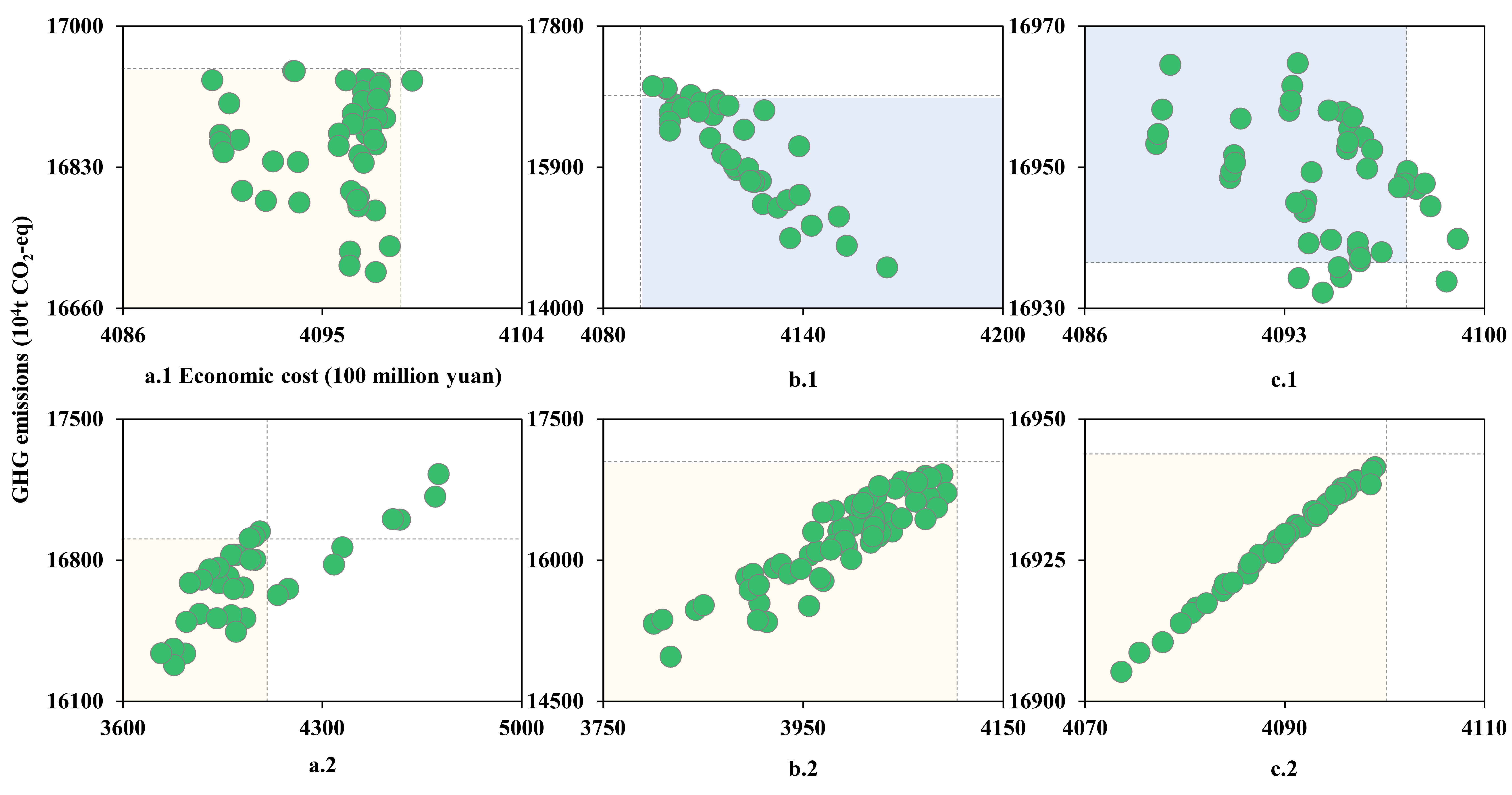


Note: Fig. S17 a.1 and a.2 show the optimization results of GHG emissions and economic cost of each scenario in the food production and consumption policy group, respectively. Fig. S17 b.1 and b.2 show the optimization results of GHG emissions and economic cost of each scenario in the energy production and consumption policy group, respectively. Fig. S17 c.1 and c.2 show the optimization results of GHG emissions and economic cost of each scenario in the water production and consumption policy group, respectively.

# **4. The uncertainty and sensitivity analysis of our results**

The data used in the study was primarily obtained from official releases, published literature, authoritative international institutions, and the Ecoinvent database (see Data sources in the Materials and Methods). In addition, we also compared the GHG emissions and economic cost of the baseline scenario with statistical data. The result was found to be reasonable (see Fig. S18 in the Supplementary Materials).

Supplementary Fig. S10 and S11 depict the multiple sources of uncertainty, encompassing different resources, materials inputs, GHG emissions and economic cost. Specifically, the accounting of food subsystem focused on four stages throughout its life cycle: production (including tillage, sowing, irrigation, fertilization, pesticide use, and harvest), processing (such as rain threshing and slaughter), transport and consumption (cooking). It should be noting that previous studies have already addressed the verse sources of uncertainty in each subsystem, and our unit result for resource-material inputs and GHG emissions generally align with the ranges reported in the reviews (15-17).

Considering the complex and numerous parameters in our integrated optimization model, we mitigated uncertainty in our results by conducting multiple scenarios. Specifically, we analyzed 251 individual scenarios across 3 different levels, totaling 753 scenarios. This modeling not only demonstrates the robustness of our model but also emphasizes the reliability of our results. Additionally, we also performed a sensitivity analysis to investigate the effects of changes in FEW production and consumption assumptions at low, medium and high levels. Our results, complementing the research of Van Vuuren et al. (2019) (18), reveal that the energy subsystem is highly responsive to cumulative exergy consumption and GHG emissions. For instance, assuming constant production and consumption in the food and water subsystems, implementing changes in the energy subsystem (i.e., low carbon and cleaner energy, and climate change mitigation) to reduce GHG emissions would lead to a 6.1%, 6.3%, and 6.7% greater reduction compared to changes in the food subsystem assumption at low, medium, and high levels, respectively. Furthermore, our findings indicate that alterations in food consumption can affect economic cost. When keeping production and consumption in the energy and water subsystems unchanged, modifying food consumption to reduce economic cost would result in a 1.8%, 2.6% and 2.9% higher reduction compared to changes in the energy subsystem assumption at low, medium, and high levels, respectively.

# **Supplementary Fig. S18.** The GHG emissions and economic cots of baseline scenario (BAU) in Beijing, 2017


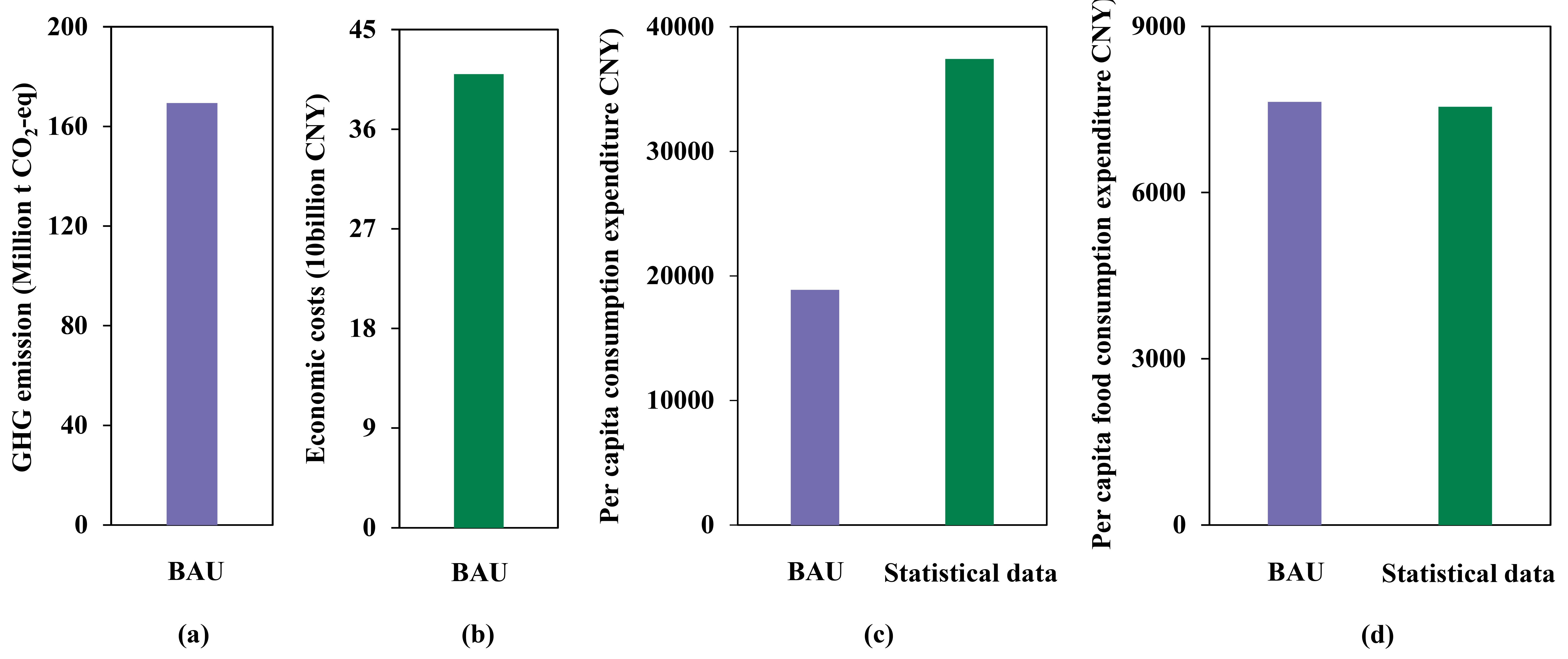


Note: The per capita consumption expenditure in BAU was lower than that taken from the statistical data. This result was reasonable. The per capita consumption expenditure in BAU only contained FEW consumption, while that in statistical data still included clothing, live, education and entertainment, medical treatment and other services.

# **Supplementary Fig. S19.** Uncertainty analysis of the optimization results (the total CExC) associated with single and integrated policy groups at low, mid and high levels


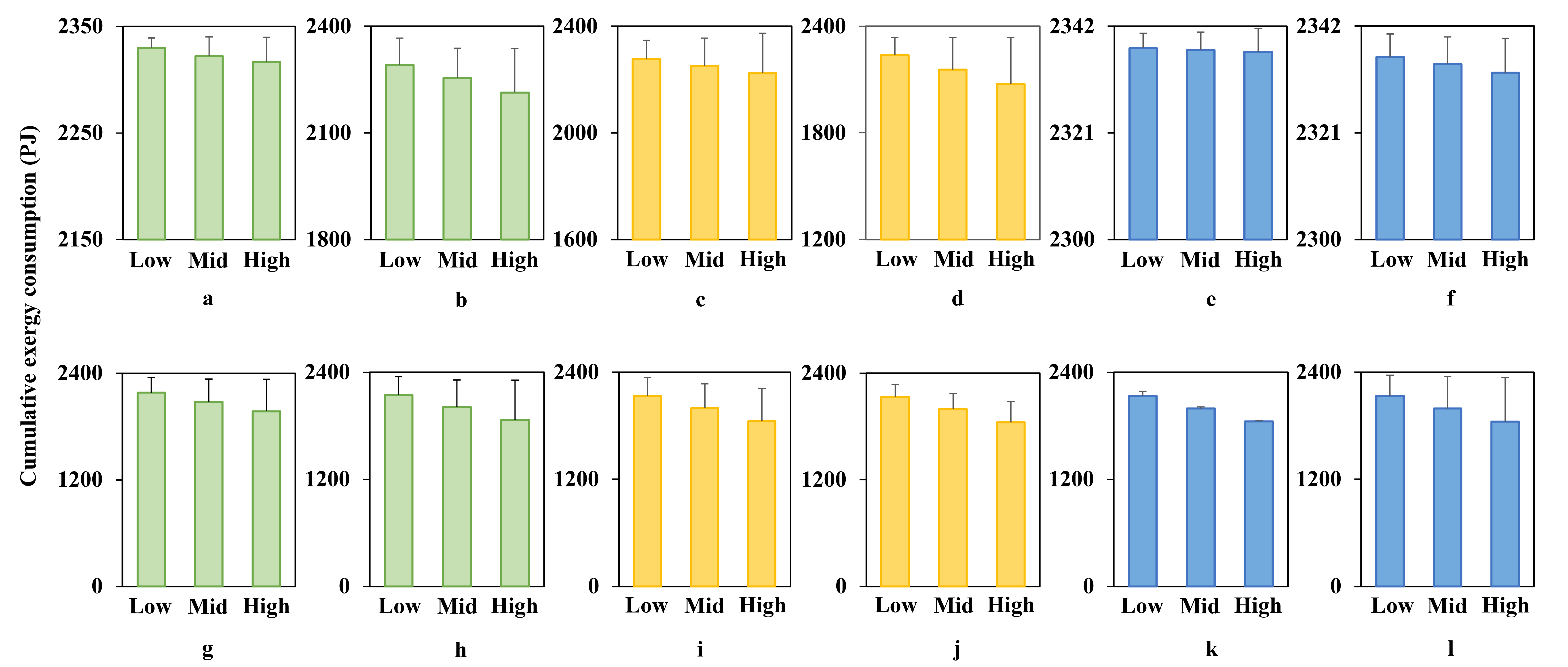


Note: Fig. S19 a, b, c, d, e, and f show the uncertainty analysis of the optimization results (the total CExC) associate with food production, food consumption, energy production, energy consumption, water production and water consumption policy group, respectively. Fig. S19 g, h, i, j, and k show the uncertainty analysis of the optimization results (the total CExC) associate with two, three, four, five and six integrated policy groups, respectively. Fig. S19 l show the uncertainty analysis of the optimization results (the total CExC) associate with the whole scenarios (753 scenarios).

# **Supplementary Fig. S20.** Uncertainty analysis of the optimization results (GHG emissions) associated with single and integrated policy groups


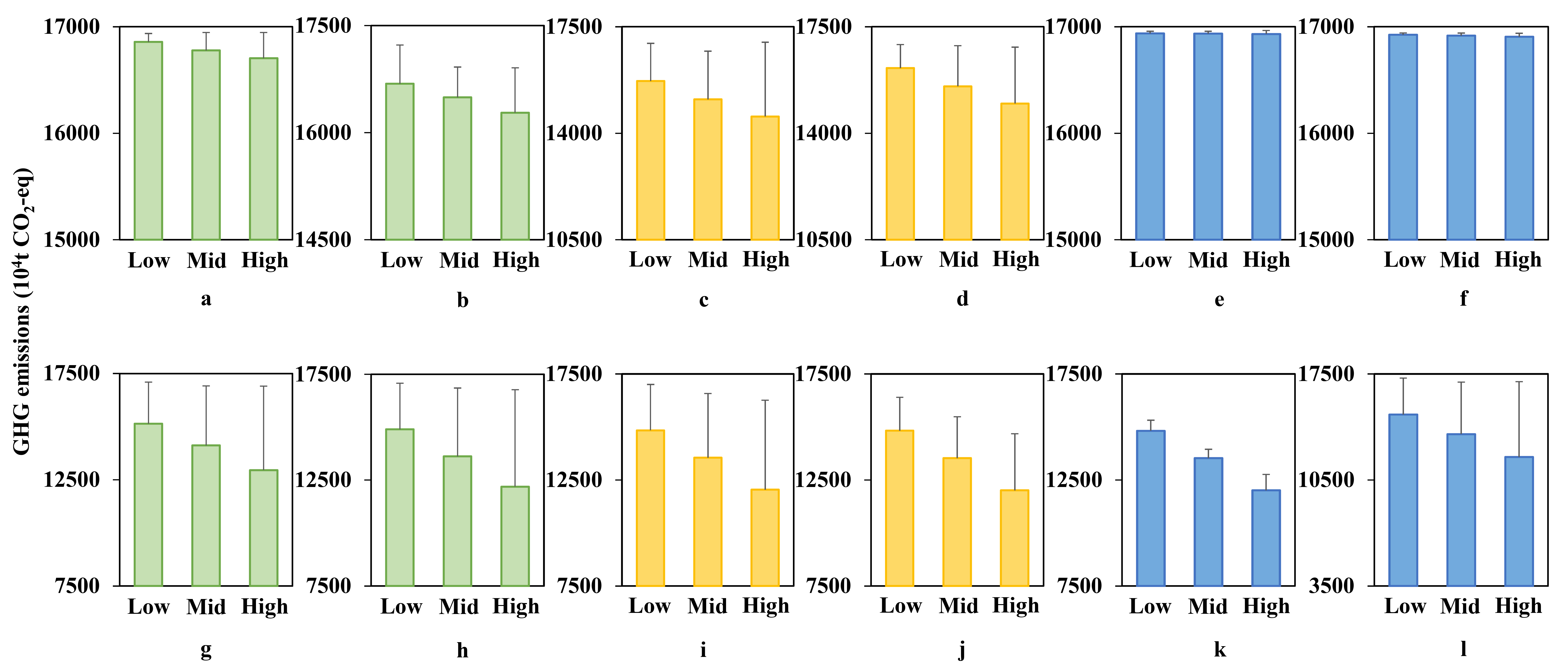


Note: Fig. S20 a, b, c, d, e, and f show the uncertainty analysis of the optimization results (GHG emissions) associate with food production, food consumption, energy production, energy consumption, water production and water consumption policy group, respectively. Fig. S20 g, h, i, j, and k show the uncertainty analysis of the optimization results (GHG emissions) associate with two, three, four, five and six integrated policy groups, respectively. Fig. S20 l show the uncertainty analysis of the optimization results (GHG emissions) associate with the whole scenarios (753 scenarios).

# **Supplementary Fig. S21.** Uncertainty analysis of the optimization results (economic cost) associated with single and integrated policy groups


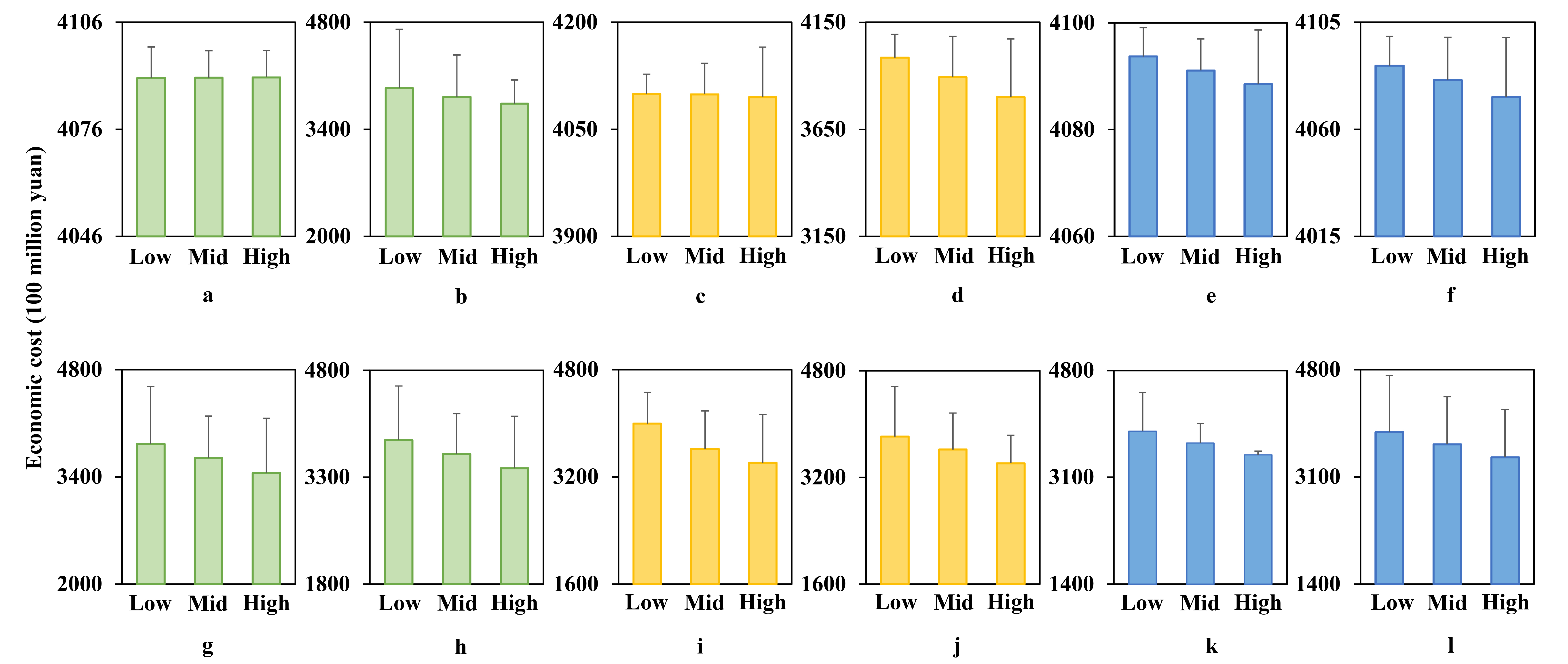


Note: Fig. S21 a, b, c, d, e, and f show the uncertainty analysis of the optimization results (economic cost) associate with food production, food consumption, energy production, energy consumption, water production and water consumption policy group, respectively. Fig. S21 g, h, i, j, and k show the uncertainty analysis of the optimization results (economic cost) associate with two, three, four, five and six integrated policy groups, respectively. Fig. S21 l show the uncertainty analysis of the optimization results (economic cost) associate with the whole scenarios (753 scenarios).

# **5. The explanation of key parameters and variables**

| **Nomenclature**  **Sets** $i\in I$ food type $c\in i$ Crop products $l\in L$ food from local system $im\in IM$ imported from external system $j\in J$ energy type $r\in R$ Energy raw material $k\in K$ water type  **Parameters** $DRI_{i}^{min}$ the minimum food intakes based on the Chines Dietary Guidelines, g/day $DRI_{i}^{max}$ the maximum food intakes based on the Chines Dietary Guidelines, g/day $p$ the amount of permanent resident population in urban area, 10^4^ $L_{c,current}^{local}$ the current land use of crop $c$, ha $ELD_{current}^{coal-el}$ the current electricity supply from coal fired, 10^8^ kWh $ELD_{current}^{ng-el}$ the current electricity supply from natural fired, 10^8^ kWh $ELD_{current}^{renew-el}$ the current electricity supply from renewable energy, 10^8^ kWh $M_{renew-el}^{installed}$ the installed capacity of renewable energy, MW $\lambda_{c}$ the straw amount per unit yield of crop $c$ $A_{c}^{local}$ the demand of crop $c$, t $H_{c}$ the collection and utilization rate of crop straw $\eta_{r}^{el}$ the electricity conservation from raw material $r$ $W_{gr}^{current}$ the current use of groundwater, 10^8^ m^3^ $W_{sur}^{current}$ the current use of surface water, 10^8^ m^3^ $W_{ra}^{planning}$ the availability of rainwater in urban planning, 10^8^ m^3^ $W_{diversion}^{planning}$ the availability of water diversion in urban planning, 10^8^ m^3^ $Ref_{cod}$ the COD removal efficiency of the treatment plant, % $ex_{i}$ the exergy coefficient of food$i$, PJ/Mt $ex^{fertilizer}$ the exergy coefficient of fertilizer, PJ/Mt $ex^{pesticide}$ the exergy coefficient of pesticide, PJ/Mt $ex^{plastic}$ the exergy coefficient of plastic, PJ/Mt $ex^{feed}$ the exergy coefficient of feed, PJ/Mt $ex_{j}$ the exergy coefficient of energy$j$, PJ/Mt, PJ/10^8^m^3^, PJ/10^8^kWh $ELD_{food,i}^{production}$ the use of electricity in food production process, kWh/kg $Oil_{food,i}^{production}$ the use of oil in food production process, kg/kg $Coal_{food,i}^{production}$ the use of coal in food production process, kg/kg $\xi_{k}^{eld}$ the electricity use coefficient of different water types, kWh/m^3^ $\beta_{j,per}^{urban-household}$ the energy demand coefficient of per capita urban household, t/per, m^3^/per, kWh/per $\beta_{j,per}^{rural-household}$ the energy demand coefficient of per capita rural household, t/per, m^3^/per, kWh/per  **Variables** $f_{i}^{dem}$ the total amount of food demand, t $f_{i}^{s}$ the total amount of food supply, t $f_{i}^{l-s}$ the food resource from local supply, t $f_{i}^{im-s}$ the food resource imported from other provinces in China, t $L_{c}^{local}$ the local land demand of crop $c$, ha $e_{j}^{dem}$ urban demand of energy j, tce $e_{j}^{l}$ energy resource from local supply, tce $e_{j}^{im}$ energy resource imported from other provinces in China, tce $e_{j}^{household-dem}$ energy demand from household, tce $e_{j}^{economic-dem}$ energy demand from economic system, tce $ELD^{dem}$ the total amount of electricity demand, 10^8^ kWh $ELD^{l}$ the electricity from local supply, 10^8^ kWh $ELD^{im}$ the electricity imported from other provinces in China, 10^8^ kWh $ELD^{coal-el}$ the coal fired power generation, 10^8^ kWh $ELD^{ng-el}$ the natural gas power generation, 10^8^ kWh $ELD^{renew-el}$ renewable energy generation, 10^8^ kWh $Heat^{dem}$ urban heat demand, 10^10^KJ $Heat^{l}$ heat from local supply, 10^10^KJ $Heat^{im}$ heat imported from other provinces in China, 10^10^KJ $M_{renew-el}^{l}$ the local renewable energy, MW $W_{k}^{withdrawal}$ water withdrawal, 10^8^ m^3^ $W^{use}$ the total amount of water use, 10^8^ m^3^ $W_{\text{gr}}$ the usage of groundwater, 10^8^ m^3^ $W_{sur}$ the usage of surface water, 10^8^ m^3^ $W_{ra}$ the usage of rainwater, 10^8^ m^3^ $W_{reuse}$ the usage of reclaimed water, 10^8^ m^3^ $W_{diversion}$ the usage of water diversion, 10^8^ m^3^ $cod_{k}$ the COD of wastewater, g COD/kg $W_{k}$ the amount of wastewater, 10^8^ m^3^ $cod'$ the COD of the treated wastewater from the treatment plant, g COD/kg $W'$ the amount of treated wastewater from the treatment plant, 10^8^ m^3^ |
| --- |

# **6. Key objective functions and constrains in this work**

Owing to the different nature of these resources, it is essential to choose a unified quantity for integrated optimization. Exergy defined as the maximum amount of work which can be produced by a system, could provide a unified way to measure various natural and human made resources with solid scientific basis (19,20). The scarcity and usefulness of exergy have been well proved by the global thermodynamics (21). Considering the life cycle process of FEW consumption, cumulative exergy consumption ($CExC$) is used in individual subsystem and integrated system optimization modelling. In the light of various demands and constraints in urban system, minimizing the total cumulative exergy consumption is a better choice to meet such demands and reduce resource consumption.

**Minimize objective function:**

$CExC_{total}^{scenario}=CExF^{scenario}+CExE^{scenario}+CExW^{scenario}$ (1)

$\begin{matrix} CExF^{scenario}=\sum_{i=1}^{m} ex_{i}f_{i}^{l}+\sum_{i=1}^{m} ex_{i}f_{i}^{im}+\sum_{c\in i,i=1}^{m} ex^{fertilizer}(\varepsilon_{c}^{fertilizer,l}f_{c}^{l}+\varepsilon_{c}^{fertilizer,im}f_{c}^{im}) \\ +\sum_{c\in i,i=1}^{m} ex^{pesticide}(\varepsilon_{c}^{pesticide,l}f_{c}^{l}+\varepsilon_{c}^{pesticide,im}f_{c}^{im})+\sum_{c\in i,i=1}^{m} ex^{plastic}(\varepsilon_{c}^{plastic,l}f_{c}^{l}+\varepsilon_{c}^{plastic,im}f_{c}^{im}) \\ +\sum_{c\in i,i=1}^{m} ex^{feed}(\varepsilon_{livestock}^{feed,l}f_{livestock}^{l}+\varepsilon_{livestock}^{feed,im}f_{c}^{im}) \end{matrix}$ (2)

$CExE^{scenario}=\sum_{j=1}^{n} ex_{j}(e_{j}^{l}+e_{j}^{im})$ (3)

$CExW^{scenario}=\sum_{k=1}^{o} (ex^{el}\varepsilon_{k}^{el}+ex^{ch}\varepsilon_{k}^{ch})w_{k}+ex^{r}\varepsilon_{ra}^{r}w_{ra}$ (4)

where $ex_{i}$, $ex^{fertilizer}$, $ex^{pesticide}$, $ex^{plastic}$ and $ex^{feed}$are the exergy coefficient (PJ/Mt) of food$i$, fertilizer, pesticide, plastic and feed, respectively. $ex_{j}$is the exergy coefficient (PJ/Mt, PJ/10^8^m^3^, PJ/10^8^kWh) of energy$j$.

**Subject to the key constraints within FEW subsystems**

1) Urban food supply-demand balance

$\sum_{i=1}^{m} f_{i}^{l-s}+\sum_{i=1}^{m} f_{i}^{im-s}=\sum_{i=1}^{m} f_{i}^{dem}$ (5)

$f_{i}^{s}=f_{i}^{p}(1-\varepsilon_{i}^{loss}-\varepsilon_{i}^{waste})$ (6)

$f^{dem}=\sum_{i=1}^{m} x_{i}$ (7)

2) the Chinese dietary guidelines constraint

$DRI_{i}^{min}\leq x_{i}\leq DRI_{i}^{max}$ (8)

$\sum_{i=1}^{m} f_{i}^{min}\times p\times365\leq f^{dem}\leq\sum_{i=1}^{m} f_{i}^{max}\times p\times365$ (9)

3) Land availability constraint

$\sum_{c\in i,i=1}^{m} L_{c}^{local}\leq L_{c,current}^{local}$ (10)

4) Urban energy supply-demand balance

$e_{j}^{l}+e_{j}^{im}=e_{j}^{dem}$ (11)

5) Urban energy demand constraint

$e_{j}^{dem}=e_{j}^{household-dem}+e_{j}^{economic-dem}$ (12)

6) Electricity supply-demand balance

$ELD^{dem}=ELD^{l}+ELD^{im}$ (13)

7) Electricity production constraint

$ELD^{dem}=ELD^{coal-el}+ELD^{ng-el}+ELD^{renew-el}$ (14)

8) Coal availability for power generation

$ELD^{coal-el}\leq ELD_{current}^{coal-el}$ (15)

9) Natural gas availability for power generation

$ELD_{current}^{ng-el}\leq ELD^{ng-el}$ (16)

10) Renewable energy availability constraint

$ELD_{current}^{renew-el}\leq ELD^{renew-el}$ (17)

11) Heat supply-demand balance

$Heat^{l}+Heat^{im}=Heat^{dem}$ (18)

12) Raw material availability constraint

$M_{r}=M_{r}^{direct}+M_{r}^{transfer}$ (19)

$\sum_{r\in j,j=1}^{n} M_{r}^{l}\leq M_{r}^{l,av}$ (20)

$M_{renew-el}^{l}\leq M_{renew-el}^{installed}$ (21)

13) Biomass energy production availability

$M_{bio}^{av}\leq\lambda_{c}A_{c}^{local}H_{c}$ (22)

14) Electricity conservation from raw material availability

$\sum_{r\in j,j=1}^{n} ELD^{r-el}=\sum_{r\in j,j=1}^{n} \eta_{r}^{el}M_{r}$ (23)

15) Urban water supply-demand balance

$\sum_{k=1}^{o} W_{k}^{withdrawal}=W^{use}$ (24)

16) Water supply constraint

$W_{k}^{withdrawal}\leq W_{k}^{volume}$ (25)

17) Water use type

$W^{use}=W_{\text{gr}}+W_{sur}+W_{ra}+W_{reuse}+W_{diversion}$ (26)

18) Water source availability constraint

$W_{gr}\leq W_{gr}^{current}$ (27)

$W_{sur}\leq W_{sur}^{current}$ (28)

$W_{ra}\leq W_{ra}^{planning}$ (29)

$W_{diversion}\leq W_{diversion}^{planning}$ (30)

19) Concentration balance with respect to chemical oxygen demand (COD) levels

$(1-Ref_{cod})\sum_{k=1}^{o} cod_{k}W_{k}=cod'W'$ (31)

$cod_{w}^{t}=\sum_{k=1}^{o} cod_{k}W_{k}$ (32)

$\sum_{k=1}^{o} cod_{k}W_{k}\leq cod_{\alpha}W^{dem}$ (33)

where $f_{i}^{dem}$, $f_{i}^{s}$, $f_{i}^{l-s}$ and $f_{i}^{im-s}$are food demand, food supply, and food resource from local supply and imported from other provinces in China, respectively. $i$ is food $i$, and $i\in m$. $f_{i}^{p}$ is the food production. $\varepsilon_{i}^{loss}$ and $\varepsilon_{i}^{waste}$ are the coefficients of loss and waste in food $i$, respectively. $x_{i}$ is the food demand of different types. $DRI_{i}^{min}$ and $DRI_{i}^{max}$ are the minimum and maximum food intakes based on the Chines Dietary Guidelines. $p$ is urban population. $L_{c}^{local}$ and $L_{c,current}^{local}$ are the local land demand and current land use of crop $c$, respectively. $e_{j}^{dem}$, $e_{j}^{l}$ and $e_{j}^{im}$ are urban demand of energy $j$, energy resource from local supply and imported from other provinces in China, respectively. $e_{j}^{household-dem}$ and $e_{j}^{economic-dem}$ are energy demand from household and economic system, respectively. $ELD^{dem}$, $ELD^{l}$ and $ELD^{im}$ represent electricity demand, electricity from local supply and imported from other provinces in China, respectively. $ELD^{coal-el}$, $ELD^{ng-el}$, and $ELD^{renew-el}$ denote coal fired power generation, natural gas power generation and renewable energy, respectively. $ELD_{current}^{coal-el}$, $ELD_{current}^{ng-el}$ and $ELD_{current}^{renew-el}$ are the current electricity supply from coal fired, natural fired and renewable energy, respectively. $Heat^{dem}$, $Heat^{l}$ and $Heat^{im}$ are urban heat demand, heat from local supply and imported from other provinces in China, respectively. $M_{r}$, $M_{r}^{direct}$, $M_{r}^{transfer}$ and $M_{r}^{l,av}$ are raw materials of energy resource (including biomass, solar and wind resources), direct and transferred raw materials, and available quantify of raw materials, respectively. $M_{renew-el}^{l}$ and $M_{renew-el}^{installed}$ are the local renewable energy and installed capacity, respectively. $M_{bio}^{av}$ is the available collection of biomass raw materials (crop straw) in urban system. $\lambda_{c}$, $A_{c}^{local}$ and $H_{c}$ are the straw amount per unit yield of crop $c$, demand of crop $c$, and the collection and utilization rate of crop straw, respectively. $\eta_{r}^{el}$ is the electricity conservation from raw material $r$. $W_{k}^{withdrawal}$, $W^{use}$ and $W_{k}^{volume}$are water withdrawal, water use and available water resource, respectively. $W_{\text{gr}}$, $W_{sur}$, $W_{ra}$, $W_{reuse}$ and $W_{diversion}$ are the groundwater, surface water, rainwater, reclaimed water and water diversion, respectively. $W_{gr}^{current}$ and $W_{sur}^{current}$ are the current use of groundwater and surface water, respectively. $W_{ra}^{planning}$ and $W_{diversion}^{planning}$ are the availability of rainwater and water diversion in urban planning, respectively. $Ref_{cod}$ is the COD removal efficiency of the treatment plant. $cod_{k}$ and $W_{k}$ are the COD of wastewater and the amount of wastewater, respectively. $cod'$and $W'$ are the COD of the treated wastewater from the treatment plant and the amount of treated wastewater from the treatment plant, respectively.

**Subject to the key constraints cross-subsystems**

20) Energy requirements

$e_{food}^{dem}=\sum_{i=1}^{m} e_{food,i}^{production}+\sum_{i=1}^{m} e_{food,i}^{processing}+\sum_{i=1}^{m} e_{food,i}^{transport}+\sum_{i=1}^{m} e_{food,i}^{cooking}$ (34)$e_{food}^{production}=\sum_{i=1}^{m} ELD_{food,i}^{production}+\sum_{i=1}^{m} Oil_{food,i}^{production}+\sum_{i=1}^{m} Coal_{food,i}^{production}$ (35)$ELD_{w}^{total}=\sum_{k=1}^{o} \xi_{k}^{eld}W_{k}^{use}$ (36)

$ELD_{w}^{total}=\xi_{sur}W_{sur}^{use}+\xi_{gr}W_{gr}^{use}+\xi_{ra}W_{ra}^{use}+\xi_{reuse}W_{reuse}^{use}+\xi_{diversion}W_{diversion}^{use}$(37)

$e_{j}^{household-dem}=\beta_{j,per}^{urban-household}\times p^{urban}+\beta_{j,per}^{rural-household}\times p^{rural}$ (38)$e_{j}^{economic-dem}=\mu_{j}^{sector}GDP^{sector}$ (39)

21) Water requirements

$W^{dem}=W^{agriculture}+W^{industry}+W^{services}+W^{household}+W^{ecology}$ (40)

where $e_{food}^{dem}$, $e_{food,i}^{production}$, $e_{food,i}^{processing}$, $e_{food,i}^{transport}$ and $e_{food,i}^{cooking}$ are the energy demand of food subsystem, food production, processing, transport and cooking, respectively. $ELD_{food,i}^{production}$, $Oil_{food,i}^{production}$ and $Coal_{food,i}^{production}$ are the use of electricity, oil and coal in food production process, respectively. $ELD_{w}^{total}$, $\xi_{k}^{eld}$ and $W_{k}^{use}$are the total electricity use of water subsystem, electricity use coefficient of different water types, and water use, respectively. $e_{j}^{household-dem}$ and $e_{j}^{economic-dem}$are energy demand of household and economic system, respectively. $\beta_{j,per}^{urban-household}$ and $\beta_{j,per}^{rural-household}$ are the energy demand coefficient of per capita urban and rural household, respectively. $p^{urban}$ and $p^{rural}$ are urban and rural population, respectively. $\mu_{j}^{sector}$ and $GDP^{sector}$ are energy use intensity and GDP of each sector, respectively. $W^{agriculture}$, $W^{industry}$, $W^{services}$, $W^{household}$ and $W^{ecology}$ are water use of agriculture, industry, services, household and ecological environment, respectively.

# **7. Data sources and the key parameters of each resource in the different life cycle processes^i^**

# **Supplementary Table S11.** The key materials and energy input intensities of grain in the different life cycle processes

| **Key processes** | | **Mathematical equations** | **Data description** |
| --- | --- | --- | --- |
| Production | Fertilizer | FE1=CS1/YI1/PR1 | FE1: the total amount of fertilizer use (t)  CS1: the cost of fertilizer (yuan/mu)  YI1: the grain yield (kg/mu)  PR1: fertilizer price in 2017 (yuan/kg) |
|  | Pesticides | PE1=CS2/YI1/PR2 | PE1: the total amount of pesticides use (t)  CS2: the cost of pesticides (yuan/mu)  YI1: the grain yield (kg/mu)  PR2: pesticides price in 2017 (yuan/kg) |
|  | plastic | PL1=CS3/YI1/PR3 | PL1: the total amount of plastic use (t)  CS3: the cost of plastic (yuan/mu)  YI1: the grain yield (kg/mu)  PR3: plastic price in 2017 (yuan/kg) |
|  | Diesel | DI1=CS4/YI1/PR4 | DI1: the total amount of diesel use (t)  CS4: the cost of diesel (yuan/mu)  YI1: the grain yield (kg/mu)  PR4: diesel price in 2017 (yuan/kg) |
| Processing | Electricity | 9.1kWh/t | — |
| Transport | Energy | EC1=EI1×GR×Distance | EC1: the total oil consumption (t)  EI1: energy consumption of per kg food (L/km)  GR: the amount of grain supply from other regions  Distance: the distance between supply region and Beijing city |
| Cooking | Electricity | EL1=F1×EI2×h | EL1: the electricity consumption  F1: amount of a meal (kg)  EI2: energy intensity (kWh)  h: cooking time (h) |

Note:^i^Date for various cost were taken from the Statistic Yearbook (19,22,23).

# **Supplementary Table S12.** The key materials and energy input intensities of vegetables in the different life cycle processes

| **Key processes** | | **Mathematical equations** | **Data description** |
| --- | --- | --- | --- |
| Production | Fertilizer | FE2=CS1/YI2/PR1 | FE2: the total amount of fertilizer use (t)  CS1: the cost of fertilizer (yuan/mu)  YI2: the vegetables yield (kg/mu)  PR1: fertilizer price in 2017 (yuan/kg) |
|  | Pesticides | PE2=CS2/YI2/PR2 | PE2: the total amount of pesticides use (t)  CS2: the cost of pesticides (yuan/mu)  YI2: the vegetables yield (kg/mu)  PR2: pesticides price in 2017 (yuan/kg) |
|  | plastic | PL2=CS3/YI2/PR3 | PL2: the total amount of plastic use (t)  CS3: the cost of plastic (yuan/mu)  YI2: the vegetables yield (kg/mu)  PR3: plastic price in 2017 (yuan/kg) |
|  | Diesel | DI2=CS4/YI2/PR4 | DI2: the total amount of diesel use (t)  CS4: the cost of diesel (yuan/mu)  YI2: the vegetables yield (kg/mu)  PR4: diesel price in 2017 (yuan/kg) |
| Transport | Energy | EC2=EI1×VE×Distance | EC2: the total oil consumption (t)  EI1: energy consumption of per kg food (L/km)  GR: the amount of vegetables supply from other regions  Distance: the distance between supply region and Beijing city |
| Cooking | Natural gas | NS1=F2×EI2×h | NS1: nature gas consumption  F2: amount of a meal (kg)  EI2: energy intensity per hour (kWh)  h: cooking time (h) |

# **Supplementary Table S13.** The key materials and energy input intensities of fruits in the different life cycle processes

| **Key processes** | | **Mathematical equations** | **Data description** |
| --- | --- | --- | --- |
| Production | Fertilizer | FE3=CS1/YI3/PR1 | FE2: the total amount of fertilizer use (t)  CS1: the cost of fertilizer (yuan/mu)  YI2: the vegetables yield (kg/mu)  PR1: fertilizer price in 2017 (yuan/kg) |
|  | Pesticides | PE3=CS2/YI3/PR2 | PE2: the total amount of pesticides use (t)  CS2: the cost of pesticides (yuan/mu)  YI2: the vegetables yield (kg/mu)  PR2: pesticides price in 2017 (yuan/kg) |
|  | plastic | PL3=CS3/YI3/PR3 | PL2: the total amount of plastic use (t)  CS3: the cost of plastic (yuan/mu)  YI2: the vegetables yield (kg/mu)  PR3: plastic price in 2017 (yuan/kg) |
|  | Diesel | DI3=CS4/YI3/PR4 | DI2: the total amount of diesel use (t)  CS4: the cost of diesel (yuan/mu)  YI2: the vegetables yield (kg/mu)  PR4: diesel price in 2017 (yuan/kg) |
| Transport | Energy | EC3=EI1×FR×Distance | EN1: the total oil consumption (t)  EI1: energy consumption of per kg food (L/km)  FR: the amount of fruits supply from other regions  Distance: the distance between supply region and Beijing city |

# **Supplementary Table S14.** The key materials and energy input intensities of animal-sourced foods in the different life cycle processes in Beijing

| **Key processes**  **Food types** | **Production** | | | | | **Processing** | **Transport** | **Cooking** |
| --- | --- | --- | --- | --- | --- | --- | --- | --- |
|  | C-feed | Roughage | Electricity | Coal | Water | Electricity | Energy | Natural gas |
| **Pork** | 3.2532 | 2.2858 | 0.1110 | 0.00005 | 0.4050 | 0.0518 | — | 0.5467 |
| **Beef** | 0.1333 | 0 | 0.0248 | 0 | 0.4850 | 0.0518 | — | 0.5467 |
| **Mutton** | 0.3948 | 0 | 0.1853 | 0 | 0.4520 | 0.0518 | — | 0.5467 |
| **Poultry** | 1.9200 | 1.3440 | 0.0700 | 0.00002 | 0.2810 | 0.0518 | — | 0.5467 |
| **Eggs** | 2.6171 | 1.8320 | 0.05691 | 0 | 0.4050 | — | — | 0.4556 |
| **Dairy** | 0.5477 | 0.3834 | 0.0304 | 0.00002 | 0.1450 | — | — | — |
| **Aquatic products** | 1.2906 | 0 | 0.96576 | 0.00017 | 0.5620 | 0.0518 | — | 0.0683 |

Note: the feed intensity was kg/kg; electricity intensity was kWh/kg; coal intensity was t/kg; water intensity was m^3^/kg.

# **Supplementary Table S15.** The key materials and energy input intensities of animal-sourced foods in the different life cycle processes in Inner Mongolia

| **Key processes**  **Food types** | **Production** | | | | | **Processing** | **Transport** |
| --- | --- | --- | --- | --- | --- | --- | --- |
|  | C-feed | Roughage | Electricity | Coal | Water | Electricity | Energy |
| **Pork** | 4.7944 | 3.9146 | 0.1950 | 0.0003 | 0.4050 | 0.0518 | ECi=EIi×FR×Distance |
| **Beef** | 0.5204 | 0 | 0.3659 | 0 | 0.4850 | 0.0518 |  |
| **Mutton** | 0.7618 | 0 | 0.8951 | 0 | 0.4520 | 0.0518 |  |
| **Poultry** | 2.2487 | 1.5299 | 0.1339 | 0.0003 | 0.2810 | 0.0518 |  |
| **Eggs** | 2.3100 | 1.6161 | 0.0603 | 0.00002 | 0.4050 | — |  |
| **Dairy** | 0.6085 | 0.4169 | 0.0435 | 0.00003 | 0.1450 | — |  |
| **Aquatic products** | — | — | — | — | — | — | — |

Note: the feed intensity was kg/kg; electricity intensity was kWh/kg; coal intensity was t/kg; water intensity was m^3^/kg.

# **Supplementary Table S16.** The key materials and energy input intensities of animal-sourced foods in the different life cycle processes in Henan province

| **Key processes**  **Food types** | **Production** | | | | | **Processing** | **Transport** |
| --- | --- | --- | --- | --- | --- | --- | --- |
|  | C-feed | Roughage | Electricity | Coal | Water | Electricity | Energy |
| **Pork** | 4.2647 | 2.9573 | 0.0958 | 0 | 0.4050 | 0.0518 | ECi=EIi×FR×Distance |
| **Beef** | 0.1582 | 0 | 0.0333 | 0 | 0.4850 | 0.0518 |  |
| **Mutton** | 0.5533 | 0 | 0.2538 | 0 | 0.4520 | 0.0518 |  |
| **Poultry** | 2.2417 | 1.6027 | 0.0872 | 0 | 0.2810 | 0.0518 |  |
| **Eggs** | 2.2603 | 1.5409 | 0.0466 | 0.00002 | 0.4050 | — |  |
| **Dairy** | — | — | — | — | — | — |  |
| **Aquatic products** | — | — | — | — | — | — | — |

Note: the feed intensity was kg/kg; electricity intensity was kWh/kg; coal intensity was t/kg; water intensity was m^3^/kg.

# **Supplementary Table S17.** The key materials and energy input intensities of animal-sourced foods in the different life cycle processes in Hebei province

| **Key processes**  **Food types** | **Production** | | | | | **Processing** | **Transport** |
| --- | --- | --- | --- | --- | --- | --- | --- |
|  | C-feed | Roughage | Electricity | Coal | Water | Electricity | Energy |
| **Pork** | 3.7465 | 2.6704 | 0.0958 | 0 | 0.4050 | 0.0518 | ECi=EIi×FR×Distance |
| **Beef** | 0.1493 | 0 | 0.0333 | 0 | 0.4850 | 0.0518 |  |
| **Mutton** | 0.4507 | 0 | 0.2538 | 0 | 0.4520 | 0.0518 |  |
| **Poultry** | 2.4819 | 1.7745 | 0.1119 | 0.0002 | 0.2810 | 0.0518 |  |
| **Eggs** | 2.3212 | 1.6207 | 0.0504 | 0 | 0.4050 | — |  |
| **Dairy** | — | — | — | — | — | — |  |
| **Aquatic products** | — | — | — | — | — | — | — |

Note: the feed intensity was kg/kg; electricity intensity was kWh/kg; coal intensity was t/kg; water intensity was m^3^/kg.

# **Supplementary Table S18.** The key materials and energy input intensities of animal-sourced foods in the different life cycle processes in Shandong province

| **Key processes**  **Food types** | **Production** | | | | | **Processing** | **Transport** |
| --- | --- | --- | --- | --- | --- | --- | --- |
|  | C-feed | Roughage | Electricity | Coal | Water | Electricity | Energy |
| **Aquatic products** | 1.2906 | 0 | 0.9658 | 0.0002 | 0.5620 | 0.0683 | ECi=EIi×FR×Distance |

Note: the feed intensity was kg/kg; electricity intensity was kWh/kg; coal intensity was t/kg; water intensity was m^3^/kg.

# **Supplementary Table S19.** Water use of plant-based foods in the different life cycle processes (24)

| **Food type** | **Regions** | **Green water** | **Blue water** | **Grey water** |
| --- | --- | --- | --- | --- |
| Wheat | Beijing | 0.7921 | 0.3923 | 0.2600 |
|  | Hebei | 0.5370 | 0.6024 | 0.2628 |
|  | Shandong | 0.6403 | 0.5413 | 0.2674 |
| Rice | Beijing | 0.6042 | 0.8164 | 0.2866 |
|  | Heilongjiang | 0.7565 | 0.6317 | 0.3377 |
|  | Jilin | 0.8128 | 0.6705 | 0.3373 |
| Vegetables | Beijing | 0.2506 | 0.0093 | 0.2006 |
|  | Hebei | 0.2401 | 0.0158 | 0.1882 |
|  | Shandong | 0.2419 | 0.0057 | 0.1634 |
| Fruits | Beijing | 1.3306 | 0.1283 | 0.5468 |
|  | Hebei | 1.4072 | 0.0554 | 0.5427 |
|  | Shandong | 1.4235 | 0.0384 | 0.4759 |
|  | Guangdong | 1.5564 | 0.0014 | 0.3873 |

Note: the unit of water intensity was m^3^/kg.

# **Supplementary Table S20.** The water footprint coefficient of each energy category^j^

| **Energy type** | **Footprint coefficient** | **Energy type** | **Footprint coefficient** |
| --- | --- | --- | --- |
| Raw coal | 0.35-2.30 (m^3^/t) | Thermal power | 2.92-3.00 (m^3^/MWh) |
| Crude oil | 1.05-8.00 (m^3^/t) | Heating | 0.37-0.50 (m^3^/GJ) |
| Oil products | 0.75 (m^3^/t) | Biomass | 2.10-2.70 (m^3^/MWh) |
| Natural gas | 0.002-0.008 (m^3^/m^3^) | Solar power | 0 (m^3^/MWh) |
| Hydropower | 0 (m^3^/MWh) | Wind power | 0 (m^3^/MWh) |

Note: ^j^water footprint coefficient were based on “industry water consumption quota in the main energy producing provinces and the literature research (24-26).

# **Supplementary Table S21.** The energy footprint coefficient of each water category^k^

| **Key processes** | **Footprint coefficient (kWh/m^3^)** |
| --- | --- |
| Ground water withdrawal | 0.44 |
| Surface water withdrawal | 0.14 |
| Water supply | 0.35 |
| Wastewater treatment | 0.24 |

Note: ^k^footprint coefficients were referred by the literature review (27-31).

# **Supplementary Table S22.** Exergy coefficient of various resources (32,33)

| **Item** | **Exergy coefficient** | **Unit** |
| --- | --- | --- |
| Coal | 22.16 | PJ/Mt |
| Oil/petroleum product | 44.32 | PJ/Mt |
| Natural gas | 4.13 | PJ/10^8^m^3^ |
| Electricity | 0.36 | PJ/10^8^kWh |
| District heat | 0.20 | PJ/10^15^J |
| Rice | 15.80 | PJ/Mt |
| Wheat | 13.90 | PJ/Mt |
| Vegetable | 1.90 | PJ/Mt |
| Fruit | 1.90 | PJ/Mt |
| Meat | 4.600 | PJ/Mt |
| Milk | 4.9 | PJ/Mt |
| Egg | 6.10 | PJ/Mt |
| Aquatic products | 5.800 | PJ/Mt |
| Fertilizer | 37.1 | PJ/Mt |
| Plastic | 32.50 | PJ/Mt |
| Pesticide | 7.52 | PJ/Mt |
| Fodder | 13.90 | PJ/Mt |

# **Supplementary Table S23.** GHG emission coefficient of food resource in different life cycle processes^l^

| **Item** | **Production emission** | **Use emission** | **Burning emission** |
| --- | --- | --- | --- |
| Fertilizer | 1.0595 (kg CO_2_-eq/kg) | 0.0025 (kg CO_2_-eq/kg) | — |
| Pesticide | 9.7000 (kg CO_2_-eq/kg) | — | — |
| Plastic | 0.7910(kg CO_2_-eq/kg) | — | — |
| Diesel | 0.4940(kg CO_2_-eq/kg) | — | 3.1317 (kg CO_2_-eq/kg) |
| Concentrated feed | 0.6450 (kg CO_2_-eq/kg) | — | — |
| Roughage | 0.1240 (kg CO_2_-eq/kg) | — | — |
| Enteric fermentation (Beef) | 2.7696 (kg CO_2_-eq/kg) | — | — |
| Enteric fermentation (Mutton) | 3.6127 (kg CO_2_-eq/kg) | — | — |
| Enteric fermentation (Cow) | 0.3093 (kg CO_2_-eq/kg) | — | — |
|  | **Direct emission** | **Indirect emission** | — |
| Manure emission (Beef) | 0.0454 (kg CO_2_-eq/kg) | 0.1612 (kg CO_2_-eq/kg) | — |
| Manure emission (Mutton) | 0.1188 (kg CO_2_-eq/kg) | 0.1576 (kg CO_2_-eq/kg) | — |
| Manure emission (Cow) | 0.0886 (kg CO_2_-eq/kg) | 0.1169 (kg CO_2_-eq/kg) | — |
|  | **Production emission** | **Burning emission** | — |
| Coal | 0.4870 (kg CO_2_-eq/kg) | 1.4723 (kg CO_2_-eq/kg) | — |
| Thermal power (coal) | 0.9500 (kg CO_2_-eq/kWh) | — | — |
| Heating (coal) | 0.4870 (kg CO_2_-eq/kg) | 0.1290 (kg CO_2_-eq/10^3^kJ) | — |
| Thermal power (Natural gas) | 0.5230 (kg CO_2_-eq/kWh) | — | — |
| Heating (Natural gas) | 0.2500 (kg CO_2_-eq/kg) | 0.0109 (kg CO_2_-eq/10^3^kJ) | — |
| Hydropower | 0.0042 (kg CO_2_-eq/kWh) | — | — |
| Wind power | 0.0204 (kg CO_2_-eq/kWh) | — | — |
| Solar power | 0.0756 (kg CO_2_-eq/kWh) | — | — |
| Gasoline | 0.6990 (kg CO_2_-eq/kg) | 3.1217 (kg CO_2_-eq/kg) | — |
| Kerosene | 0.4940 (kg CO_2_-eq/kg) | 3.1863 (kg CO_2_-eq/kg) | — |
| Diesel oil | 0.4840 (kg CO_2_-eq/kg) | 3.1863 (kg CO_2_-eq/kg) | — |
| Fuel oil | 0.4840 (kg CO_2_-eq/kg) | 3.1270 (kg CO_2_-eq/kg) | — |
| Imported power | 0.8790 (kg CO_2_-eq/kWh) | — | — |

Note: ^l^these parameters were all taken from the Ecoinvent database version 3.0, and the characterization factors of China’s practices were primarily used. The life-cycle impact assessment method used for the study is ReCiPe Midpoint, which is a standard in scientific LCA research that indicates how different environmental mechanisms are affected by various anthropogenic emissions over a 100 year time horizon for most mechanisms and without considering uncertain long-term effects.

# **Supplementary Table S24.** Prices of various food, energy and water resources in Beijing, 2017^m^

| **Item** | **Price (CNY/kg)** | **Item** | **Price** |
| --- | --- | --- | --- |
| Wheat | 4.12 | Coal | 535.00 (CNY/t) |
| Rice | 5.31 | Petroleum | 8421.86 (CNY/t) |
| Vegetables | 5.82 | Natural gas | 2.73 (CNY/m^3^) |
| Fruit | 11.67 | Electricity | 0.39 (CNY/kWh) |
| Pork | 27.46 | Heating | 0.84 (CNY/10^10^J) |
| Beef | 72.23 | Biomass | 0.77 (CNY/kWh) |
| Mutton | 78.22 | Wind power | 0.57 (CNY/kWh) |
| Poultry | 22.61 | Solar power | 0.94 (CNY/kWh) |
| Eggs | 10.49 | Hydropower | 0.26 (CNY/kWh) |
| Dairy | 20.98 | Water (residents) | 3.64 (CNY/m^3^) |
| Aquatic products | 26.15 | Wastewater treatment (residents) | 1.36 (CNY/m^3^) |
| — | — | Water (others) | 6.50 (CNY/m^3^) |
| — | — | Wastewater treatment (others) | 3.00 (CNY/m^3^) |

Note:^m^these prices were taken from the government’s website and Statistical Yearbook (34-36).

Beijing Municipal Commission of Development and Reform (coal). <http://fgw.beijing.gov.cn/gzdt/fgzs/mtbdx/bzwlxw/201912/t20191221_1394704.htm> Accessed 10 April 2021.

Beijing Municipal Commission of Development and Reform (petroleum products). <http://fgw.beijing.gov.cn/gzdt/tztg/202004/t20200417_1818894.htm> Accessed 10 April 2021.

Beijing Municipal Commission of Development and Reform (heating)

<http://fgw.beijing.gov.cn/fgwzwgk/zcgk/bwqtwj/201912/t20191226_1506645.htm> Accessed 10 April 2021.

Beijing Municipal Commission of Development and Reform (Natural gas)

<http://fgw.beijing.gov.cn/fgwzwgk/zcgk/bwqtwj/201912/t20191226_1506646.htm> Accessed 10 April 2021

Beijing Municipal Commission of Development and Reform (water)

<http://fgw.beijing.gov.cn/fgwzwgk/zcgk/bwqtwj/201912/t20191226_1506655.htm> Accessed 10 April 2021.

# **References**

1 Li Y, et al. 2015. Life cycle assessment of water supply alternatives in water-receiving areas of the South-to-North Water Diversion Project in China. *Water Res.* 89:9-19.

2 Zhao Y, et al. 2017. Energy reduction effect of the South-to-North Water Diversion Project in China. *Sci. Rep.* 7:15956.

3 Zhao Z, Zuo Y, Zillante JG. 2017. Transformation of water resource management: a case study of the South-to-North Water Diversion project. *J Clean. Prod.* 163:136-145.

4 Tilman D, et al. 2011. Global food demand and the sustainable intensification of agriculture. *Proc. Natl. Acad. Sci. USA.* 108:20260-20264.

5 Obersteiner M, et al. 2016. Assessing the land resource-food price nexus of the Sustainable Development Goals. *Sci. Adv.* 2:e1501499.

6 Van Oers L, De Koning A, Guinée JB. 2002. Abiotic resource depletion in LCA. Improving characterisation factors for abiotic resource depletion as recommended in the Dutch LCA handbook. Delft, The Netherlands.

7 Schlör H, Venghaus S, Hake JF. 2018. The FEW-Nexus city index – measuring urban resilience. *Appl. Energy* 210:382-392.

8 FAO. 2019. The state of food and agriculture 2019. Moving forward on food loss and waste reduction.

9 Xue L, et al. 2021. China’s food loss and waste embodies increasing environmental impacts. *Nat. Food* 2:519-528.

10 He P, et al. 2018. The environmental impacts of rapidly changing diets and their nutritional quality in China. *Nat. Sustain.* 1:122-127.

11 Beijing Municipal Commission of Development and Reform. 2016. The 13th Five-Year Plan for Beijing Municipal New and Renewable Energy Development. Beijing, China.

12 Johansson TB, et al. 2012. Global Energy Assessment — Toward a Sustainable Future. Cambridge University Press.

13 IEA. 2021. World Energy Outlook 2020.

14 Beijing Water Authority. 2016. The 13th Five-Year Plan for Beijing Municipal Water Development. Beijing, China.

15 Rothausen SGSA, Conway D. 2011. Greenhouse-gas emissions from energy use in the water sector. *Nat. Clim. Change* 1(4):201- 219.

16 Liang S, et al. 2019. Quantifying the urban food-energy-water nexus: the case of the Detroit Metropolitan Area. Environ. Sci. Tech. 53(2):779-788.

17 Xiong X, et al. 2020. Urban dietary changes and linked carbon footprint in China: a case study of Beijing. *J. Environ. Manage.* 255:109877.

18 Van Vuuren DP, et al. 2019. Integrated scenarios to support analysis of the food–energy–water nexus. *Nat. Sustain.* 2:1132-1141.

19 Valero A. 2006. Exergy accounting: capabilities and drawbacks. *Energy*, 31:164-180.

20 Sciubba E, Wall G. 2007. A brief commented history of exergy from the beginnings to 2004. *Int J Thermodyn* 10(1):1-26.

21 Chen GQ. 2005. Exergy consumption of the earth. *Ecol. Model.* 184(2-4):363-380.

22 Hoff H. 2011. Understanding the nexus. Background Paper for the Bonn 2011 Conference: the Water, Energy and Food Security Nexus. [https://uploads.water-energy-food.org/resources/SEI-Paper-Hoff-UnderstandingTheNexus-2011.pdf. Accessed 15 January 2022](https://uploads.water-energy-food.org/resources/SEI-Paper-Hoff-UnderstandingTheNexus-2011.pdf.%20Accessed%2015%20January%202022).

23 Beijing Municipal Bureau of Statistics. 2018. Beijing Statistical Yearbook. China Statistics Press, Beijing, China.

24 Mekonnen MM, Hoekstra AY. 2010. The green, blue and grey water footprint of crops and derived crop products. http://www.waterfootprint.org/Repors/Report147-WaterFootprintCrop-Vol1.pdf (2010).

25 Ministry of Agriculture and Rural Affairs of China. 2018. China Agricultural Yearbook. China Statistics Press, Beijing, China.

26 Macknick J, et al. 2012. Operational water consumption and withdrawal factors for electricity generating technologies: a review of existing literature. *Environ. Res. Lett.* 7:045802.

27 Peng W, et al. 2018. Managing China’s coal power plants to address multiple environmental objectives. *Nat. Sustain.* 1:693-701.

28 Qin Y, et al. 2018. Air quality-carbon-water synergies and trade-offs in China’s natural gas industry. *Nat. Sustain.* 1:505-511.

29 Wang JX, et al. 2012. China’s water-energy nexus: greenhouse-gas emissions from groundwater use for agriculture. *Environ. Res. Lett.* 7:014035.

30 Smith K, et al. 2016. Impact of urban water supply on energy use in China: a provincial and national comparison. *Mitig Adapt Strateg Glob Change* 21:1213-1233.

31 He Y, et al. 2019. Assessment of energy consumption of municipal wastewater treatment plants in China. *J Clean. Prod.* 228:399-404.

32 Madhu K, Pauliuk S, Dhathri S, Creutzig F. 2021. Understanding environmental trade-offs and resource demand of direct air capture technologies through comparative life-cycle assessment. *Nat. Energy* 6:1035-1044.

33 Chen GQ, Chen B. 2009. Extended-exergy analysis of the Chinese society. *Energy* 34(9):1127-1144.

34 National Bureau of Statistics of China. 2018. China Price Statistical Yearbook. China Statistics Press, Beijing, China.

35 National Energy Administration. 2017. National electricity price supervision notification 2017.

36 National Bureau of Statistics of China. 2018. China Yearbook of Agricultural Price Survey. China Statistics Press, Beijing, China.
